# Supplementary material for: Spatial and temporal heterogeneity in human mobility patterns in Holocene Southwest Asia and the East Mediterranean
Source: Curr Biol. 2023 Jan 9;33(1):41–57.e15. doi: 10.1016/j.cub.2022.11.034 (PMC9839366; doi:10.1016/j.cub.2022.11.034)
Supplement: Document S1. Figures S1–S5 [file mmc1.pdf]

Current Biology, Volume *33*

Supplemental Information

Spatial and temporal heterogeneity in human mobility patterns in Holocene Southwest Asia and the East Mediterranean

Dilek Koptekin, Eren Yüncü, Ricardo Rodríguez-Varela, N. Ezgi Altınışık, Nikolaos Psonis, Natalia Kashuba, Sevgi Yorulmaz, Robert George, Duygu Deniz Kazancı, Damla Kaptan, Kanat Gürün, Kıvılcım Başak Vural, Hasan Can Gemici, Despoina Vassou, Evangelia Daskalaki, Cansu Karamurat, Vendela K. Lagerholm, Ömür Dilek Erdal, Emrah Kırdök, Aurelio Marangoni, Andreas Schachner, Handan Üstündağ, Ramaz Shengelia, Liana Bitadze, Mikheil Elashvili, Eleni Stravopodi, Mihriban Özbaşaran, Güneş Duru, Argyro Nafplioti, C. Brian Rose, Tuğba Gencer, Gareth Darbyshire, Alexander Gavashelishvili, Konstantine Pitskhelauri, Özlem Çevik, Osman Vuruşkan, Nina Kyparissi-Apostolika, Ali Metin Büyükkarakaya, Umay Oğuzhanoğlu, Sevinç Günel, Eugenia Tabakaki, Akper Aliev, Anar Ibrahimov, Vaqif Shadlinski, Adamantios Sampson, Gülşah Merve Kılınç, Çiğdem Atakuman, Alexandros Stamatakis, Nikos Poulakakis, Yılmaz Selim Erdal, Pavlos Pavlidis, Jan Storå, Füsun Özer, Anders Götherström, and Mehmet Somel

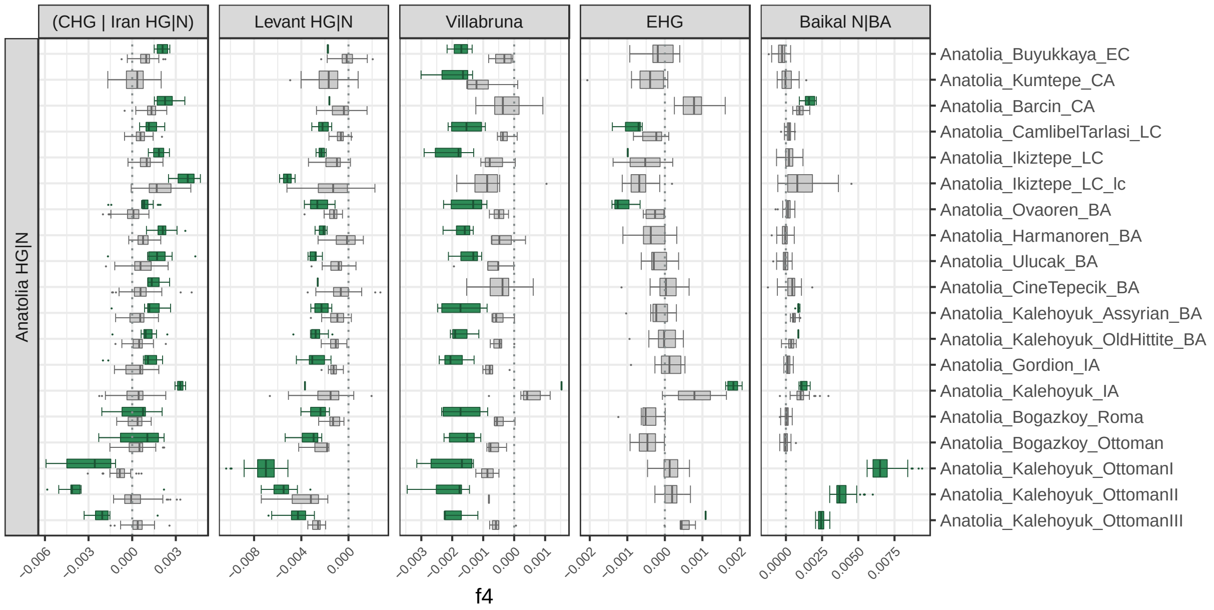

Figure S1: Genetic affinities of post-Neolithic Anatolian populations, **related to Table S3-S4 and Figure 3**. Results from the model f_4*(YRI, Test; Anatolia HG|N, Post Neolithic Anatolian)*, where *Test* refers to the South Caucasus/Iran or Levant pre-Neolithic/Neolithic populations, Villabruna, EHG and Baikal populations (Table S3). The Dataset 1 (STAR Methods) was used in the analysis. Boxplots show all f_4 statistics calculated by at least 10K overlapping SNPs. Green colour boxplots show nominally significant f_4-statistics with $|Z| > 3$ and grey colour boxplots show non-significant f_4-statistics with $|Z| < 3$.

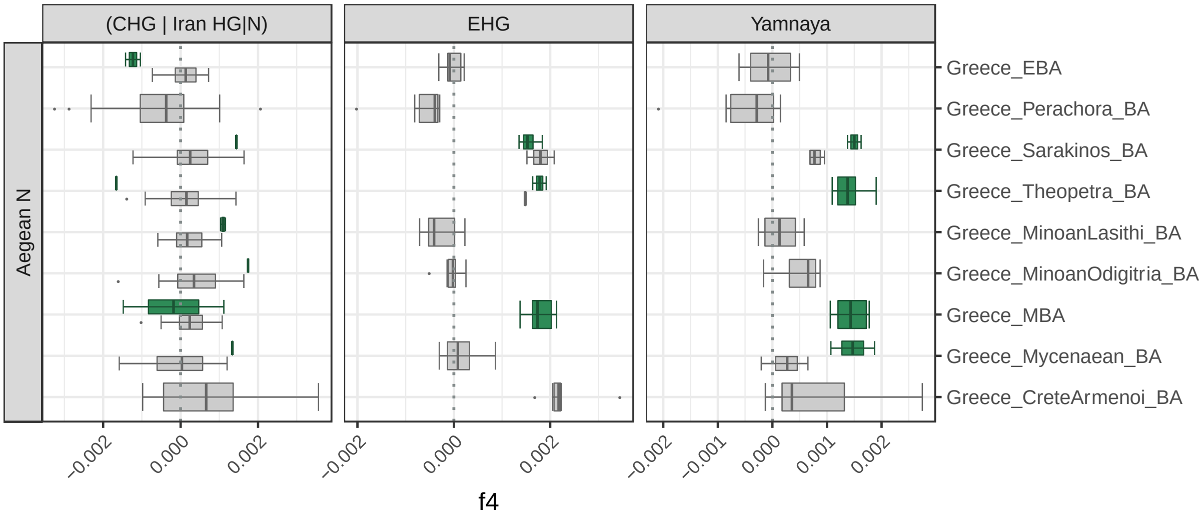

Figure S2: Genetic affinities of post-Neolithic Aegean populations, related to Table S3-S4 and Figure 3. Results from the model f_4*(YRI, Test; Aegean N, Post Neolithic Aegean)*, where *Test* refers to the South Caucasus/Iran pre-Neolithic/Neolithic populations, EHG and Yamnaya populations (Table S3). The Dataset 1 (STAR Methods) was used in the analysis. Boxplots show all f_4 statistics calculated by at least 10K overlapping SNPs. Green colour boxplots show nominally significant f_4-statistics with $|Z| > 3$ and grey colour boxplots show non-significant f_4-statistics with $|Z| < 3$.

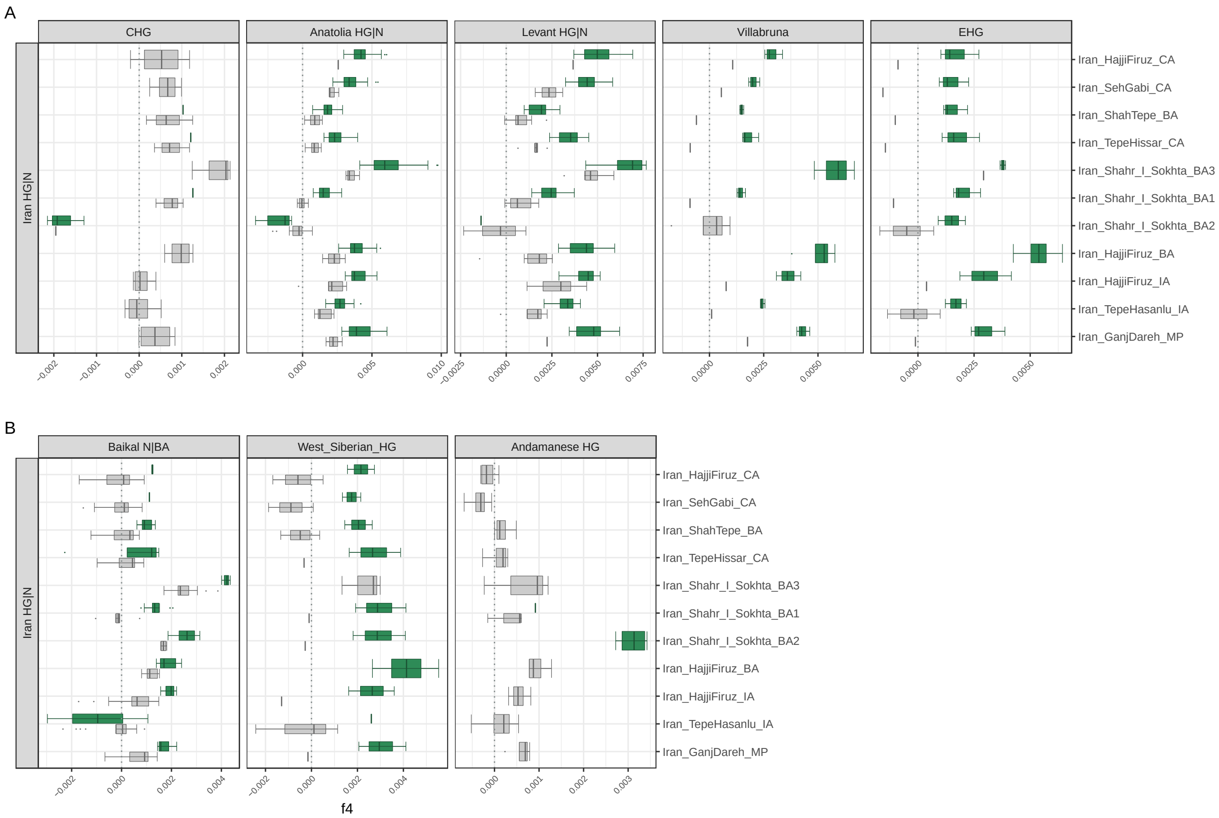

Figure S3: Genetic affinities of post-Neolithic Iran populations, related to Table S3-S4 and Figure 3. Results from the model *f₄(YRI, Test; Iran HG|N, Post Neolithic Iran)*, where *Test* refers to (A) the Anatolian or Levant pre-Neolithic/Neolithic populations, CHG, EHG and Villabruna populations, and (B) Baikal, WSHG and Andamanese HG (Table S3). The Dataset 1 (STAR Methods) was used in the analysis. Boxplots show all f_4 statistics calculated by at least 10K overlapping SNPs. Green colour boxplots show nominally significant f_4-statistics with $|Z| > 3$ and grey colour boxplots show non-significant f_4-statistics with $|Z| < 3$.

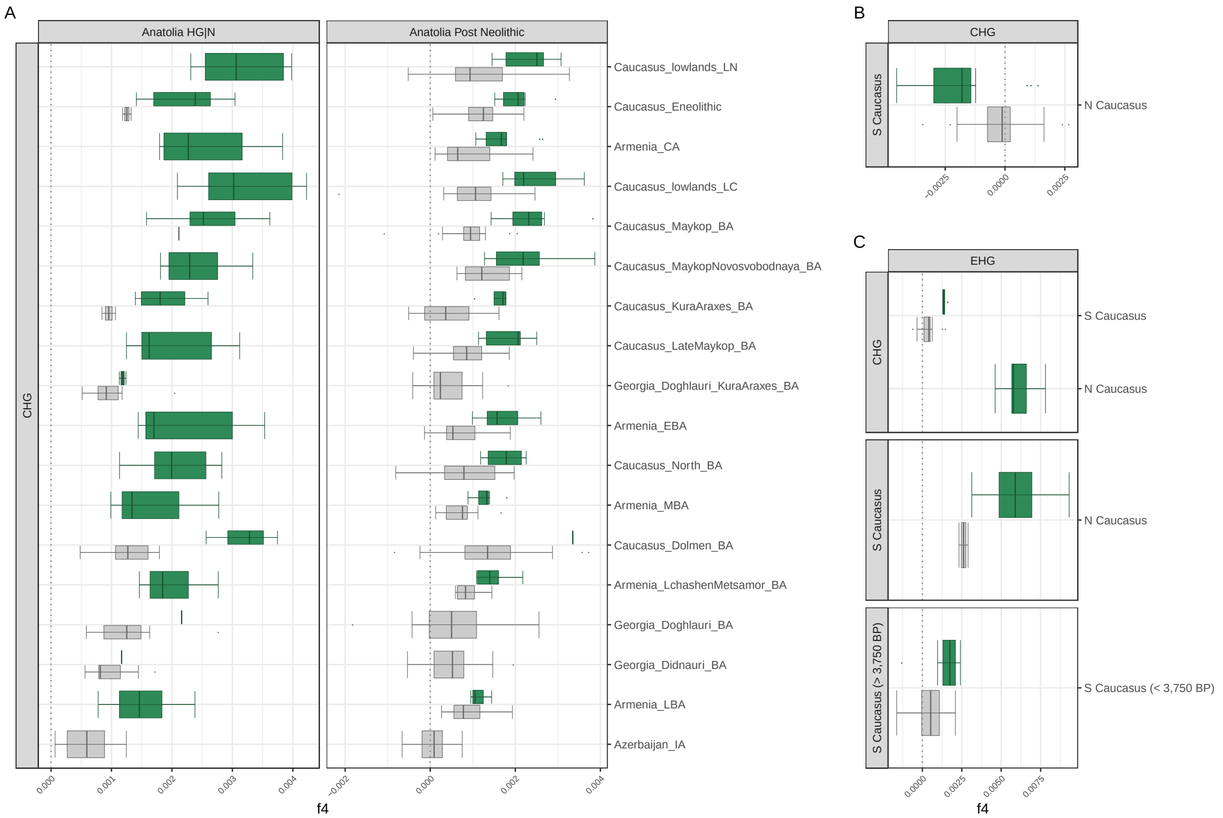

Figure S4: Genetic affinities of post-Neolithic South Caucasus populations, related to Table S3-S4 and Figure 3. Results from (A) f_4*(YRI, Anatolia HG|N or Anatolia Post Neolithic; CHG, South Caucasus Post Neolithic)*, (B) f_4*(YRI, CHG; South Caucasus, North Caucasus)*, and (C) f_4*(YRI, EHG; Pop1, Pop2)*, where *Pop1* or *Pop2* refer to CHG, South Caucasus or North Caucasus populations. The Dataset 1 (STAR Methods) was used in the analysis. Boxplots show all f_4 statistics calculated by using at least 10K overlapping SNPs. Green colour boxplots show nominally significant f_4-statistics with $|Z| > 3$ and grey colour boxplots show non-significant f_4-statistics with $|Z| < 3$.

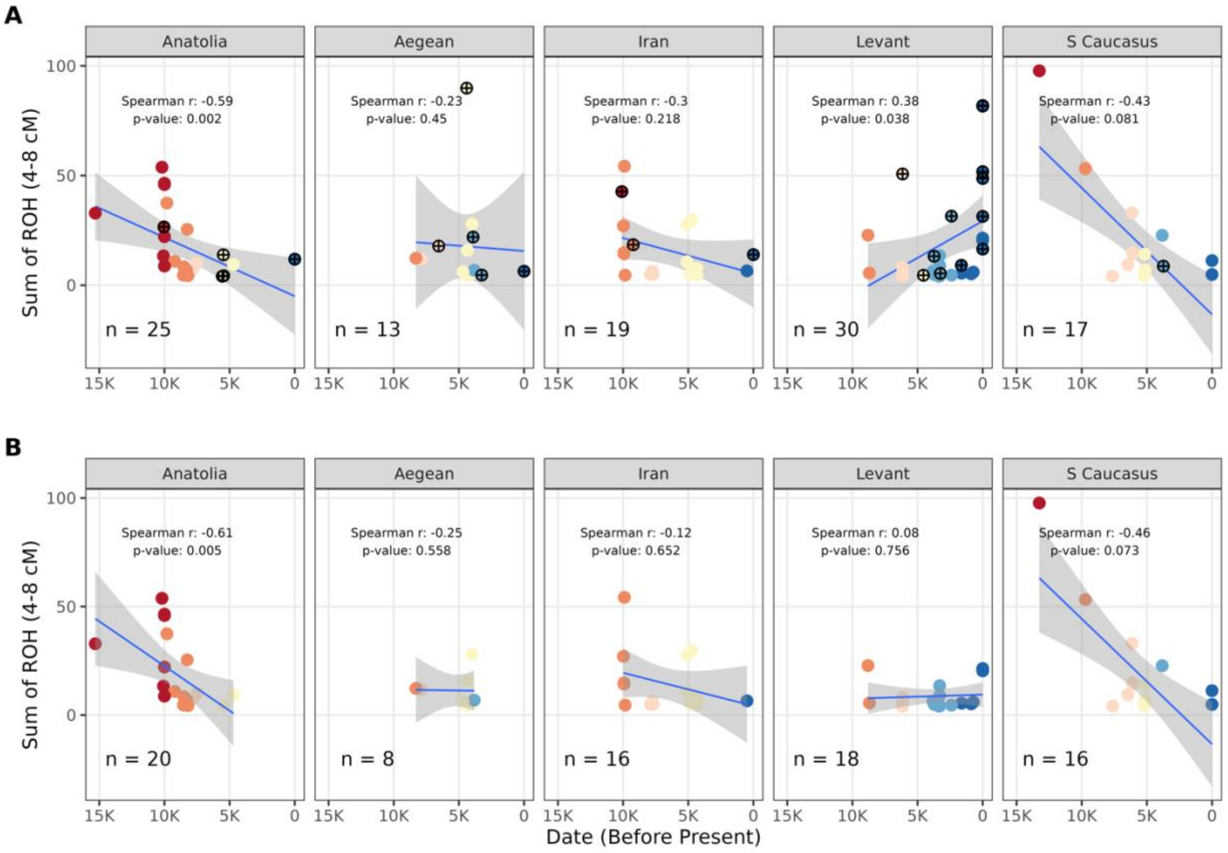

Figure S5: Sum of short (4-8 cM) runs of homozygosity (sROH) among ancient genomes, related to STAR Methods. Panel A shows all individuals for which ROH could be called (with minimum 400,000 SNPs), where individuals with ROH > 20cM, representing consanguinity, are indicated with a plus symbol. Panel B is the same as panel A but after removing likely consanguineous individuals (with ROH > 20cM). The Dataset 2 (STAR Methods) was used in the analysis. The lines in each panel represent linear regression lines. Each point represents an ancient individual and is colour coded based on its time period (Figure 1). We note that we detect no decrease in the sROH values (4-8 cM) for the Aegean, but this is likely because we did not have Aegean genomic samples from the first half of the Holocene with sufficient SNPs to determine ROH. The increase of the sum of ROH in the Levant we observed has been well-documented before and is likely due to an increase in consanguineous marriages in the region in recent times[S1] (see also Table Z11 in Zenodo).

Supplemental References

S1. Scott, E.M., Halees, A., Itan, Y., Spencer, E.G., He, Y., Azab, M.A., Gabriel, S.B., Belkadi, A., Boisson, B., Abel, L., et al. (2016). Characterization of Greater Middle Eastern genetic variation for enhanced disease gene discovery. Nat. Genet. *48*, 1071–1076. 10.1038/ng.3592.

S2. Ceballos, F.C., Gürün, K., Altınışık, N.E., Gemici, H.C., Karamurat, C., Koptekin, D., Vural, K.B., Mapelli, I., Sağlıcan, E., Sürer, E., et al. (2021). Human inbreeding has decreased in time through the Holocene. Curr. Biol. *31*, 3925–3934. 10.1016/j.cub.2021.06.027.

S3. Feldman, M., Gnecchi-Ruscone, G.A., Lamnidis, T.C., and Posth, C. (2021). Where Asia meets Europe – recent insights from ancient human genomics. Ann. Hum. Biol. *48*, 191–202. 10.1080/03014460.2021.1949039.

S4. Harney, É., May, H., Shalem, D., Rohland, N., Mallick, S., Lazaridis, I., Sarig, R., Stewardson, K., Nordenfelt, S., Patterson, N., et al. (2018). Ancient DNA from Chalcolithic Israel reveals the role of population mixture in cultural transformation. Nat. Commun. *9*, 3336. 10.1038/s41467-018-05649-9.

S5. Kılınç, G.M., Omrak, A., Özer, F., Günther, T., Büyükkarakaya, A.M., Bıçakçı, E., Baird, D., Dönertaş, H.M., Ghalichi, A., Yaka, R., et al. (2016). The demographic development of the first farmers in Anatolia. Curr. Biol. *26*, 2659–2666. 10.1016/j.cub.2016.07.057.

S6. Marchi, N., Winkelbach, L., Schulz, I., Brami, M., Hofmanová, Z., Blöcher, J., Reyna-Blanco, C.S., Diekmann, Y., Thiéry, A., Kapopoulou, A., et al. (2022). The genomic origins of the world’s first farmers. Cell *185*, 1842-1859.e18. 10.1016/j.cell.2022.04.008.

S7. Yaka, R., Mapelli, I., Kaptan, D., Doğu, A., Chyleński, M., Erdal, Ö.D., Koptekin, D., Vural, K.B., Bayliss, A., Mazzucato, C., et al. (2021). Variable kinship patterns in Neolithic Anatolia revealed by ancient genomes. Curr. Biol. *31*, 2455–2468. 10.1016/j.cub.2021.03.050.

S8. Skoglund, P., Malmström, H., Omrak, A., Raghavan, M., Valdiosera, C., Günther, T., Hall, P., Tambets, K., Parik, J., Sjögren, K.-G., et al. (2014). Genomic diversity and admixture differs for Stone-Age Scandinavian foragers and farmers. Science *344*, 747–750. 10.1126/science.1253448.

S9. Lazaridis, I., Nadel, D., Rollefson, G., Merrett, D.C., Rohland, N., Mallick, S., Fernandes, D., Novak, M., Gamarra, B., Sirak, K., et al. (2016). Genomic insights into the origin of farming in the ancient Near East. Nature *536*, 419–424. 10.1038/nature19310.

S10. Nielsen, R., and Slatkin, M. (2013). An introduction to population genetics. Sunderland MA Sinauer Assoc. Sch.

S11. Harney, É., Patterson, N., Reich, D., and Wakeley, J. (2021). Assessing the performance of qpAdm: a statistical tool for studying population admixture. Genetics *217*, iyaa045. 10.1093/GENETICS/IYAA045.

S12. de Barros Damgaard, P., Marchi, N., Rasmussen, S., Peyrot, M., Renaud, G., Korneliussen, T., Moreno-Mayar, J.V., Pedersen, M.W., Goldberg, A., Usmanova, E., et al.

(2018). 137 ancient human genomes from across the Eurasian steppes. Nature *557*, 369–374. 10.1038/s41586-018-0094-2.

S13. Clemente, F., Unterländer, M., Dolgova, O., Amorim, C.E.G., Coroado-Santos, F., Neuenschwander, S., Ganiatsou, E., Cruz Dávalos, D.I., Anchieri, L., Michaud, F., et al. (2021). The genomic history of the Aegean palatial civilizations. Cell *184*, 2565–2586. 10.1016/j.cell.2021.03.039.

S14. Feldman, M., Fernández-Domínguez, E., Reynolds, L., Baird, D., Pearson, J., Hershkovitz, I., May, H., Goring-Morris, N., Benz, M., Gresky, J., et al. (2019). Late Pleistocene human genome suggests a local origin for the first farmers of central Anatolia. Nat. Commun. *10*, 1218. 10.1038/s41467-019-09209-7.

S15. Haber, M., Doumet-Serhal, C., Scheib, C., Xue, Y., Danecek, P., Mezzavilla, M., Youhanna, S., Martiniano, R., Prado-Martinez, J., Szpak, M., et al. (2017). Continuity and admixture in the last five millennia of Levantine history from ancient Canaanite and present-day Lebanese genome sequences. Am. J. Hum. Genet. *101*, 274–282. 10.1016/j.ajhg.2017.06.013.

S16. Haber, M., Nassar, J., Almarri, M.A., Saupe, T., Saag, L., Griffith, S.J., Doumet-Serhal, C., Chanteau, J., Saghieh-Beydoun, M., Xue, Y., et al. (2020). A genetic history of the Near East from an aDNA time course sampling eight points in the past 4,000 years. Am. J. Hum. Genet. *107*, 149–157. 10.1016/j.ajhg.2020.05.008.

S17. Kılınç, G.M., Koptekin, D., Atakuman, Ç., Sümer, A.P., Dönertaş, H.M., Yaka, R., Bilgin, C.C., Büyükkarakaya, A.M., Baird, D., Altınışık, E., et al. (2017). Archaeogenomic analysis of the first steps of Neolithization in Anatolia and the Aegean. Proc. R. Soc. B *284*, 20172064. 10.1098/rspb.2017.2064.

S18. Lazaridis, I., Mittnik, A., Patterson, N., Mallick, S., Rohland, N., Pfrengle, S., Furtwängler, A., Peltzer, A., Posth, C., Vasilakis, A., et al. (2017). Genetic origins of the Minoans and Mycenaeans. Nature *548*, 214–218. 10.1038/nature23310.

S19. Narasimhan, V.M., Patterson, N., Moorjani, P., Rohland, N., Bernardos, R., Mallick, S., Lazaridis, I., Nakatsuka, N., Olalde, I., Lipson, M., et al. (2019). The formation of human populations in South and Central Asia. Science *365*, eaat7487. 10.1126/science.aat7487.

S20. Skourtanioti, E., Erdal, Y.S., Frangipane, M., Balossi Restelli, F., Yener, K.A., Pinnock, F., Matthiae, P., Özbal, R., Schoop, U.D., Guliyev, F., et al. (2020). Genomic history of Neolithic to Bronze Age Anatolia, Northern Levant, and Southern Caucasus. Cell *181*, 1158–1175. 10.1016/j.cell.2020.04.044.

S21. Wang, C.-C., Reinhold, S., Kalmykov, A., Wissgott, A., Brandt, G., Jeong, C., Cheronet, O., Ferry, M., Harney, E., Keating, D., et al. (2019). Ancient human genome-wide data from a 3000-year interval in the Caucasus corresponds with eco-geographic regions. Nat. Commun. *10*, 590. 10.1038/s41467-018-08220-8.

S22. Mehrjoo, Z., Fattahi, Z., Beheshtian, M., Mohseni, M., Poustchi, H., Ardalani, F., Jalalvand, K., Arzhangi, S., Mohammadi, Z., Khoshbakht, S., et al. (2019). Distinct genetic variation and heterogeneity of the Iranian population. PLoS Genet. *15*, e1008385. 10.1371/journal.pgen.1008385.

S23. Agranat-Tamir, L., Waldman, S., Martin, M.A.S., Gokhman, D., Mishol, N., Eshel, T., Cheronet, O., Rohland, N., Mallick, S., Adamski, N., et al. (2020). The genomic history of the Bronze Age Southern Levant. Cell *181*, 1146–1157. 10.1016/j.cell.2020.04.024.

S24. Haber, M., Mezzavilla, M., Xue, Y., and Tyler-Smith, C. (2016). Ancient DNA and the rewriting of human history: be sparing with Occam's razor. Genome Biol. *17*, 1. 10.1186/s13059-015-0866-z.

S25. Feldman, M., Master, D.M., Bianco, R.A., Burri, M., Stockhammer, P.W., Mittnik, A., Aja, A.J., Jeong, C., and Krause, J. (2019). Ancient DNA sheds light on the genetic origins of early Iron Age Philistines. Sci. Adv. *5*, eaax0061. 10.1126/sciadv.aax0061.

S26. Altınışık N.E., Kazancı D.D., Aydoğan A., Gemici H.C., Erdal Ö.D., Sarıaltun S., et al. A genomic snapshot of demographic and cultural dynamism in Upper Mesopotamia during the Neolithic Transition. Sci. Adv. 2022;8:eabo3609. doi:10.1126/sciadv.abo3609
