# Supplementary material for: Spatial and temporal heterogeneity in human mobility patterns in Holocene Southwest Asia and the East Mediterranean
Source: Curr Biol. 2023 Jan 9;33(1):41–57.e15. doi: 10.1016/j.cub.2022.11.034 (PMC9839366; doi:10.1016/j.cub.2022.11.034)
Supplement: Methods S1. Supplemental discussion on estimation of human mobility and changing genetic diversity over time, related to Figures 1, 3, and 5 and STAR Methods — (D) Comparison of changes in ancestry components and inter-regional genetic distances over time. [file mmc6.pdf]

Methods S1: Supplemental discussion on estimation of human mobility and changing genetic diversity over time, related to Figure 1, 3, 5, Table 1, Table S1-S2 and STAR Methods.

A. Discussion on previous observations about human mobility patterns in Holocene Southwest Asia and the East Mediterranean

Recent archaeogenomic studies have revealed a number of interesting observations on the gene pools in Southwest Asia and the East Mediterranean, which we summarise and discuss below. We also discuss why certain population genetic observations (e.g. decreasing F_{ST} over time) can be interpreted as indicating inter-regional admixture and mobility. Finally, we discuss how we may reconcile historical observations on increasing mobility with previously observed population genetics patterns that hint at reduced mobility.

a) Within-population genetic diversity: Diversity levels were low in the early Holocene, but appear to have increased following the Neolithic transition[S2-S7]. This is compatible with increasing admixture within the studied populations. Meanwhile, local population growth without admixture would not explain this observation. This is because diversity in at least some of these studies[S5,S8] was measured using SNPs ascertained in populations from Africa, representing outgroups to present-day Eurasians. These variants, therefore, arose earlier than the separation among the Eurasian populations under study. Any de novo variants that could accumulate in growing populations and that would normally increase diversity would be invisible to such analysis. Likewise, our observation of increasing diversity in Southwest Asia cannot be explained by de novo variants. This leaves mobility behind.

b) Inter-population genetic differentiation: F_{ST} is a commonly used measure of population differentiation, roughly comparing population divergence with total diversity (including within-population diversity). F_{ST} values calculated among West Eurasian human groups before the Neolithic were high, but dropped sharply during the Neolithic and Chalcolithic periods[S3,S4,S9]. Low genetic diversity and high F_{ST} observed by the late Pleistocene and early Holocene suggest population isolation in earlier periods. During the Neolithic period, especially with the westward and eastward Neolithic expansions around 9,000 Before Present (BP), there appears to have occurred rapid admixture and consequent genetic homogenization across the region. Importantly, inter-population differentiation (average F_{ST}) appears to have strongly decreased from early-to-mid-Holocene[S3,S4,S9]. A drop in F_{ST} between two groups is compatible with inter-population admixture, which would cause shorter coalescence times[S10].

c) Ancestry components: Ancestry components can be inferred using qpAdm analysis[S11], where a genome is explained as a mixture of source genomes. Such analysis can reveal potential admixture events if the sources include earlier populations from the same region, and under the assumption of limited population structure within a region. If this is the case, any change in ancestry components over time should be caused by admixture.

In Southwest Asia, qpAdm analyses on genomes from the Holocene have yielded a number of important insights. The foremost for our study is widespread inter-regional admixture from the Neolithic to the Bronze Ages, especially between eastern (Iran and South Caucasus) and western (Anatolia and Levant) Southwest Asia, and also extending into the Aegean[S4,S7,S9,S12–S21]. For instance, by the Early Bronze Age, Anatolian groups carried approximately 50% ancestry of

eastern origin (related to early Holocene South Caucasus/Iran), while Zagros populations carried approximately 50% ancestry of western origin (related to early Holocene Anatolia)[S19,S20].

During the Bronze Age, Steppe- or West Siberia-related gene flow is also observed in the broad region, albeit at lower levels and not ubiquitously[S9,S12,S13,S16,S19-S21]. Importantly, changes in admixture components appear to be more modest in the period between the Bronze Age period and the present-day. Analysing past and extant populations of present-day Iran[S22] of the Levant[S15,S16,S23], of the Caucasus[S21,S24], and of present-day Greece have suggested limited or even no observable change in ancestry components over the last 3,000-4,000 years[S13,S18].

While singular ancient genomes with non-local ancestry (often dubbed "outliers") are occasionally discovered, these mobility events appear to not have left substantial traces in the local gene pools from the Bronze Age onwards[S16,S19,S20,S25].

We next discuss the signal of decreasing F_{ST} (differentiation) and increasing stability (lower change) in ancestry components in the latter half of the Holocene. On the surface, these observations may seem to imply a decrease in mobility with the Bronze Ages. But this would be at odds with rich archaeological, historical and linguistic evidence for inter-regional human movements in the same period, from the establishment of trade colonies to forced population transfers (see Table I in Document Z1). A number of non-exclusive explanations could be conceived to reconcile expected inter-regional mobility and the observed genetic stability pattern:

1) Even if migration continued, the large size of resident agricultural populations that emerged by the Bronze Age may have diluted the genetic impact of immigrants[S3,S16].

2) Low amounts of gene flow simultaneously emerging from diverse external sources may not be visible in qpAdm analyses and may not cause an increase in F_{ST}, thus producing the wrong impression of reduced mobility.

3) The relative homogenization among regional gene pools in Southwest Asia in the early Holocene may have rendered later mobility events within Southwest Asia less visible to qpAdm analyses. Specifically, after an initial bout of admixture e.g. during the Neolithic, further admixture between the same groups e.g. during the Bronze Age may be difficult to detect via qpAdm.

4) The post-Bronze Age samples from the region may not be sufficiently representative of demographic changes in this period [for instance, an analysis of a relatively rich temporal sample from the Levant[S15] reported subtle admixture from the Iron Age to the Ottoman Period from external regions (the Steppe, Africa, South Asia, and Central Asia) not observed earlier]. Nevertheless, the largely consistent patterns of ancestry change observed using genomes from different sites from each region, including the new genomes presented here (Figure 3), overall suggest that insufficient sampling is not a major issue.

5) F_{ST} may not be the optimal statistic to gauge rates of inter-population migration, as it is influenced by within-population diversity. For instance, F_{ST} is sensitive to population size reduction, which can cause an increase in F_{ST} without admixture (see Figure VII in Document Z1).

B. Discussion on interpretation of ancestry proportion estimations

In our qpAdm analyses we specifically model the population of each period with the earlier period as sources from the region. More precisely, we attempt to explain regional populations A or B at time T+1 ("target"), as mixtures of populations of the same regions A and B at time T ("sources").

This analysis revealed that, in a number of instances, we need additional ancestry sources to explain the later-coming genome sample (e.g. the post-Neolithic Zagros sample needing Anatolian ancestry in addition to the earlier Zagros Neolithic ancestry). This would be compatible with inter-regional mobility. Still, there remain some significant limitations in interpreting qpAdm results:

- The appearance of an ancestry component related to population B at time T in population A at time T+1 does not mean a movement from B to A. To avoid such overinterpretation, we use "population X-related" to describe putative admixture events.
- There is the possibility of a hidden and strong population structure that confounds temporal change. If intra-regional genetic differences (differences within region A) are larger than inter-regional differences (between A and B), this would render the inference of mobility invalid. But if we can assume that strong population structure is not common (which is usually, though not always, the case at the time scales we use; see Figure 1 and 3), changing ancestry sources in qpAdm could be interpreted as mobility.
- High genetic similarity between the target and source populations can introduce high noise in qpAdm analysis. For instance, in our analyses, we found a wide range of possible Anatolian contributions (0-42%) in Levant genomes (Table S4). This is likely caused by high affinity between Anatolia and Levant populations in the early Holocene. The pre-Neolithic Anatolian gene pool itself is thought to have arisen from admixture between Levant and European pre-Neolithic groups, and the Neolithic Anatolians are thought to have descended from this gene pool[S25,S26]. As a consequence, when the source populations share high drift (common ancestry), qpAdm modelling has difficulty in precisely estimating the proportion of admixture.
- qpAdm-estimated proportions are sometimes treated as migration rate estimates, but they cannot be interpreted as such unless the direct sources are known.
- Multiple models may explain the same data and may be similarly valid. In Figure 3 we chose to represent only one, following the criteria explained in the STAR Methods. Meanwhile, using alternative models (e.g. with similar but slightly different sources, such as CHG vs. Iran Neolithic) would yield the same qualitative conclusions about putative admixture-events.

C. Discussion on the possible effects of heterogeneous datasets on the results

The fact that we are using a heterogeneous dataset comprises a wide spectrum of whole genome coverage values (from 0.1X to 11.5X for shotgun samples and from 0.0007X to 0.50X for 1240K capture) and different protocols (shotgun sequencing versus SNP capture) requires additional consideration:

<u>1) Low depth of coverage:</u> This can lead to false negative results (missing subtle admixture signals) and it may introduce noise in f_3 statistics.

Meanwhile, low coverage is not expected to cause a bias in f_3 statistics (i.e. systematically higher affinity towards one population over another). Consequently, our main observations involving temporal changes (increasing diversity over time, the pattern of reduced and later increased inter-regional differences, and the sex-bias effect) are not expected to be influenced by heterogeneity in the coverage, as we explain below:

1a) We find that when we use all the data (ancient shotgun and SNP capture, and present-day genomes), there is a correlation between time and coverage (Spearman's rho = -0.16, p=0.0005). This appears to be driven by modern-day genomes (~30X coverage per genome), and also by having genomes produced by using two different protocols within the dataset (shotgun sequencing versus SNP capture). However, within the two ancient genome datasets produced by these protocols, we do not find a correlation between time and coverage (Spearman's rho = -0.05, p=0.34 in the 1240K data; Spearman's rho = 0.06, p=0.47 in the shotgun data). This is important because when we analyse the two (shotgun and capture) datasets separately, we find the same results again (Figure XIX and Figure XX in Document Z1).

1b) The outgroup-f_3 comparisons can be affected by the number of SNPs used in the calculation (i.e. very low SNPs may introduce strong noise). In our original analyses we had used > 2,000 SNPs as cutoff for calculations for autosomal SNPs and > 1,000 SNPs for X chromosome SNPs (see STAR Methods). When we repeat our main observations involving temporal changes (increasing diversity over time, the pattern of reduced and later increased inter-regional differences, and the sex-bias effect) by using 10K (Figure X in Document Z1) and 50K SNPs (Figure XI in Document Z1) cutoff calculations for autosomal SNPs, we observe the same patterns.

2) Combining data produced by different laboratories runs the risk of technical effects (related to laboratory protocols or data processing) confounding biological signals. To minimize this risk we took two measures:

- We processed all ancient genome BAM files from external sources through the same pipeline as the newly generated data (STAR Methods).
- For our main novel observations, we repeated the analyses separately using shotgun sequencing and SNP capture-based datasets (Figures XV, XVI in Document Z1).

D. Comparison of changes in ancestry components and inter-regional genetic distances over time

Here we describe changes in ancestry components estimated using qpAdm (Figure 3) and corresponding changes in inter-regional genetic distances (Figure 5C) in three examples:

Anatolia-Caucasus: In TP1 and TP2, the profile of South Caucasus is represented by the CHG sample (the pink component in Figure 3), distinct from that in Anatolia[S5]. In TP3 in the Caucasus (the first two Caucasus genomes Figure 3), we find a >50% Anatolian-related component represented by Barcin_CA. In Anatolia, TP3 is represented by Buyukkaya_EC and Kumtepe_CA, both of which can be modeled as earlier populations (Barcin_N) and GanjDareh_N, the latter similar to CHG[S9]. These imply bidirectional gene flow between the two regions. We also observe a sharp reduction in genetic distance between the two regions in TP3. However, genetic divergence rebounds in TP5 and TP6, which corresponds to the introduction of EHG-related ancestry in the Caucasus in TP5, and also Siberian-related ancestry in Anatolia in TP6.

Iran-Levant: Between these two regions, we find a sharp reduction in genetic distances in TP3. In the same period, we observe both Iran_BA-related and Anatolia_EN-related components appearing in the Levant, and a large Anatolian-related component (represented by Buyukkaya_EC) appearing in Iran. Importantly, this Anatolian-related component itself contains Levant-related ancestry (seen in the Barcin_N genome in Figure 3, and also present in the Anatolian_EN ancestry; see Feldman et al.[S25]). Genetic distances tend to rise in subsequent periods, which includes EHG- and Siberia-related ancestry appearing in Iran, and an Anatolia_EN-related component appearing in the Levant in TP6 (the latter may represent Europe-related gene flow).

Anatolia-Aegean: The initial genetic profiles of these two regions are highly similar, with the same qpAdm components except for CHG and GanjDareh_N (which are highly similar to each other[S9]) being used as the eastern source. They diverge from TP4 onward in their (1- f_3) distances, which corresponds to the introduction of EHG-related ancestry in the Aegean, and also Siberian-related ancestry in Anatolia.
