# Supplementary material for: Spatial and temporal heterogeneity in human mobility patterns in Holocene Southwest Asia and the East Mediterranean
Source: Curr Biol. 2023 Jan 9;33(1):41–57.e15. doi: 10.1016/j.cub.2022.11.034 (PMC9839366; doi:10.1016/j.cub.2022.11.034)
Supplement: Document S2. Article plus supplemental information [file mmc7.pdf]

Current Biology

Article

Spatial and temporal heterogeneity in human mobility patterns in Holocene Southwest Asia and the East Mediterranean

Highlights

- Genetic diversity in Southwest Asia increased continuously through the Holocene
- Regional populations admixed among themselves with the Neolithic
- After the Bronze Age, populations diverged from each other via distant gene flow
- Male-to-female bias increased over time in inter-regional human movements

Authors

Dilek Koptekin, Eren Yüncü, Ricardo Rodríguez-Varela, ..., Füsun Özer, Anders Götherström, Mehmet Somel

Correspondence

dilek.koptekin@metu.edu.tr (D.K.),
anders.gotherstrom@arklab.su.se (A.G.),
msomel@metu.edu.tr (M.S.)

In brief

Koptekin et al. use ancient genomes to infer population movements in Southwest Asia through 10,000 years, which saw the emergence of agriculture and later of complex societies with distant connections. The authors propose "the expanding mobility model," where migration ranges increased over time, accompanied by growing male bias in movements.

Koptekin et al., 2023, Current Biology *33*, 41–57
January 9, 2023 © 2022 The Authors. Published by Elsevier Inc.
https://doi.org/10.1016/j.cub.2022.11.034

CellPress

Current Biology

CellPress
OPEN ACCESS

Article

Spatial and temporal heterogeneity in human mobility patterns in Holocene Southwest Asia and the East Mediterranean

Dilek Koptekin,[1,2,45,*] Eren Yüncü,[2] Ricardo Rodríguez-Varela,[3,4,42] N. Ezgi Altınışık,[5,42] Nikolaos Psonis,[6,42] Natalia Kashuba,[7] Sevgi Yorulmaz,[2] Robert George,[3,8] Duygu Deniz Kazancı,[2,5] Damla Kaptan,[2] Kanat Gürün,[2] Kıvılcım Başak Vural,[2] Hasan Can Gemici,[9] Despoina Vassou,[6] Evangelia Daskalaki,[4] Cansu Karamurat,[9] Vendela K. Lagerholm,[3,4] Ömür Dilek Erdal,[10] Emrah Kırdök,[11] Aurelio Marangoni,[3] Andreas Schachner,[12] Handan Üstündağ,[13] Ramaz Shengelia,[14] Liana Bitadze,[15] Mikheil Elashvili,[16] Eleni Stravopodi,[17]

(Author list continued on next page)

[1]Department of Health Informatics, Graduate School of Informatics, Middle East Technical University, 06800 Ankara, Turkey
[2]Department of Biological Sciences, Middle East Technical University, 06800 Ankara, Turkey
[3]Centre for Palaeogenetics, Stockholm, Sweden
[4]Department of Archaeology and Classical Studies, Stockholm University, 10691 Stockholm, Sweden
[5]Human-G Laboratory, Department of Anthropology, Hacettepe University, Beytepe 06800, Ankara, Turkey
[6]Ancient DNA Lab, Institute of Molecular Biology and Biotechnology (IMBB), Foundation for Research and Technology – Hellas (FORTH), N. Plastira 100, Vassilika Vouton, GR-70013 Irakleio, Greece
[7]Department of Archaeology and Ancient History, Archaeology, Uppsala University, Uppsala, Sweden
[8]School of Medicine, University of Notre Dame, Sydney, Australia
[9]Department of Settlement Archaeology, Middle East Technical University, 06800 Ankara, Turkey

(Affiliations continued on next page)

SUMMARY

We present a spatiotemporal picture of human genetic diversity in Anatolia, Iran, Levant, South Caucasus, and the Aegean, a broad region that experienced the earliest Neolithic transition and the emergence of complex hierarchical societies. Combining 35 new ancient shotgun genomes with 382 ancient and 23 present-day published genomes, we found that genetic diversity within each region steadily increased through the Holocene. We further observed that the inferred sources of gene flow shifted in time. In the first half of the Holocene, Southwest Asian and the East Mediterranean populations homogenized among themselves. Starting with the Bronze Age, however, regional populations diverged from each other, most likely driven by gene flow from external sources, which we term "the expanding mobility model." Interestingly, this increase in inter-regional divergence can be captured by outgroup-f_3-based genetic distances, but not by the commonly used F_{ST} statistic, due to the sensitivity of F_{ST}, but not outgroup-f_3, to within-population diversity. Finally, we report a temporal trend of increasing male bias in admixture events through the Holocene.

INTRODUCTION

Human mobility can be a driver of sociocultural change, but also an outcome. Studying spatiotemporal patterns of mobility together with sociocultural transitions is of critical importance to understanding the human past. Southwest Asia and the East Mediterranean present an attractive case here, with their exceptionally long history of food-producing societies. The region was center stage of key cultural and social transformations during the Holocene, from the earliest sedentary villages and agriculture to the earliest metallurgy, the emergence of state-organized societies, the first writing systems, and more recently, inter-regional empires (Table I in Document Z1). This period also witnessed changes that directly affected human mobility dynamics, such as population growth, the establishment of long-distance trade networks supported by transport animals and road construction, the organization of invading armies, and mass deportations.[1–5]

Recently, archaeogenomic studies have revealed interesting observations relevant to inter-regional mobility in Southwest Asia and the East Mediterranean. One such finding is that within-population genetic diversity levels were low in the early Holocene, but increased following the Neolithic transition.[6–10] A parallel observation is that inter-population genetic differentiation, as measured by F_{ST}, was high among West Eurasian human groups before the Neolithic, but dropped sharply during the Neolithic and Chalcolithic periods.[7,8,11] Interpreting a reduction in F_{ST} between regions is not straightforward as it can be caused by multiple demographic processes (Methods S1A), but a likely cause is admixture, suggesting widespread inter-regional movement and gene flow during the Neolithic.

This is an open access article under the CC BY license (http://creativecommons.org/licenses/by/4.0/).

Mihriban Özbaşaran,[18] Güneş Duru,[19] Argyro Nafplioti,[6] C. Brian Rose,[20] Tuğba Gencer,[21] Gareth Darbyshire,[22] Alexander Gavashelishvili,[23] Konstantine Pitskhelauri,[24] Özlem Çevik,[25] Osman Vuruşkan,[25] Nina Kyparissi-Apostolika,[17] Ali Metin Büyükkarakaya,[26,41] Umay Oğuzhanoğlu,[27] Sevinç Günel,[28] Eugenia Tabakaki,[6] Akper Aliev,[29] Anar Ibrahimov,[30] Vaqif Shadlinski,[31] Adamantios Sampson,[32] Gülşah Merve Kılınç,[33] Çiğdem Atakuman,[34] Alexandros Stamatakis,[35,36] Nikos Poulakakis,[6,37,38] Yılmaz Selim Erdal,[5,10] Pavlos Pavlidis,[39] Jan Storå,[40] Füsun Özer,[5,43] Anders Götherström,[3,4,43,*] and Mehmet Somel[1,2,43,44,*]
[10]Husbio-L Laboratory, Department of Anthropology, Hacettepe University, 06800 Beytepe, Ankara, Turkey
[11]Department of Biotechnology, Mersin University, 33343 Yenişehir, Mersin, Turkey
[12]Deutsches Archäologisches Institut, Inönü Cad. 10, Gümüşsuyu, 34437 İstanbul, Turkey
[13]Department of Archaeology, Anadolu University, 26470 Eskişehir, Turkey
[14]Department of the History of Medicine and Bioethics, Tbilisi State Medical University, Tbilisi 0162, Georgia
[15]Institute of History and Ethnology, Tbilisi State University, Tbilisi, Georgia
[16]Cultural Heritage and Environment Research Center, School of Natural Sciences and Medicine, Ilia State University, Tbilisi, Georgia
[17]Ephorate of Palaeoanthropology and Speleology, Ministry of Culture and Sports, 11636 Athens, Greece
[18]Department of Prehistory, Istanbul University, 34134 Istanbul, Turkey
[19]Mimar Sinan Fine Arts University, 34134 Istanbul, Turkey
[20]Department of Classical Studies, University of Pennsylvania, Philadelphia, PA, USA
[21]Department of History of Medicine and Ethics, Cerrahpasa Faculty of Medicine, Istanbul University, Istanbul, Turkey
[22]Penn Museum, Philadelphia, PA, USA
[23]Center of Biodiversity Studies, Institute of Ecology, Ilia State University, Cholokashvili Str. 5, Tbilisi 0162, Georgia
[24]Ilia State University, Cholokashvili Str. 5, Tbilisi 0162, Georgia
[25]Department of Archaeology, Trakya University, Edirne, Turkey
[26]Department of Anthropology, Hacettepe University, 06800 Beytepe, Ankara, Turkey
[27]Department of Archaeology, Pamukkale University, Denizli, Turkey
[28]Department of Archaeology, Hacettepe University, 06800 Beytepe, Ankara, Turkey
[29]Azerbaijan DNA Project, Family Tree DNA, Houston, TX, USA
[30]Khazar University, Baku, Azerbaijan
[31]Azerbaijan Medical University, Baku, Azerbaijan
[32]Department of Mediterranean Studies, University of Aegean, Dimokratias st., 85100 Rhodes, Greece
[33]Department of Bioinformatics, Graduate School of Health Sciences, Hacettepe University, 06100 Ankara, Turkey
[34]Institute of Social Sciences, Middle East Technical University, 06800 Ankara, Turkey
[35]Computational Molecular Evolution Group, Heidelberg Institute for Theoretical Studies, 69118 Heidelberg, Germany
[36]Institute for Theoretical Informatics, Karlsruhe Institute of Technology, 76131 Karlsruhe, Germany
[37]Natural History Museum of Crete, School of Sciences and Engineering, University of Crete, Knossos Avenue, 71409 Irakleio, Greece
[38]Department of Biology, School of Sciences and Engineering, University of Crete, Vassilika Vouton, 70013 Irakleio, Greece
[39]Institute of Computer Science, Foundation for Research and Technology-Hellas (FORTH), 70013 Heraklion, Greece
[40]Osteoarchaeological Research Laboratory, Department of Archaeology and Classical Studies, Stockholm University, 10691 Stockholm, Sweden
[41]Human Behavioral Ecology and Archaeometry Laboratory (IDEA Lab), Hacettepe University, Ankara, Turkey
[42]These authors contributed equally
[43]These authors contributed equally
[44]Twitter: @CompEvoMetu
[45]Lead contact
*Correspondence: dilek.koptekin@metu.edu.tr (D.K.), anders.gotherstrom@arklab.su.se (A.G.), msomel@metu.edu.tr (M.S.)
https://doi.org/10.1016/j.cub.2022.11.034

Results from ancestry component analyses similarly imply extensive inter-regional admixture from the Neolithic to the Bronze Age (BA), especially between eastern (Iran and South Caucasus) and western (Anatolia and Levant) Southwest Asia, extending into the Aegean.[8,11–22] Intriguingly, however, changes in admixture components appear more modest in the period between the BA and the present-day. Studies on past and extant populations of present-day Iran,[23] of the Levant,[15,16,24] of the Caucasus,[21,25] and of present-day Greece have suggested limited or even no observable change in ancestry components over the last 3,000–4,000 years.[13,18] Although singular ancient genomes with non-local ancestry are occasionally discovered, these mobility events appear not to have left substantial traces in local gene pools from the BA onward.[16,19,20,26] This may appear surprising because both historical and archaeological sources indicate widening mobility networks after the Chalcolithic and the BA periods connecting Southwest Asia with wider regions, based on which one might anticipate accelerating admixture and genetic change (Table I in Document Z1; Methods S1A).

The dynamics of inter-regional human mobility in Southwest Asia and the East Mediterranean during the Holocene thus remain unsettled. Here, we systematically study this problem using 35 newly produced ancient genomes, together with published and modern-day genomes. We describe the overall genetic structure of the region, explore temporal shifts in within-population diversity and inter-regional divergence, and analyze inter-regional differences in mobility rates. Finally, we tackle the question of possible sex bias in human mobility, given earlier suggestions of long-term matrilineal continuity in the region.[27–29]

CellPress
OPEN ACCESS

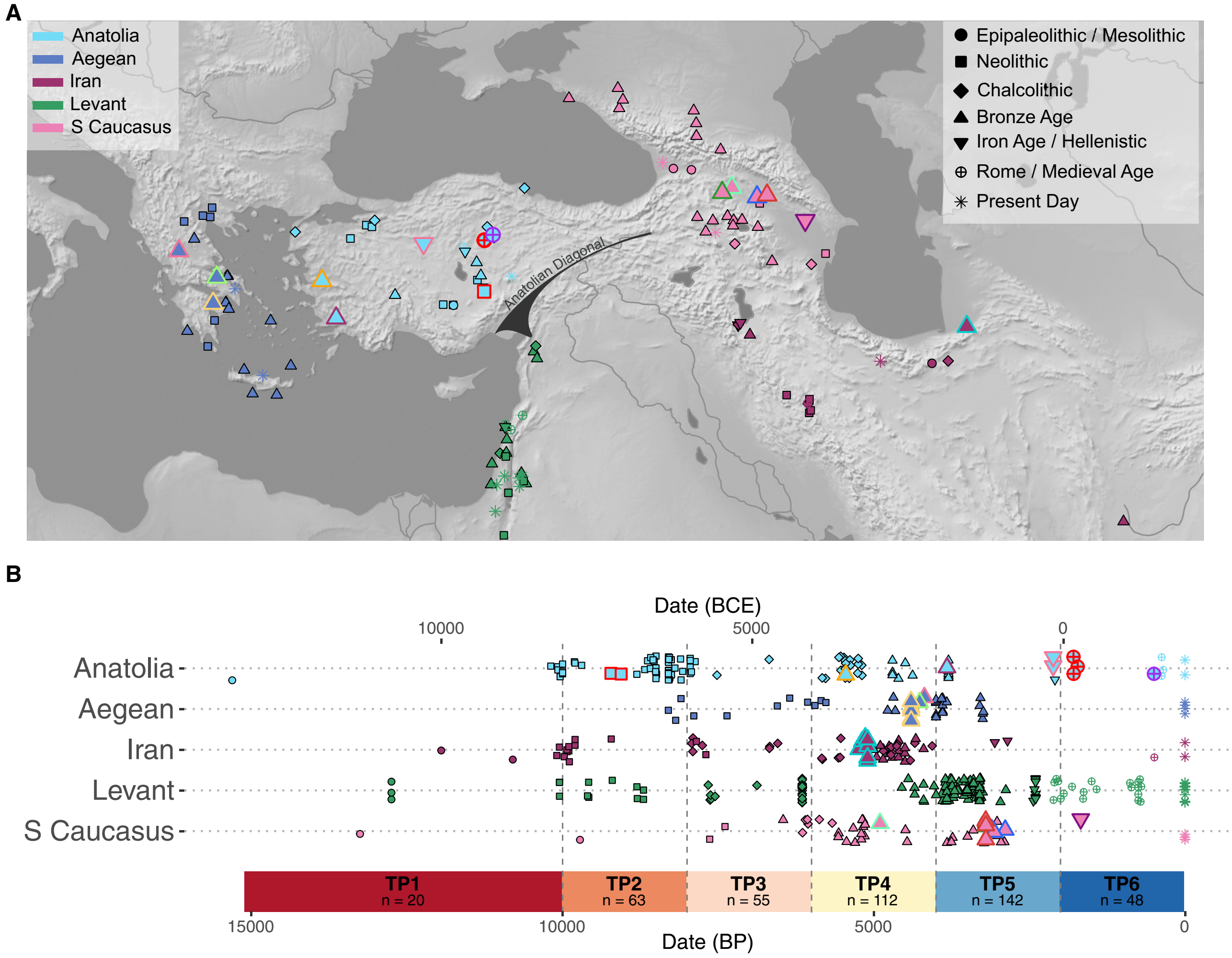

Figure 1. Geographical location of archaeological sites and dates

(A) The locations and (B) dates of ancient individuals analyzed in this study. TP denotes "time period," and the number of samples for each TP is shown (see also Table II in Document Z1). The colors and symbols for ancient samples are the same as in the principal component analysis (PCA) in Figure 2. Symbols indicate the archaeological/historical period associated with the individuals. Larger symbols with colored outlines represent the new ancient genomes presented in this study. Present-day samples are shown with an asterisk. If samples were not directly radiocarbon dated, we used approximate dates based on the archaeological context. "Date (BP)" values were calculated by adding 1,950 years to the average of the calibrated/context-based date intervals. The approximate location of the Anatolian Diagonal is shown on the map. To improve visualization, we used the "geom_jitter" function implemented in "ggplot"; therefore, the locations shown may slightly deviate from the exact coordinates, which are given in Table S2.
See Tables S1 and S2, Methods S1, and STAR Methods for details.

RESULTS

Our study focuses on human population dynamics on five geographically and culturally connected regions (Figure 1): (1) Anatolia, which we describe as the peninsula to the west of the Anatolian diagonal (the mountain range extending between the North Levant and the eastern Black Sea coast of present-day Turkey); (2) the Aegean, including present-day mainland Greece, the Cyclades, and Crete; (3) present-day Iran, including the Zagros area and South Caspian; (4) South Caucasus, comprising present-day Georgia, Southwest Russia, Armenia, and Azerbaijan; and (5) the Levant, comprising present-day West Syria, Lebanon, Palestine, Israel, and Jordan. These regions contain the highest intensity of published ancient genomes in Southwest Asia and the East Mediterranean (for which reason we did not include Mesopotamia or the Arabian Peninsula).

Genetic structure and continuity in southwest asia and the east mediterranean

From these five regions, we produced 35 new ancient shotgun-sequenced genomes, with coverages ranging between 0.02× and 7.5× (mean = 1.11×, median = 0.33×) per genome, and radiocarbon dated 15 of these individuals (Tables 1 and S1; Figure I in Document Z1). We then combined the new data with published ancient and present-day genomes from the same regions (Figure 1; Table S2; STAR Methods). The new genomes extend the geographic and temporal coverage of the published samples, e.g., by including the Iron Age (IA) in South

Table 1. Archaeological and genetic information of the ancient individuals sequenced in this study

Region	Sample ID	Location	Date (BCE/CE)	Average date (BP)	Genome coverage	Sex	mtDNA haplogroup	Y chr haplogroup
Anatolia	BOG019	Boğazköy, Turkey	100–350 CE	1,725	0.326	XY	X2n	T1a1
Anatolia	BOG020	Boğazköy, Turkey	130–190 CE	1,790	2.202	XY	X2f	J2a1h
Anatolia	BOG024	Boğazköy, Turkey	130–190 CE	1,790	0.484	XY	H13c1a	J
Anatolia	BOG028	Boğazköy, Turkey	1,000–1,900 CE	500	1.332	XX	HV1b3b	–
Anatolia	CTG025	Çine-Tepecik, Turkey	1,977–1,772 calBCE	3,825	0.191	XX	W6b	–
Anatolia	GOR001	Gordion, Turkey	333–0 BCE	2,116	7.548	XY	H14a	J2a1
Anatolia	GOR002	Gordion, Turkey	333–0 BCE	2,116	0.074	XX	K1a3	–
Anatolia	mus005	Musular, Turkey	7,377–7,167 calBCE	9,222	2.463	XX	K1a4	–
Anatolia	mus006	Musular, Turkey	7,180–7,039 calBCE	9,060	0.140	XY	N1a1a1b	CT
Anatolia	ulu117	Ulucak, Turkey	4,000–3,000 BCE	5,450	0.360	XX	J1c11	–
Aegean	G23	Theopetra, Greece	2,335–2,140 calBCE	4,188	0.426	XY	H5	I
Aegean	G37	Sarakinos, Greece	2,325–2,300 calBCE	4,263	0.228	XY	H11a2	J
Aegean	G31	Perachora, Greece	2,700–2,200 BCE	4,350	0.213	XY	J1c2	BT
Aegean	G62	Perachora, Greece	2,700–2,200 BCE	4,350	0.628	XY	J1c	G2a2b2a
Aegean	G65	Perachora, Greece	2,700–2,200 BCE	4,350	0.271	XX	T2c1d+152	–
Aegean	G66	Perachora, Greece	2,700–2,200 BCE	4,350	0.112	XX	H2a	–
Aegean	G76a	Perachora, Greece	2,569–2,340 calBCE	4,405	0.739	XX	T2c1+146	–
S Caucasus	geo005	Didnauri, Georgia	1,258–1,049 calBCE	3,104	0.077	XY	U7b	R1b1a2a2
S Caucasus	geo006	Didnauri, Georgia	1,041–837 calBCE	2,889	0.046	XY	X2	O1b1a2
S Caucasus	geo015	Doghlauri, Georgia	3,016–2,886 calBCE	4,901	0.189	XY	K1a	J2a1b1
S Caucasus	geo017	Doghlauri, Georgia	1,373–1,118 calBCE	3,196	0.033	XX	H4b	–
S Caucasus	geo029	Didnauri, Georgia	1,220–1,016 calBCE	3,068	0.092	XY	I5c	R1b1a2a2
S Caucasus	gur016	Nazarlebi, Georgia	1,500–1,000 BCE	3,250	0.021	XY	K	A
S Caucasus	gur017	Nazarlebi, Georgia	1,500–1,000 BCE	3,250	0.215	XY	N1a1a1a	BT
S Caucasus	gur019	Nazarlebi, Georgia	1,500–1,000 BCE	3,250	0.030	XX	K1a4b	–
S Caucasus	zrj003	Shamakhi, Azerbaijan	206–347 calCE	1,674	0.273	XY	K1a19	J
Iran	sha003	Shahtepe, Iran	3,200–3,100 BCE	5,100	3.346	XX	H14	–
Iran	sha004	Shahtepe, Iran	3,487–3,101 calBCE	5,244	3.877	XY	I1a	J
Iran	sha006	Shahtepe, Iran	3,200–3,100 BCE	5,100	2.548	XX	J1b1b1	–
Iran	sha007	Shahtepe, Iran	3,368–3,100 calBCE	5,184	3.945	XX	HV13b	–
Iran	sha008	Shahtepe, Iran	3,200–3,100 BCE	5,100	1.805	XX	K1a12a	–
Iran	sha009	Shahtepe, Iran	3,345–3,029 calBCE	5,137	0.250	XX	U5a2+16294	–
Iran	sha010	Shahtepe, Iran	3,200–3,100 BCE	5,100	1.400	XX	HV2	–
Iran	sha012	Shahtepe, Iran	3,200–3,100 BCE	5,100	1.075	XY	U1a3	J
Iran	sha014	Shahtepe, Iran	3,200–3,100 BCE	5,100	1.996	XY	HV13b	T1a

The “Date (BCE/CE)” column shows either calibrated C14 dates directly obtained from the samples (with the prefix “cal”) or approximate date intervals based on archaeological context (relative dating). The “Average Date (BP)” column shows dates before present (BP) calculated by adding 1,950 years to the average of the date intervals in the previous column of calibrated/context-based dates. “Sex” indicates genetic sex. See Tables S1 and S2 for more detailed information and Figure I in Document Z1 and Table Z9 in Zenodo for kinship results.

Caucasus and the Roman period in Anatolia. With the joint dataset, we called SNPs using a novel SNP panel, including 4.7 million SNPs (Dataset 1) ascertained in modern-day sub-Saharan African populations from the 1000 Genomes Project,[30] as well as the 1,240K (Dataset 2) and Human Origins (Dataset 3) SNP lists[31] (STAR Methods). We further sorted the dataset into temporal groups by dividing the Holocene into six time periods (TPs) (Figure 1; Table S2; STAR Methods; Table II in Document Z1).

To gain insight into general diversity patterns in this dataset, we performed principal component analysis (PCA) by projecting the 417 ancient genomes, including the 35 newly produced genomes from five regions, onto the PC space calculated using present-day West Eurasians (STAR Methods). This recapitulated geographic differentiation patterns, with PC1 being correlated with the north-south, and PC2 with east-west differentiation across different periods (Figure 2; see also Figures II–IV in Document Z1), implying some degree of geographic structure and regional continuity over time. We further tested these patterns using f_4-statistics.[32] Overall, we found a general trend for structure across Southwest Asia, with individuals generally sharing more alleles with local individuals than with individuals

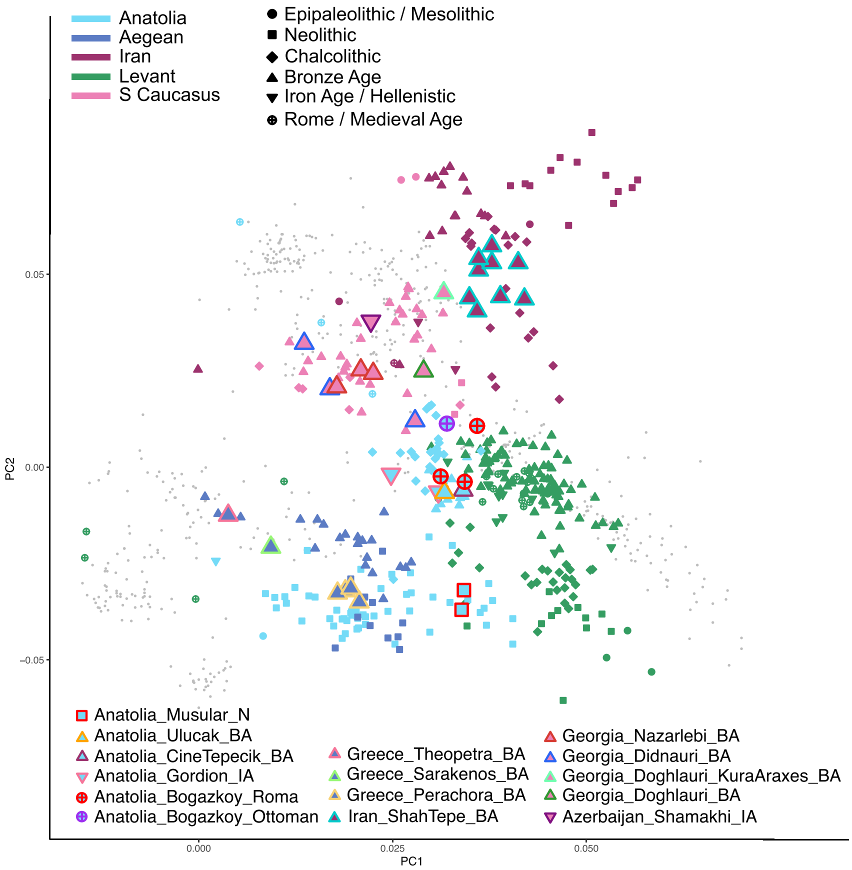

Figure 2. Principal component analysis (PCA)

The plot shows the first two principal components calculated using genomes of 828 individuals from 49 contemporary west Eurasian populations (Table Z1 in Zenodo), onto which a total of 417 ancient individuals were projected; here, we used the Human Origins SNP array (HO) SNP list (Dataset 3) (STAR Methods). Newly sequenced ancient individuals are highlighted by larger, color-framed symbols, while published individuals are shown with small symbols, and present-day individuals are depicted as the smallest gray points. Symbols indicate archaeological/historical periods and overlap multiple time periods described in Figure 1. N, Neolithic; BA, Bronze Age; IA, Iron Age (see also Figures II–IV in Document Z1, Figures Z1 and Z2 in Zenodo, and STAR Methods).

of other regions (Figure V and Table III in Document Z1). We also tested regional continuity by comparing regional gene pools across the six TPs. We found that each regional sample from a certain TP tended to share more alleles with the succeeding period than with later periods, supporting continuity (Figure VI in Document Z1). However, we also observed a number of shifts in regional gene pools, as we described in the following.

Inter-regional mobility as inferred from ancestry components

In the presence of overall regional genetic continuity, genomes from different TPs may be modeled as mixtures of early Holocene populations from Southwest Asia and the East Mediterranean as well as neighboring regions (e.g., East Europe or West Siberia). Changes in ancestry components through such modeling would then illustrate possible inter-regional gene flow events. We thus performed qpAdm modeling[33,34] on the newly generated and published ancient genomes from the five regions to describe changing sources of ancestry over time (Figure 3; Tables S3 and S4). In order to infer mobility from qpAdm results, we sought to explain ancestry components of each population as combinations of earlier regional populations, while noting that inferring mobility with this approach is based on the assumption of limited population structure within regions (see also Methods S1B). We further confirmed qpAdm-estimated ancestry change patterns using f_4-statistics (Figures S1–S4; Tables S3 and S4).

Anatolia

We start with the Anatolian peninsula, the approximate center of our region of interest. The new genomes from Musular (n = 2; ca. 9,100 BP) of Central Anatolia can be modeled as earlier Aceramic Neolithic Central Anatolian genomes with additional southern (Levant-related) and eastern (Zagros/Caucasus-related) ancestry components (Figures 3 and S1; Tables S3 and S4). This profile closely resembles that of ca. 8,500 BP Çatalhöyük, which suggests that putative eastern/southern gene flow into Central Anatolia[14,17,22,35] had taken place already by the late 10th millennium BP, before the Ceramic Neolithic. Meanwhile, DATES[36] estimation of admixture times did not yield realistic or technically feasible results (Table IV in Document Z1). In the post-Neolithic period, we present new genomes from Ulucak (n = 1) and Çine-Tepecik (n = 1) of BA West Anatolia, from Gordion (n = 2) of Central Anatolia in the IA/Hellenistic period, and from Boğazköy in Central Anatolia dating to the Roman (n = 3) and Ottoman (n = 1) periods. Interestingly, all 8 genomes can be modeled as admixtures between Ceramic Neolithic/early Chalcolithic Anatolia (ca. 70%–80%) and Zagros/Caucasus-related ancestry sources (ca. 20%–30%) (Figure 3). This is highly similar to published BA Central and West Anatolian genomes, which were earlier described as being admixed between local Neolithic and eastern sources.[18,20,37] The observation that ancestry components in Anatolia changed little from the BA to the Roman or even Ottoman periods suggests the apparent stability of the gene pool through four millennia, also observed in a recent study.[38] Exceptions include a published Kalehöyük IA genome carrying European ancestry (not observed in later-coming genomes), and Kalehöyük Ottoman genomes carrying Baikal Neolithic-related ancestry, likely representing Turkic admixture in the 1st millennium BP. Meanwhile, our Boğazköy Ottoman genome hints at the heterogeneity of this Baikal-related admixture in Anatolia; this heterogeneity can still be observed in modern-day Turkish genomes[39] (Figures 3 and S1).

Aegean

Recent studies showed that Neolithic populations in modern-day Greece were genetically similar to Anatolian contemporaneous

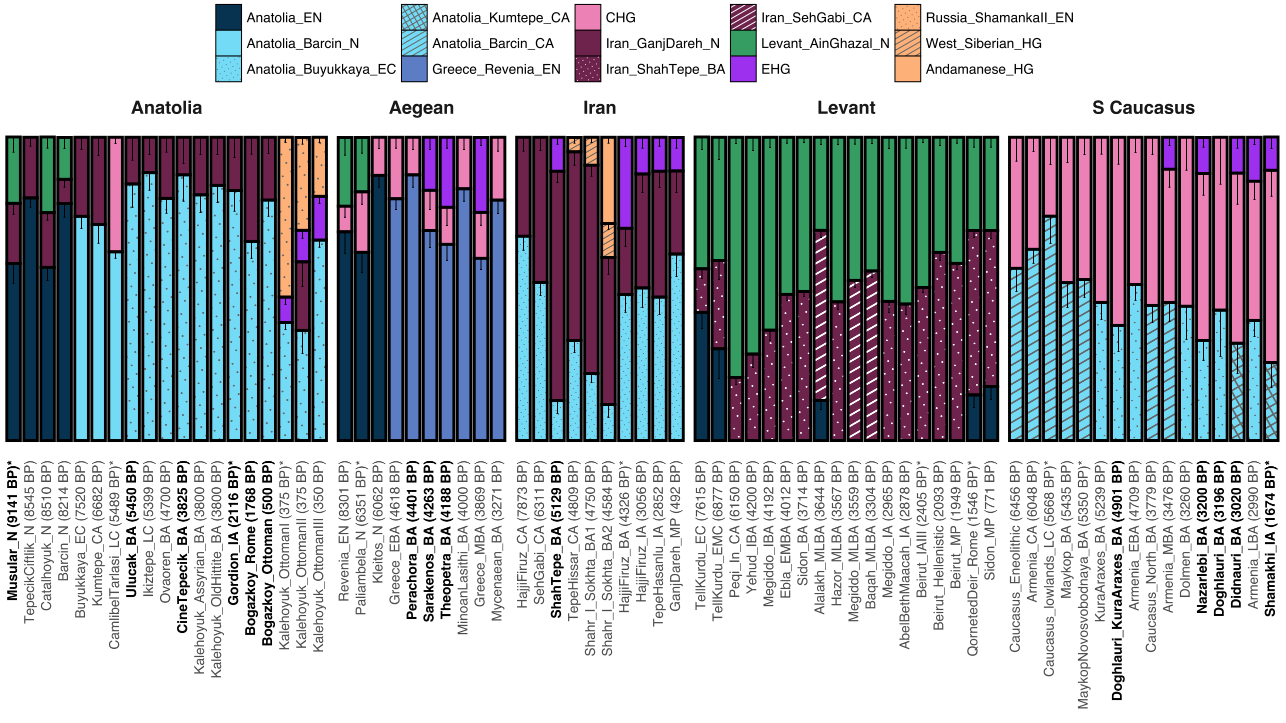

Figure 3. qpAdm models for Neolithic and post-Neolithic populations of Southwest Asia and the East Mediterranean

Each modeled genome or group of genomes is represented by columns in temporal order; the average dates are indicated in parentheses. Full population labels are listed in Table S2. Newly generated samples are highlighted in bold on the x axis. All source populations are color coded and shown above the figure. Vertical bars represent the coefficients of source populations. Error bars show one standard error. The models that yielded p > 0.01 are shown with an asterisk (*), and all other models yielded p > 0.05 (see Harney et al.[34] for an interpretation of p values in qpAdm analyses). All models tested are reported in Table Z3 in Zenodo. Among alternative feasible models, we selected one per genome to represent in this figure, following criteria we describe in STAR Methods (e.g., using as few sources as possible and using preferentially the same sources for genomes from the same region). We note that alternative feasible admixture models (with alternative source populations) listed in Table Z3 and not presented in the figure also support the same conclusions described in the text (Methods S1C). Population labels include the following abbreviations: HG, Hunter-Gatherer; (E)N, (Early) Neolithic; (E/L)C/CA, (Early/Late) Chalcolithic; (EM/M/I/L)BA, (Early Middle/Middle/Intermediate/Late) Bronze Age; IA, Iron Age; MP, Medieval Period; CHG, Caucasus HG; EHG, East European HG. Dataset 1 (STAR Methods) was used for qpAdm modeling.

See also Tables S3 and S4, Figures S1–S4, Methods S1, and STAR Methods.

populations,[10,17,18,40] while during the transition to the BA the Aegean received gene flow from eastern (South Caucasus/Iran-related) and, later, Eastern hunter-gatherer (EHG)-related sources.[13,18] Accordingly, we could model our new genomes from Perachora cave (n = 5), Sarakenos cave (n = 1), and Theopetra cave (n = 1) in mainland Greece via two- or three-way mixture models of Aegean Neolithic-related populations (60%–83%), Caucasus/Zagros-related populations (12%–20%), and EHG-related populations (0%–25%) (Figures 3 and S2). This confirms the earlier observation of a gradual and partial diffusion of EHG-related ancestry in present-day Greece.[13,18] Our results from the Sarakenos cave further push the hypothesized arrivals of people with Steppe-related ancestry in the Greek mainland into ca. 4,200 BP, within the Early BA, i.e., before the beginning of the Middle BA as hitherto known.[13] Although this is currently the earliest known evidence for Steppe-related ancestry in Greece, the hypothesis of an even earlier arrival of these people remains to be tested on new ancient genomes from the region. We note that DATES[36] estimation of admixture times were again not feasible (Table IV in Document Z1).

Zagros/Iran

Within the region corresponding to modern-day Iran, including the Zagros range, regional populations were previously shown to receive gene flow from both western (Anatolia-related) sources, starting with the Neolithic period, followed by northern (EHG- or Siberia-related) sources during the BAs, most likely representing mobility from Central Asia.[11,19] Our new genomes from Shah Tepe (n = 9; ca. 5,100 BP), from Northeast Iran near the Caspian Sea, could likewise be modeled as admixtures of Zagros Neolithic-related (76%), Anatolia Neolithic-related (13%), and EHG-related (11%) ancestries (Figures 3 and S3). Notably, Anatolian-related ancestry was lower in Shah Tepe relative to Zagros populations (Tepe Hissar and Hajji Firuz), in support of a west-to-east gradient of Anatolian admixture.[19] Further, the Shah Tepe genomes present the earliest indication of EHG-related ancestry in Iran, which is consistent with material culture records from Northeast Iran during the Chalcolithic and BAs showing Central Asian cultural influences, including in Shah Tepe[41,42]; this supports the notion of EHG influx in Iran via Central Asia instead of the Caucasus. Our modeling further marks the heterogeneity of ancestry sources across Iran, including the temporary appearance of South Asian (Andamanese Hunter-Gatherers [HG]-related) ancestry in the southeastern site of Shahr-i Sokhta during the BA[19] (Figures 3 and S3).

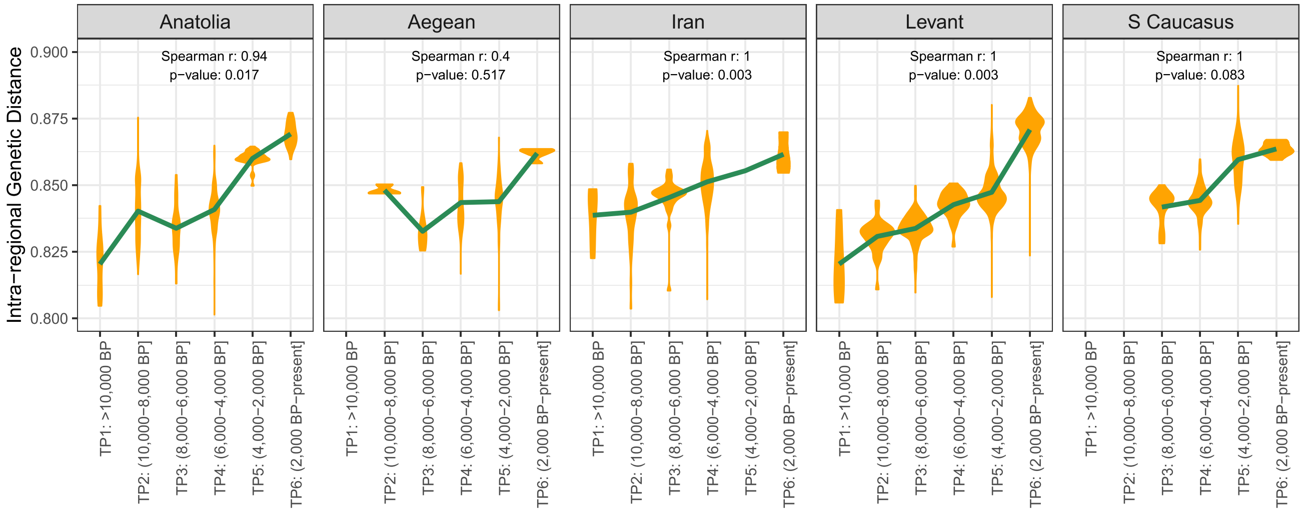

Figure 4. Genetic diversity in Southwest Asia and the East Mediterranean over time

The violin plots show genetic diversity calculated as pairwise genetic differences ($1 - f_3$) among individuals in a group. The Spearman's coefficients and p values of the correlation between average genetic distance and time periods are shown on top of each panel. All f_3-statistics were calculated using pairs with at least 2,000 overlapping SNPs between them (see Figures X and XI for results using higher SNP cutoffs, Figures XIII and XIV for alternative periodization schemes, and Figure IX for the same calculation using X-chromosome SNPs in Document Z1). Dataset 1 (STAR Methods) was used in the analysis. Standard errors of distance estimates (calculated using jackknifing; STAR Methods) are <0.01 and are thus not visible on the graph.

South caucasus

Previous work described the influx of Anatolian Neolithic-related ancestry in the South Caucasus with the arrival of Neolithic cultures (ca. 8,000 BP)[11,21] (also see Figure S4). Our new genomes from Doghlauri, Georgia, belonging to the Early BA Kura Araxes culture (n = 1; ca. 4,900 BP)[43] and to the Late BA (n = 1; ca. 3,200 BP) can likewise be modeled as two-way admixtures of local CHG (62%–57%) and Anatolian Chalcolithic populations (38%–43%) (Figures 3 and S4). Meanwhile, by ca. 3,750 BP, EHG-related ancestry appears in the sample of Armenia Middle BA.[21] We similarly find EHG-related ancestry in three new Didnauri BA and three new Nazarlebi BA genomes (ca. 3,000 BP) from Georgia (12%), as well as the new IA genome (ca. 1,700 BP) from Shamakhi in Azerbaijan (11%). This suggests that EHG-related gene flow had a persistent impact in the regional gene pool (Figure 3).

Levant

Temporal changes in ancestry components in the Levant during the Holocene have been investigated in detail, and post-Neolithic Levant genomes could be modeled as two- or three-way admixtures of local Levant Neolithic populations and post-Neolithic populations from Iran and/or Anatolian Neolithic populations, with variable degrees.[8,11,15,20,24,44] Our modeling of published data confirmed this general description (Figure 3; Table S4). We note that alternative models using external sources such as EHG have also been proposed[16,44] and that two specific ancient genome samples, those from BA and IA Ashkelon[26] and those representing Medieval Crusaders,[44] both carrying high degrees of West European ancestry, appear not to have left permanent signatures in the local gene pool (STAR Methods).

Genetic diversity increases monotonously over time

Two observations arise from the above qpAdm and f_4-statistics results (Figures 3 and S1–S4; Tables S3 and S4). First, the Neolithic and Chalcolithic periods appear dominated by increased sharing of ancestry components across regions, such as Anatolian/Aegean-related ancestry in Iran, Caucasus, and Levant, and Zagros/Caucasus-related ancestry in the Levant, Anatolia, and the Aegean. Under the assumption of limited population structure within each region (see also Methods S1B), this suggests inter-regional gene flow *within* Southwest Asia, in line with the homogenization model.[7,8,11,38] This process can also be followed on the PCA, such that genomes of different regions appear to converge in PC space over time (Figure III in Document Z1). Second, genomes from the BA onward include geographically more distant ancestry components, such as East Europe, West Siberia, the Baikal, or South Asia. These latter components are sometimes transient, such as the Medieval Crusaders in the Levant,[44] while others are persistent and detectable in subsequent genome samples from the same region.

Both internal homogenization and distant interaction should elevate within-population genetic diversity. To test this idea, we estimated diversity per region and TP by calculating pairwise genetic differences ($1 - f_3$) between individuals within a group. We observed monotonous and significant trends of increasing diversity through the Holocene (Spearman's correlation between diversity and time per region: rho > 0.94, one-sided $p < 0.04$), and a non-significant trend in the Aegean (Figure 4). Although temporal increases in diversity had previously been noted for West Eurasian populations,[45] such monotonous change has not been reported, to our knowledge. The observed diversity increase is best attributed to some degree of migration into each region (i.e., migrants with non-local genetic ancestries breeding with locals and elevating diversity). *De novo* variants cannot be the source of this signal, as we use SNPs ascertained in an outgroup population equally distant to our groups of interest[45] (STAR Methods; Methods S1A).

We further tested the pattern of increasing diversity through runs of homozygosity (ROHs) estimated by hapROH[46]

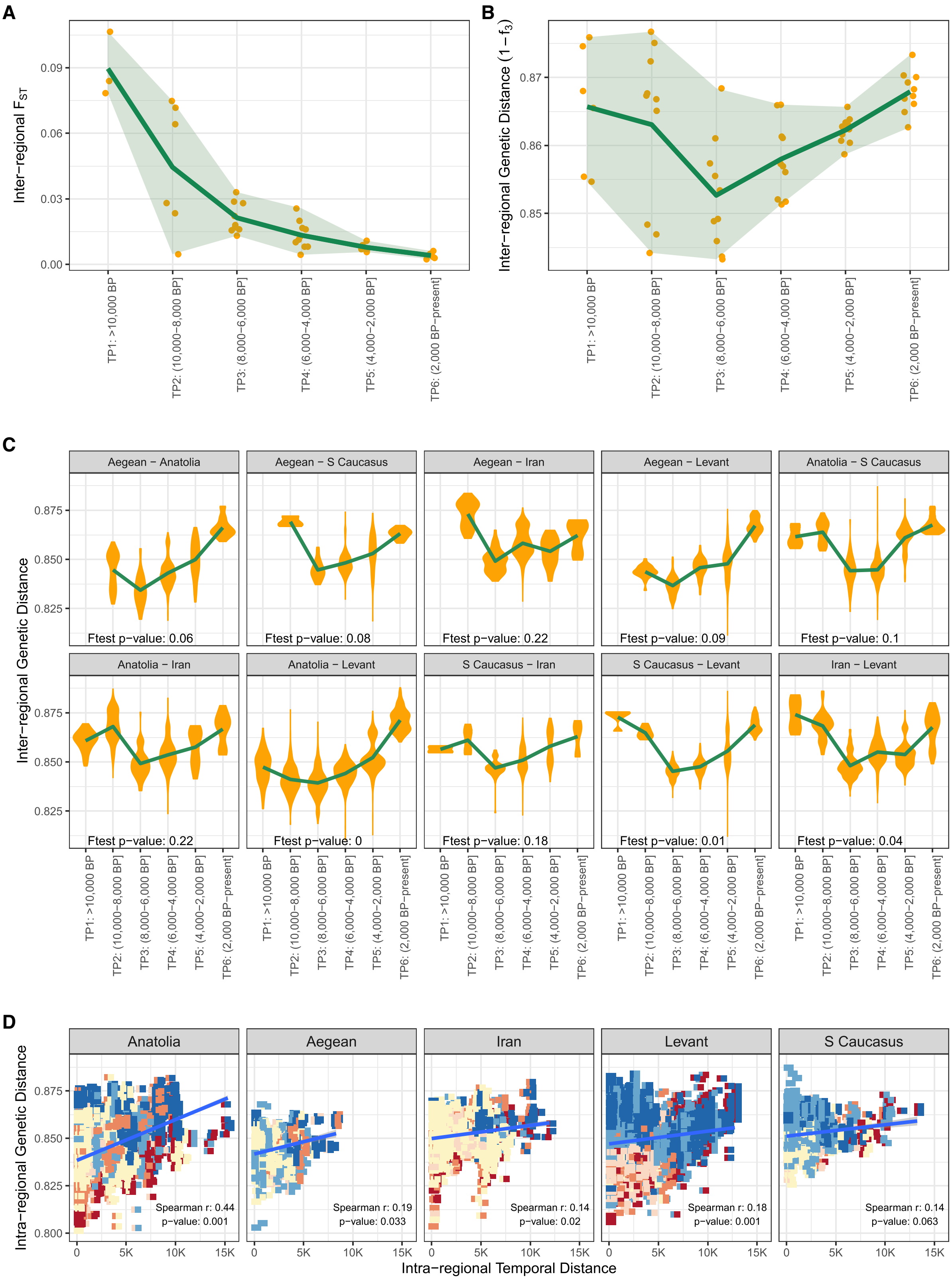

Figure 5. Inter-regional genetic differentiation over time in Southwest Asia and the East Mediterranean

(A and B) The points show (A) pairwise F_{ST} and (B) pairwise $1 - f_3$ values calculated among regional populations belonging to each time period, while the green line indicates the mean. The areas between lower and upper bounds of each time period are shaded.

(legend continued on next page)

CellPress
OPEN ACCESS

(STAR Methods). Excluding potentially consanguineous genomes, the average sum of relatively short (4–8 cM) ROHs tended to decrease in four regions (except the Levant), and this trend was significant in Anatolia (Spearman's rho = −0.61, p = 0.005; Figure S5). This is again compatible with an increase in the within-region genetic diversity due to admixture.[6,46,47]

The expanding nature of inter-regional mobility

We then investigated the question of inter-regional genetic divergence through the Holocene. Using 417 ancient and 23 modern individuals from Southwest Asia and the East Mediterranean, we first recapitulated the reported signal of decreasing average pairwise F_{ST} among regions[7,8,11] over the 6 TPs (Spearman's rho = −1, p = 0.002) (Table S2; Figure 5A). This is particularly strong in the early Holocene when regional gene pools homogenize and is also observed in ancestry components (Figure 3).

However, interpreting the pairwise F_{ST} signal in the context of mobility can be difficult, as this statistic is affected by within-population diversity, which in turn can be influenced by population size changes or gene flow from third sources.[48] Instead, the outgroup-f_3-statistic can be a more effective tool for inferring gene flow between two groups, as it measures shared drift between two genomes relative to an outgroup; it is thus robust to population size and diversity changes within groups.[32,49,50] We tested this expectation through coalescent simulations and confirmed that outgroup-f_3 (but not F_{ST}) can readily capture gene flow between two groups while not being affected by bottlenecks (STAR Methods; Figure VII in Document Z1 and Table Z2 in Zenodo).

Using the $(1 - f_3)$ distance to measure pairwise genetic differentiation among the five regional groups revealed a different pattern from that of F_{ST}: average inter-regional genetic differentiation decreases until 6,000 BP and then rises again (Figure 5B). The concave-up (down-up) shape of the average differentiation-time trajectory was marginally significant over a linear model (F-test, p = 0.04; see also Figures VIII–XVI in Document Z1; STAR Methods). We repeated the analysis by calculating pairwise genetic distances between individuals (instead of grouping them as regional populations), which again revealed a concave-up differentiation pattern (in 7 out of 10 comparisons: F-test, $p \leq 0.10$) (Figure 5C; Methods S1C). Alternative periodization schemes do not alter these main findings (Figures XIII and XIV in Document Z1). Meanwhile, the reason F_{ST} tends to decrease in the late Holocene while $(1 - f_3)$ increases can be attributed to the increase of within-population diversity in the same period (Figure 4). We could replicate this contrasting behavior between F_{ST} and f_3-based distance in coalescent simulations by introducing gene flow from external sources (Model G of Figure VII in Document Z1).

These observations suggest two sequential processes. The first involves intense mobility within Southwest Asia and the East Mediterranean after the Neolithic transition, in the early half of the Holocene. This is also evident in the qpAdm results (Figure 3): for instance, up until 6,000–4,000 BP, Anatolian and Aegean populations received intense gene flow from South Caucasus/Iran-related populations, while groups from Caucasus and Iran received gene flow from Anatolian-related populations. Similar patterns have also been reported in a recent analysis of the demographic history of the region.[38] These putative admixture events could explain a reduction in genetic distance supported both by F_{ST} and $(1 - f_3)$ values, and may also be inferred in the PCA (Figure III in Document Z1; Methods S1D). The second inferred process involves external gene flow. After the 6,000–4,000 BP period, populations in all five regions likely received different degrees of gene flow from regions outside of Southwest Asia and the East Mediterranean. Examples include EHG/Steppe-related ancestry in the Aegean, South Caucasus, and Levant[13,16]; EHG- and Central-Asian-related ancestry in Anatolian IA and later genomes[12]; Western hunter-gatherer (WHG)-, South-Asian-, and Central-Asian-related ancestries in Levant Medieval populations[16,44]; and West-Siberian-related and South-Asian-related ancestry in Iran[19] (Figures 3 and S1–S4). As a consequence of these inferred long-distance mobility events, inter-regional genetic differentiation in Southwest Asia, calculated as $(1 - f_3)$, rebounds over time (while F_{ST} remains low due to increasing intra-regional diversity). We call this "the expanding-mobility model."

Spatial heterogeneity in mobility levels

An intriguing pattern in Figure 4 was the ostensible regional differences in time-dependent diversity changes, such as higher magnitudes of change in Anatolia. We explored this further by calculating genetic distances $(1 - f_3)$ between *all* pairs of individuals from a region (irrespective of the TP) and then calculating the correlation between pairwise genetic distance versus time difference. This yields an estimate of Holocene-wide temporal differentiation in the gene pool of a region.

In all five regions, we found positive correlations between genetic distance and separation time (each region: Spearman's rho = 0.14–0.44, permutation test, p < 0.06; Figure 5D). Anatolia exhibits the highest change, similar to the diversity analysis above. We repeated this analysis using X chromosome SNPs (Figure IX in Document Z1), using subsets of individuals with similar numbers and/or temporal distributions across the five regions (Figures XVII and XVIII in Document Z1), or using only SNP capture- or shotgun-sequenced genomes (Figures XIX and XX in Document Z1). In all analyses, except when only shotgun-sequenced genomes were used (Figures XIX and XX

(C) The violin plots show pairwise genetic distance $(1 - f_3)$ between regions, calculated by comparing all individuals between a pair of regions within each time period, and the green lines show the mean.
(D) Genetic distances $(1 - f_3)$ (y axis) versus time differences (x axis) among all pairs of individuals within each region. Each point (a rectangle consisting of two squares) represents a pair of ancient individuals, with the squares colored according to the respective time period (see Figure 1). The line represents linear regression. The Spearman correlation coefficient between time and distance, and the p value calculated by random permutations of individuals across time (n = 1,000), are indicated on the figure. All analyses in the figure were performed using autosomal SNPs in Dataset 1 (STAR Methods), and all f_3-statistics were calculated using pairs with at least 2,000 overlapping SNPs between them (see Figures X and XI for results using higher SNP cutoffs, Figures XIII and XIV for alternative periodization schemes, and Figure IX for the same calculation but using X-chromosome SNPs in Document Z1). Standard errors of both for F_{ST} and outgroup-f_3-based distance estimates (calculated using jackknifing; STAR Methods) are <0.01 and are thus not visible on the graph.
See also Methods S1 and STAR Methods.

in Document Z1), Anatolia showed the strongest magnitude of change. If this result proves robust in future work, it would be tempting to investigate whether geographic factors, such as Anatolia being *en route* between Europe and Southwest Asia, or comprising large arable lands that could sustain sizeable populations—or idiosyncratic events, such as the strong East/Central Asia-related gene flow event into Anatolia over the last millennium—could have contributed to the relatively high rate of change on the peninsula. Conversely, the Caucasus shows a relatively low magnitude of genetic change in most analyses, which may have been shaped by terrain ruggedness and/or lower carrying capacities.[51] The limited consistency among datasets, however, indicates that our estimate of overall temporal differentiation may be sensitive to technical factors, such as sequencing technology and SNP panels used.

A possible temporal shift in sex-biased inter-regional mobility

Finally, we addressed the question of sex-biased mobility in Southwest Asia and the East Mediterranean. We first analyzed the distribution of mtDNA and Y chromosome haplogroups between consecutive TPs using F_{ST}. We observed no significant difference in mtDNA haplogroup composition but a number of significant temporal shifts in Y chromosome haplogroup composition (Figure 6A). Although this analysis is compromised by the partly arbitrary nature of haplogroups, it does imply the relative stability of the maternal gene pool, consistent with earlier work in various regions.[27–29] It would also be compatible with stronger genetic drift in the male gene pool and/or higher rates of male mobility, with the most notable effect in the Levant (Figures XXI–XXIII in Document Z1).

We next investigated genetic change on autosomes versus the X chromosome to gain further insight into possible sex-biased gene flow. For this, we calculated the genetic distance between consecutive TPs within each region, for autosomes and the X chromosome separately. These autosomal and X chromosomal distances were highly correlated, as expected (Spearman's rho = 0.82, $p < 0.0001$) (Figure 6B). Interestingly, in early periods, genetic distances on the X chromosome increased significantly more than on the autosomes, and vice versa in later periods. Residuals from an autosomal versus X chromosomal distance regression model were hence highly positively correlated with time (Spearman's rho = 0.70, $p = 0.0001$; Figure 6C) (Figures XXIV and XXV in Document Z1). We can rule out differential drift between male and female gene pools as the cause of this signal due to the insensitivity of the f_3-statistic to drift in this context (Figure VII in Document Z1). This suggests that sex bias in admixture events shifted over time. This can be caused by female mobility being relatively higher during early periods than later periods and/or by higher reproductive success of migrant males in later than earlier periods (Figure 6C).

This putative shift in sex-biased admixture patterns resembles observations in ancient Europe, with low sex bias in the Neolithic expansion followed by highly male-biased Steppe expansion in the BA.[13,52,53] A time-dependent increase in sex bias would also be consistent with the expanding-mobility model, given observations that long-range human migration may tend to be more male-dominated than short-range migration.[54] Meanwhile, we cannot directly quantify sex bias in this framework; i.e., we cannot distinguish whether early periods were devoid of sex bias and male bias emerged later, or early periods had female bias that later disappeared. In addition, the haplogroup composition analysis described earlier provides only weak parallels to the observation of temporal shifts in male bias (Figure 6A). Although we remain cautious about the generality of the observed sex-biased mobility patterns, we note that their study can provide vital insight into changing social dynamics and networks over time.

DISCUSSION

Our work reveals a number of novel observations. We show that rates of inter-regional genetic differentiation, as measured by $(1 - f_3)$, did not decline through the Holocene in Southwest Asia and the East Mediterranean, in contrast to the implications of earlier F_{ST} analyses. On the contrary, while intra-regional diversity increases monotonously over time, inter-regional differentiation first declines and then rebounds, approximately starting with the BA. We find that these patterns are generally robust to technical factors, such as experimental protocol (SNP capture versus shotgun sequencing) (Figures XIX and XX in Document Z1) and SNP numbers used (Figures X and XI in Document Z1; Methods S1C). These results suggest that mobility continued unabated and also with an expanding range, possibly both as a result and a consequence of increasing social and technological complexity (Table I in Document Z1). We also observe a trend of increasing male bias in mobility in the latter half of the Holocene, partly reminiscent of sex-biased mobility observed in European history.[52]

These changing patterns in mobility, inferred from diversity and divergence statistics, resonate well with archaeological and historical evidence regarding improvements in the means of transportation (e.g., horses and roads), the expanding scales of exchange networks (e.g., long-distance trade of raw material and produce, including the establishment of trade routes and trade colonies), and the trend toward more hierarchical and centralized polities able to exert an influence over larger territories and populations (e.g., organized invasions and forced displacements) that emerge in the second half of the Holocene in Southwest Asia and the East Mediterranean (Table I in Document Z1). An attractive question for future studies would be whether this pattern of expanding mobility ranges in post-Neolithic societies may also be observed in other regions, such as South and East Asia, Africa, or the Americas.

We acknowledge that due to the patchy distribution of our sample and the limited number of available genomes in several regions and TPs (Figure 1; Table II in Document Z1), some of our observations on trans-regional patterns may be considered tentative. Denser and more homogeneous samples will allow possible confounding between population structure and temporal changes to be strictly ruled out. Nevertheless, the fact that we detect consistent trends across all five regions and that we replicate our results in bootstrap and jackknife analyses (Figures XV–XVIII, XXVI, and XXVII and Table V in Document Z1), and using alternative periodizations (Figures XIII and XIV in Document Z1), overall suggest the robustness of our main

CellPress
OPEN ACCESS

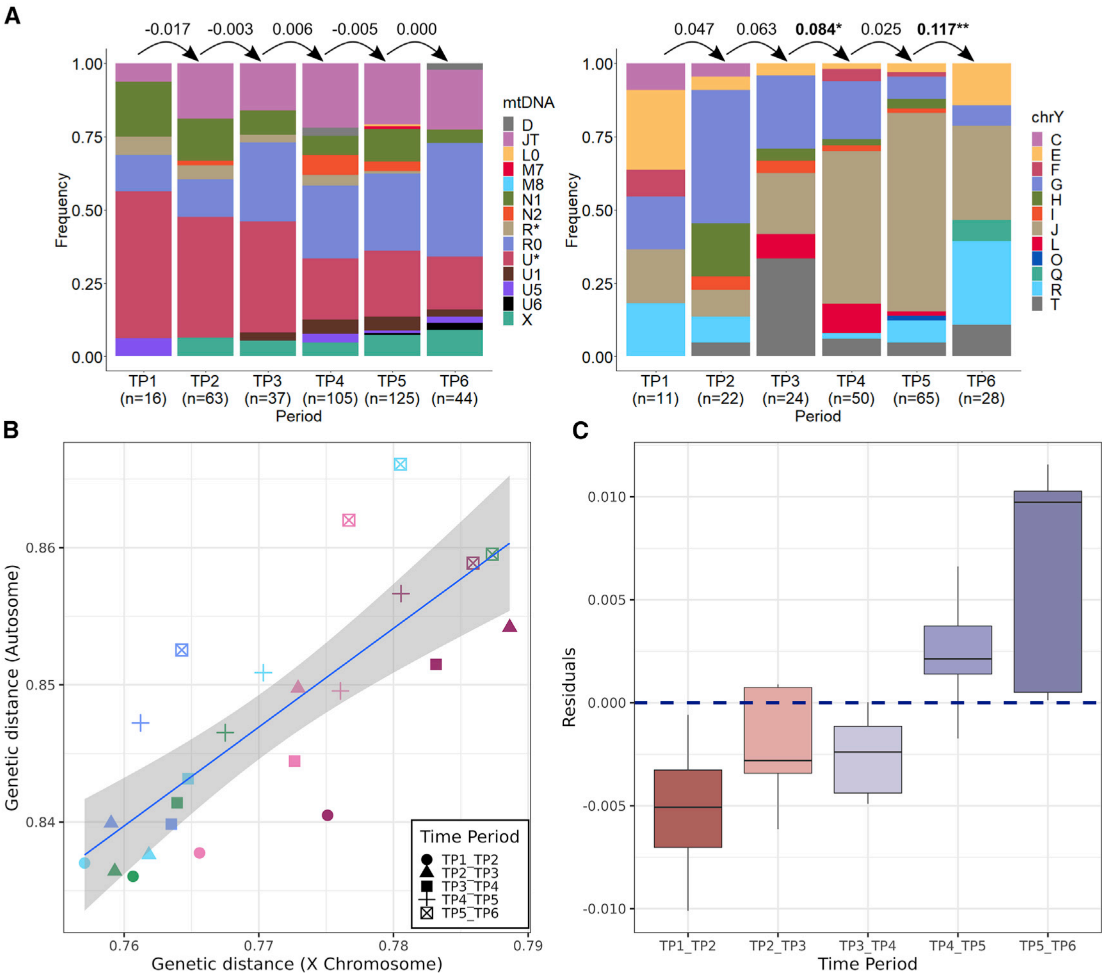

Figure 6. Uniparental markers and sex-biased admixture

(A) Distribution of mtDNA and Y chromosome haplogroups among time periods across all regions of Southwest Asia and the East Mediterranean. The values between the bars are F_{ST} values, with negative values indicating practically no differentiation. Bold values indicate nominally significant F_{ST}-based differentiation between consecutive periods (permutation test, $p < 0.05$; see also Table Z8 in Zenodo and Figures XXII–XXIIII in Document Z1 for an alternative haplogroup classification).

(B) Comparison of average genetic distance ($1 - f_3$) between two consecutive time periods in the same region, calculated using autosomes (y axis) versus the X chromosome (x axis). We used Dataset 1 in this analysis and applied a cut-off of >2,000 SNPs for calculations for autosomal SNPs and >1,000 SNPs for X-chromosome SNPs. Each point represents the average genetic distance between genome samples from two consecutive time periods of the same region, i.e., a measure of within-region genetic change. Comparisons involving the first half of the Holocene (TP1–TP2, TP2–TP3, TP3–TP4) are below the regression line, indicating relatively more change on the X chromosome than on autosomes. In contrast, comparisons involving the latter half of the Holocene (TP4–TP5, TP5–TP6) tend to be above the line, indicating *relatively* more change on autosomes.

(C) Distribution of residuals obtained from the linear regression model in (B) (n = 24). Residuals and time were highly correlated (Spearman's rho = 0.70, p = 0.0001).

See also Figures XXIV–XXVII and Table V in Document Z1.

observations, i.e., intra-regional genetic diversity increasing over time, shifting sex bias in admixture, and inter-regional genetic distances increasing with the BA. The latter observation is also supported by our qpAdm results as well as those recently published by independent groups.[38]

Finally, our statistics are only indirect measures of human mobility, and the absolute magnitudes of these movements remain uncertain. This is because the amount of observed change in outgroup-f_3 (Figure 5B) or ROH (Figure S5) values will depend not only on the migration rate (the proportion of incoming migrant alleles in the gene pool each generation) but also on the amount of genetic differentiation between incoming and local groups.[55] In addition, if one takes into account the fact that human populations in Southwest Asia and the East Mediterranean grew significantly over the Holocene,[56] the absolute amount of human movement (immigrant numbers) required to create a certain magnitude of change will also vary in time. Accordingly, our observation that diversity increased linearly in time cannot be interpreted as an indication of constant migration levels through the Holocene. Quantifying the exact amount of mobility thus remains a future challenge.

STAR★METHODS

Detailed methods are provided in the online version of this paper and include the following:

- KEY RESOURCES TABLE
- RESOURCE AVAILABILITY
  - Lead contact
  - Materials availability
  - Data and code availability
- EXPERIMENTAL MODEL AND SUBJECT DETAILS
  - Description of archaeological sites and archaeological material
- METHOD DETAILS
  - Sample preparation
  - Radiocarbon dating
- QUANTIFICATION AND STATISTICAL ANALYSIS
  - Sequence data processing
  - Testing for contamination and quality control
  - Molecular sex determination
  - Estimating uniparental haplogroups
  - Genome-wide SNP datasets
  - Ancient genome sample selection
  - Defining time periods
  - Trimming and pseudo-haploid genotyping
  - Genetic kinship analyses
  - Principal components analysis (PCA)
  - Ancestry proportion estimation
  - Genetic differentiation among populations
  - Genomic similarity/distance among populations
  - Detecting gene flow among populations
  - Admixture time estimation
  - Runs of homozygosity (ROH)
  - Coalescent simulations
  - Visualization

SUPPLEMENTAL INFORMATION

Supplemental information can be found online at https://doi.org/10.1016/j.cub.2022.11.034.

ACKNOWLEDGMENTS

We thank Orhan Efe Yavuz, Anna-Sapfo Malaspinas, Flora Jay, Ayshin Ghalichi, Yeşim Aydın Son, Can Alkan, Hamit İzgi, Aslıhan Ilgaz, Maja Krzewińska, and all colleagues at the METU CompEvo, Hacettepe Human_G and Center for Palaeogenetics (CPG) for their support, suggestions, and/or comments. We also thank three anonymous reviewers for their constructive criticism. The authors acknowledge support from Wenner-Gren Foundation Dissertation Fieldwork grant (no. 9573 to D. Koptekin); H2020 ERC Consolidator grant (no. 772390 NEOGENE to M.S.); EMBO Scientific Exchange grant (no. 8883 to D. Koptekin); H2020-WIDESPREAD-05-2020 TWINNING grant (no. 952317 NEOMATRIX to M.S.); TÜBİTAK of Turkey (no. 117Z229 to M.S.); Klaus-Tschira Foundation; German Research Foundation (DFG, grant no. SCHA 889/4-4 to A. Schachner); the National Genomics Infrastructure in Stockholm funded by Science for Life Laboratory, the Knut and Alice Wallenberg Foundation, and the Swedish Research Council; SNIC/Uppsala Multidisciplinary Center for Advanced Computational (UPPMAX) projects uppstore20180102, SNIC 2018/8-293, SNIC 2018/8-88, and SNIC 2021-2-17; the projects HELLAS-CH (MIS 5002735), implemented under Action for Strengthening Research and Innovation Infrastructures, and POLITEIA-II (MIS 5002478), implemented under Action for the Strategic Development on the Research and Technological Sector, both funded by the operational programme Competitiveness, Entrepreneurship and Innovation (NSRF 2014–2020) and co-financed by Greece and the European Union (European Regional Development Fund); and Hacettepe University Scientific Research Projects Coordination Unit (nos. 16769 and 14528 to A.M.B.). Computations were performed at NEOGENE (Middle East Technical University) and UPPMAX resources.

AUTHOR CONTRIBUTIONS

Conceptualization, D. Koptekin, M.S., A. Götherström, F.Ö., and J.S.; methodology, D. Koptekin, M.S., A. Götherström, P.P., and E.Y.; formal analysis, D. Koptekin, E.Y., N.E.A., M.S., R.R.-V., K.G., K.B.V., N. Psonis, and E.K.; investigation, F.Ö., R.R.-V., N.K., D. Koptekin, S.Y., R.G., D.D.K., D. Kaptan, N. Psonis, D.V., V.K.L., E.D., A.M., and E.T.; resources, Ö.D.E., Y.S.E., A. Schachner, H.Ü., R.S., M.Ö., G. Duru, E.S., C.B.R., L.B., M.E., A. Gavashelishvili, K.P., N. Psonis, T.G., G. Darbyshire, N.K.-A., Ö.Ç., O.V., A.M.B., U.O., S.G., A.A., A.I., and V.S.; writing – original draft, D. Koptekin and M.S.; writing – review & editing, J.S., A. Götherström, F.Ö., H.C.G., R.R.-V., C.K., E.S., D. Kaptan, K.G., G.M.K., M.Ö., G. Duru., A. Schachner, N. Poulakaki, R.G., C.B.R., A. Gavashelishvili, P.P., N.E.A., R.S., A. Stamatakis, A.N., N. Psonis, Ö.Ç., E.Y., A.M.B., S.G., V.K.L., K.B.V., D.V., Ç.A., and E.T.; visualization, D. Koptekin, N.E.A., and E.Y.; funding acquisition, M.S., D. Koptekin, A. Schachner, A. Götherström, A.M.B., and E.K.; supervision, M.S., A. Götherström, P.P., F.Ö., Y.S.E., A. Stamatakis, and N. Poulakaki.

DECLARATION OF INTERESTS

The authors declare no competing interests.

Received: April 29, 2022
Revised: August 13, 2022
Accepted: November 15, 2022
Published: December 8, 2022

REFERENCES

1. Bertman, S. (2003). Handbook to Life in Ancient Mesopotamia (Oxford University Press).
2. Lewis, B. (1995). The Middle East. A Brief History of the Last 2,000 Years (Scribner).
3. Van De Mieroop, M. (2007). A History of the Ancient Near East, ca. 3000–323 BC, Second Edition (Wiley-Blackwell).
4. Palmisano, A., Woodbridge, J., Roberts, C.N., Bevan, A., Fyfe, R., Shennan, S., Cheddadi, R., Greenberg, R., Kaniewski, D., Langgut, D., et al. (2019). Holocene landscape dynamics and long-term population trends in the Levant. Holocene *29*, 708–727. https://doi.org/10.1177/0959683619826642.
5. Potts, D.T. (2012). A Companion to the Archaeology of the Ancient Near East, First Edition (Wiley-Blackwell).
6. Ceballos, F.C., Gürün, K., Altınışık, N.E., Gemici, H.C., Karamurat, C., Koptekin, D., Vural, K.B., Mapelli, I., Sağlıcan, E., Sürer, E., et al. (2021). Human inbreeding has decreased in time through the Holocene. Curr. Biol. *31*, 3925–3934.e8. https://doi.org/10.1016/j.cub.2021.06.027.
7. Feldman, M., Gnecchi-Ruscone, G.A., Lamnidis, T.C., and Posth, C. (2021). Where Asia meets Europe – recent insights from ancient human genomics. Ann. Hum. Biol. *48*, 191–202. https://doi.org/10.1080/03014460.2021.1949039.
8. Harney, É., May, H., Shalem, D., Rohland, N., Mallick, S., Lazaridis, I., Sarig, R., Stewardson, K., Nordenfelt, S., Patterson, N., et al. (2018). Ancient DNA from Chalcolithic Israel reveals the role of population mixture in cultural transformation. Nat. Commun. *9*, 3336. https://doi.org/10.1038/s41467-018-05649-9.
9. Kılınç, G.M., Omrak, A., Özer, F., Günther, T., Büyükkarakaya, A.M., Bıçakçı, E., Baird, D., Dönertaş, H.M., Ghalichi, A., Yaka, R., et al. (2016). The demographic development of the first farmers in Anatolia. Curr. Biol. *26*, 2659–2666. https://doi.org/10.1016/j.cub.2016.07.057.

CellPress
OPEN ACCESS

10. Marchi, N., Winkelbach, L., Schulz, I., Brami, M., Hofmanová, Z., Blöcher, J., Reyna-Blanco, C.S., Diekmann, Y., Thiéry, A., Kapopoulou, A., et al. (2022). The genomic origins of the world's first farmers. Cell *185*, 1842–1859.e18. https://doi.org/10.1016/j.cell.2022.04.008.

11. Lazaridis, I., Nadel, D., Rollefson, G., Merrett, D.C., Rohland, N., Mallick, S., Fernandes, D., Novak, M., Gamarra, B., Sirak, K., et al. (2016). Genomic insights into the origin of farming in the ancient near East. Nature *536*, 419–424. https://doi.org/10.1038/nature19310.

12. Damgaard, PdB., Marchi, N., Rasmussen, S., Peyrot, M., Renaud, G., Korneliussen, T., Moreno-Mayar, J.V., Pedersen, M.W., Goldberg, A., Usmanova, E., et al. (2018). 137 Ancient human genomes from across the Eurasian steppes. Nature *557*, 369–374. https://doi.org/10.1038/s41586-018-0094-2.

13. Clemente, F., Unterländer, M., Dolgova, O., Amorim, C.E.G., Coroado-Santos, F., Neuenschwander, S., Ganiatsou, E., Cruz Dávalos, D.I., Anchieri, L., Michaud, F., et al. (2021). The genomic history of the Aegean palatial civilizations. Cell *184*, 2565–2586.e21. https://doi.org/10.1016/j.cell.2021.03.039.

14. Feldman, M., Fernández-Domínguez, E., Reynolds, L., Baird, D., Pearson, J., Hershkovitz, I., May, H., Goring-Morris, N., Benz, M., Gresky, J., et al. (2019). Late Pleistocene human genome suggests a local origin for the first farmers of central Anatolia. Nat. Commun. *10*, 1218. https://doi.org/10.1038/s41467-019-09209-7.

15. Haber, M., Doumet-Serhal, C., Scheib, C., Xue, Y., Danecek, P., Mezzavilla, M., Youhanna, S., Martiniano, R., Prado-Martinez, J., Szpak, M., et al. (2017). Continuity and admixture in the last five millennia of Levantine history from ancient Canaanite and present-day Lebanese genome sequences. Am. J. Hum. Genet. *101*, 274–282. https://doi.org/10.1016/j.ajhg.2017.06.013.

16. Haber, M., Nassar, J., Almarri, M.A., Saupe, T., Saag, L., Griffith, S.J., Doumet-Serhal, C., Chanteau, J., Saghieh-Beydoun, M., Xue, Y., et al. (2020). A genetic history of the Near East from an aDNA time course sampling eight points in the past 4,000 years. Am. J. Hum. Genet. *107*, 149–157. https://doi.org/10.1016/j.ajhg.2020.05.008.

17. Kılınç, G.M., Koptekin, D., Atakuman, Ç., Sümer, A.P., Dönertaş, H.M., Yaka, R., Bilgin, C.C., Büyükkarakaya, A.M., Baird, D., Altınışık, E., et al. (2017). Archaeogenomic analysis of the first steps of Neolithization in Anatolia and the Aegean. Proc. Biol. Sci. *284*, 20172064. https://doi.org/10.1098/rspb.2017.2064.

18. Lazaridis, I., Mittnik, A., Patterson, N., Mallick, S., Rohland, N., Pfrengle, S., Furtwängler, A., Peltzer, A., Posth, C., Vasilakis, A., et al. (2017). Genetic origins of the Minoans and Mycenaeans. Nature *548*, 214–218. https://doi.org/10.1038/nature23310.

19. Narasimhan, V.M., Patterson, N., Moorjani, P., Rohland, N., Bernardos, R., Mallick, S., Lazaridis, I., Nakatsuka, N., Olalde, I., Lipson, M., et al. (2019). The formation of human populations in South and Central Asia. Science *365*, eaat7487. https://doi.org/10.1126/science.aat7487.

20. Skourtanioti, E., Erdal, Y.S., Frangipane, M., Balossi Restelli, F., Yener, K.A., Pinnock, F., Matthiae, P., Özbal, R., Schoop, U.D., Guliyev, F., et al. (2020). Genomic history of Neolithic to Bronze Age Anatolia, Northern Levant, and Southern Caucasus. Cell *181*, 1158–1175.e28. https://doi.org/10.1016/j.cell.2020.04.044.

21. Wang, C.-C., Reinhold, S., Kalmykov, A., Wissgott, A., Brandt, G., Jeong, C., Cheronet, O., Ferry, M., Harney, E., Keating, D., et al. (2019). Ancient human genome-wide data from a 3000-year interval in the Caucasus corresponds with eco-geographic regions. Nat. Commun. *10*, 590. https://doi.org/10.1038/s41467-018-08220-8.

22. Yaka, R., Mapelli, I., Kaptan, D., Doğu, A., Chyleński, M., Erdal, Ö.D., Koptekin, D., Vural, K.B., Bayliss, A., Mazzucato, C., et al. (2021). Variable kinship patterns in Neolithic Anatolia revealed by ancient genomes. Curr. Biol. *31*, 2455–2468.e18. https://doi.org/10.1016/j.cub.2021.03.050.

23. Mehrjoo, Z., Fattahi, Z., Beheshtian, M., Mohseni, M., Poustchi, H., Ardalani, F., Jalalvand, K., Arzhangi, S., Mohammadi, Z., Khoshbakht, S., et al. (2019). Distinct genetic variation and heterogeneity of the Iranian population. PLoS Genet. *15*, e1008385. https://doi.org/10.1371/journal.pgen.1008385.

24. Agranat-Tamir, L., Waldman, S., Martin, M.A.S., Gokhman, D., Mishol, N., Eshel, T., Cheronet, O., Rohland, N., Mallick, S., Adamski, N., et al. (2020). The genomic history of the Bronze Age Southern Levant. Cell *181*, 1146–1157.e11. https://doi.org/10.1016/j.cell.2020.04.024.

25. Haber, M., Mezzavilla, M., Xue, Y., and Tyler-Smith, C. (2016). Ancient DNA and the rewriting of human history: be sparing with Occam's razor. Genome Biol. *17*, 1. https://doi.org/10.1186/s13059-015-0866-z.

26. Feldman, M., Master, D.M., Bianco, R.A., Burri, M., Stockhammer, P.W., Mittnik, A., Aja, A.J., Jeong, C., and Krause, J. (2019). Ancient DNA sheds light on the genetic origins of early Iron Age Philistines. Sci. Adv. *5*, eaax0061. https://doi.org/10.1126/sciadv.aax0061.

27. Hughey, J.R., Paschou, P., Drineas, P., Mastropaolo, D., Lotakis, D.M., Navas, P.A., Michalodimitrakis, M., Stamatoyannopoulos, J.A., and Stamatoyannopoulos, G. (2013). A European population in Minoan Bronze Age Crete. Nat. Commun. *4*, 1861. https://doi.org/10.1038/ncomms2871.

28. Margaryan, A., Derenko, M., Hovhannisyan, H., Malyarchuk, B., Heller, R., Khachatryan, Z., Avetisyan, P., Badalyan, R., Bobokhyan, A., Melikyan, V., et al. (2017). Eight millennia of matrilineal genetic continuity in the South Caucasus. Curr. Biol. *27*, 2023–2028.e7. https://doi.org/10.1016/j.cub.2017.05.087.

29. Yaka, R., Birand, A., Yılmaz, Y., Caner, C., Açan, S.C., Gündüzalp, S., Parvizi, P., Erim Özdoğan, A., Togan, İ., and Somel, M. (2018). Archaeogenetics of Late Iron Age Çemialo Sırtı, Batman: investigating maternal genetic continuity in north Mesopotamia since the Neolithic. Am. J. Phys. Anthropol. *166*, 196–207. https://doi.org/10.1002/ajpa.23423.

30. 1000 Genomes Project Consortium, Auton, A., Brooks, L.D., Durbin, R.M., Garrison, E.P., Kang, H.M., Korbel, J.O., Marchini, J.L., McCarthy, S., McVean, G.A., et al. (2015). A global reference for human genetic variation. Nature *526*, 68–74. https://doi.org/10.1038/nature15393.

31. Mathieson, I., Lazaridis, I., Rohland, N., Mallick, S., Patterson, N., Roodenberg, S.A., Harney, E., Stewardson, K., Fernandes, D., Novak, M., et al. (2015). Genome-wide patterns of selection in 230 ancient Eurasians. Nature *528*, 499–503. https://doi.org/10.1038/nature16152.

32. Patterson, N., Moorjani, P., Luo, Y., Mallick, S., Rohland, N., Zhan, Y., Genschoreck, T., Webster, T., and Reich, D. (2012). Ancient admixture in human history. Genetics *192*, 1065–1093. https://doi.org/10.1534/genetics.112.145037.

33. Haak, W., Lazaridis, I., Patterson, N., Rohland, N., Mallick, S., Llamas, B., Brandt, G., Nordenfelt, S., Harney, E., Stewardson, K., et al. (2015). Massive migration from the steppe was a source for Indo-European languages in Europe. Nature *522*, 207–211. https://doi.org/10.1038/nature14317.

34. Harney, É., Patterson, N., Reich, D., and Wakeley, J. (2021). Assessing the performance of qpAdm: a statistical tool for studying population admixture. Genetics *217*, iyaa045. https://doi.org/10.1093/GENETICS/IYAA045.

35. Altınışık, N.E., Kazancı, D.D., Aydoğan, A., Gemici, H.C., Erdal, Ö.D., Sarıaltun, S., Vural, K.B., Koptekin, D., Gürün, K., Sağlıcan, E., et al. (2022). A genomic snapshot of demographic and cultural dynamism in Upper Mesopotamia during the Neolithic Transition. Sci. Adv. *8*, eabo3609. https://doi.org/10.1126/sciadv.abo3609.

36. Chintalapati, M., Patterson, N., and Moorjani, P. (2022). The spatiotemporal patterns of major human admixture events during the European Holocene. eLife *11*, e77625. https://doi.org/10.7554/eLife.77625.

37. de Barros Damgaard, P., Martiniano, R., Kamm, J., Moreno-Mayar, J.V., Kroonen, G., Peyrot, M., Barjamovic, G., Rasmussen, S., Zacho, C., Baimukhanov, N., et al. (2018). The first horse herders and the impact of early Bronze Age steppe expansions into Asia. Science *360*, eaar7711. https://doi.org/10.1126/science.aar7711.

38. Lazaridis, I., Alpaslan-Roodenberg, S., Acar, A., Açıkkol, A., Agelarakis, A., Aghikyan, L., Akyüz, U., Andreeva, D., Andrijašević, G., Antonović, D., et al. (2022). The genetic history of the Southern Arc: a bridge between

West Asia and Europe. Science *377*, eabm4247. https://doi.org/10.1126/science.abm4247.

39. Kars, M.E., Başak, A.N., Onat, O.E., Bilguvar, K., Choi, J., Itan, Y., Çaglar, C., Palvadeau, R., Casanova, J.L., Cooper, D.N., et al. (2021). The genetic structure of the Turkish population reveals high levels of variation and admixture. Proc. Natl. Acad. Sci. USA *118*. e2026076118. https://doi.org/10.1073/pnas.2026076118.
40. Hofmanová, Z., Kreutzer, S., Hellenthal, G., Sell, C., Diekmann, Y., Díez-Del-Molino, D., Van Dorp, L., López, S., Kousathanas, A., Link, V., et al. (2016). Early farmers from across Europe directly descended from Neolithic Aegeans. Proc. Natl. Acad. Sci. USA *113*, 6886–6891. https://doi.org/10.1073/pnas.1523951113.
41. Orsaria, F. (1995). Shah Tepe: a new approach to an old excavation. Riv. Stud. Orient. *69*, 481–495.
42. Thornton, C.P. (2013). The Bronze Age in Northeastern Iran. In The Oxford Handbook of Ancient Iran, D.T. Potts, ed. (Oxford University Press). https://doi.org/10.1093/oxfordhb/9780199733309.013.0034.
43. Rasia, P.A., Bitadze, L., Rova, E., and Bertoldi, F. (2021). Bronze Age burials from Doghlauri (Georgia). Preliminary analysis of human remains reveals a change in burial customs. J. Archaeol. Sci. Rep. *38*, 103048. https://doi.org/10.1016/j.jasrep.2021.103048.
44. Haber, M., Doumet-Serhal, C., Scheib, C.L., Xue, Y., Mikulski, R., Martiniano, R., Fischer-Genz, B., Schutkowski, H., Kivisild, T., and Tyler-Smith, C. (2019). A transient pulse of genetic admixture from the crusaders in the Near East identified from ancient genome sequences. Am. J. Hum. Genet. *104*, 977–984. https://doi.org/10.1016/j.ajhg.2019.03.015.
45. Skoglund, P., Malmström, H., Omrak, A., Raghavan, M., Valdiosera, C., Günther, T., Hall, P., Tambets, K., Parik, J., Sjögren, K.-G., et al. (2014). Genomic diversity and admixture differs for Stone-Age Scandinavian foragers and farmers. Science *344*, 747–750. https://doi.org/10.1126/science.1253448.
46. Ringbauer, H., Novembre, J., and Steinrücken, M. (2021). Parental relatedness through time revealed by runs of homozygosity in ancient DNA. Nat. Commun. *12*, 5425. https://doi.org/10.1038/s41467-021-25289-w.
47. Ceballos, F.C., Joshi, P.K., Clark, D.W., Ramsay, M., and Wilson, J.F. (2018). Runs of homozygosity: windows into population history and trait architecture. Nat. Rev. Genet. *19*, 220–234. https://doi.org/10.1038/nrg.2017.109.
48. Whitlock, M.C., and McCauley, D.E. (1999). Indirect measures of gene flow and migration: FST not equal to 1/(4Nm + 1). Heredity *82*, 117–125. https://doi.org/10.1038/sj.hdy.6884960.
49. Harris, A.M., and DeGiorgio, M. (2017). Admixture and ancestry inference from ancient and modern samples through measures of population genetic drift. Hum. Biol. *89*, 21–46. https://doi.org/10.13110/humanbiology.89.1.02.
50. Peter, B.M. (2016). Admixture, population structure, and F-statistics. Genetics *202*, 1485–1501. https://doi.org/10.1534/genetics.115.183913.
51. Gavashelishvili, A., Yanchukov, A., Tarkhnishvili, D., Murtskhvaladze, M., Akhvlediani, I., and Kazancı, C. (2021). Landscape genetics and the genetic legacy of Upper Paleolithic and Mesolithic hunter-gatherers in the modern Caucasus. Sci. Rep. *11*, 17985. https://doi.org/10.1038/s41598-021-97519-6.
52. Goldberg, A., Günther, T., Rosenberg, N.A., and Jakobsson, M. (2017). Ancient X chromosomes reveal contrasting sex bias in Neolithic and Bronze Age Eurasian migrations. Proc. Natl. Acad. Sci. USA *114*, 2657–2662. https://doi.org/10.1073/pnas.1616392114.
53. Olalde, I., Mallick, S., Patterson, N., Rohland, N., Villalba-Mouco, V., Silva, M., Dulias, K., Edwards, C.J., Gandini, F., Pala, M., et al. (2019). The genomic history of the Iberian Peninsula over the past 8000 years. Science *363*, 1230–1234. https://doi.org/10.1126/science.aav4040.
54. Jun, K.-H. (2013). Demographics and migration: an overview. In The Encyclopedia of Global Human Migration, I. Ness, ed. (Wiley-Blackwell). https://doi.org/10.1002/9781444351071.wbeghm16.
55. Loog, L., Mirazón Lahr, M., Kovacevic, M., Manica, A., Eriksson, A., and Thomas, M.G. (2017). Estimating mobility using sparse data: application to human genetic variation. Proc. Natl. Acad. Sci. USA *114*, 12213–12218. https://doi.org/10.1073/pnas.1703642114.
56. Palmisano, A., Lawrence, D., de Gruchy, M.W., Bevan, A., and Shennan, S. (2021). Holocene regional population dynamics and climatic trends in the Near East: a first comparison using archaeo-demographic proxies. Quat. Sci. Rev. *252*, 106739. https://doi.org/10.1016/j.quascirev.2020.106739.
57. Meyer, M., and Kircher, M. (2010). Illumina sequencing library preparation for highly multiplexed target capture and sequencing. Cold Spring Harb. Protoc. *2010*. pdb.prot5448. https://doi.org/10.1101/pdb.prot5448.
58. Psonis, N., Vassou, D., and Kafetzopoulos, D. (2021). Testing a series of modifications on genomic library preparation methods for ancient or degraded DNA. Anal. Biochem. *623*, 114193. https://doi.org/10.1016/j.ab.2021.114193.
59. Schubert, M., Lindgreen, S., and Orlando, L. (2016). AdapterRemoval v2: rapid adapter trimming, identification, and read merging. BMC Res. Notes *9*, 88. https://doi.org/10.1186/s13104-016-1900-2.
60. Li, H., and Durbin, R. (2009). Fast and accurate short read alignment with Burrows-Wheeler transform. Bioinformatics *25*, 1754–1760. https://doi.org/10.1093/bioinformatics/btp324.
61. Kircher, M. (2012). Analysis of high-throughput ancient DNA sequencing data. Methods Mol. Biol. *840*, 197–228. https://doi.org/10.1007/978-1-61779-516-9_23.
62. Li, H., Handsaker, B., Wysoker, A., Fennell, T., Ruan, J., Homer, N., Marth, G., Abecasis, G., and Durbin, R.; 1000 Genome Project Data Processing Subgroup (2009). The Sequence Alignment/Map format and SAMtools. Bioinformatics *25*, 2078–2079. https://doi.org/10.1093/bioinformatics/btp352.
63. Korneliussen, T.S., Albrechtsen, A., and Nielsen, R. (2014). ANGSD: analysis of next generation sequencing data. BMC Bioinformatics *15*, 356. https://doi.org/10.1186/s12859-014-0356-4.
64. Weissensteiner, H., Pacher, D., Kloss-Brandstätter, A., Forer, L., Specht, G., Bandelt, H.J., Kronenberg, F., Salas, A., and Schönherr, S. (2016). HaploGrep 2: mitochondrial haplogroup classification in the era of high-throughput sequencing. Nucleic Acids Res. *44*, W58–W63. https://doi.org/10.1093/nar/gkw233.
65. Patterson, N., Price, A.L., and Reich, D. (2006). Population structure and eigenanalysis. PLoS Genet. *2*, e190. https://doi.org/10.1371/journal.pgen.0020190.
66. Monroy Kuhn, J.M.M., Jakobsson, M., and Günther, T. (2018). Estimating genetic kin relationships in prehistoric populations. PLoS One *13*, e0195491. https://doi.org/10.1371/journal.pone.0195491.
67. Alexander, D.H., Novembre, J., and Lange, K. (2009). Fast model-based estimation of ancestry in unrelated individuals. Genome Res. *19*, 1655–1664. https://doi.org/10.1101/gr.094052.109.
68. Chang, C.C., Chow, C.C., Tellier, L.C., Vattikuti, S., Purcell, S.M., and Lee, J.J. (2015). Second-generation PLINK: rising to the challenge of larger and richer datasets. GigaScience *4*, 7. https://doi.org/10.1186/s13742-015-0047-8.
69. Quinlan, A.R., and Hall, I.M. (2010). BEDTools: a flexible suite of utilities for comparing genomic features. Bioinform. Oxf. Engl. *26*, 841–842. https://doi.org/10.1093/bioinformatics/btq033.
70. Fu, Q., Mittnik, A., Johnson, P.L.F., Bos, K., Lari, M., Bollongino, R., Sun, C., Giemsch, L., Schmitz, R., Burger, J., et al. (2013). A revised timescale for human evolution based on ancient mitochondrial genomes. Curr. Biol. *23*, 553–559. https://doi.org/10.1016/j.cub.2013.02.044.
71. Li, H. (2011). A statistical framework for SNP calling, mutation discovery, association mapping and population genetical parameter estimation from sequencing data. Bioinformatics *27*, 2987–2993. https://doi.org/10.1093/bioinformatics/btr509.

72. van Oven, M., and Kayser, M. (2009). Updated comprehensive phylogenetic tree of global human mitochondrial DNA variation. Hum. Mutat. *30*, E386–E394. https://doi.org/10.1002/humu.20921.
73. Poznik, G.D. (2016). Identifying Y-chromosome haplogroups in arbitrarily large samples of sequenced or genotyped men. Preprint at bioRxiv. https://doi.org/10.1101/088716.
74. Excoffier, L., and Lischer, H.E.L. (2010). Arlequin suite ver 3.5: a new series of programs to perform population genetics analyses under Linux and Windows. Mol. Ecol. Resour. *10*, 564–567. https://doi.org/10.1111/j.1755-0998.2010.02847.x.
75. Jun, G., Wing, M.K., Abecasis, G.R., and Kang, H.M. (2015). An efficient and scalable analysis framework for variant extraction and refinement from population-scale DNA sequence data. Genome Res. *25*, 918–925. https://doi.org/10.1101/gr.176552.114.
76. Kelleher, J., Etheridge, A.M., and McVean, G. (2016). Efficient coalescent simulation and genealogical analysis for large sample sizes. PLoS Comput. Biol. *12*, e1004842. https://doi.org/10.1371/journal.pcbi.1004842.
77. Miles, A., pyup.io bot, Murillo, R., Ralph, P., Harding, N., Pisupati, R., Rae, S., and Millar, T. (2021). cggh/scikit-allel: v1.3.3. https://doi.org/10.5281/zenodo.4759368.
78. Özbaşaran, M. (1999). Musular: a general assessment on a new Neolithic site in Central Anatolia. Neolit Turk. Cradle Civiliz, 147–155.
79. Özbaşaran, M. (2000). The Neolithic site of Musular, Central Anatolia. Anatolica *26*, 129–151. https://doi.org/10.2143/ANA.26.0.2015496.
80. Özbaşaran, M., Duru, G., Kayacan, N., Erdoğu, B., and Buitenhuis, H. (2007). Musular 1996–2004: Genel Değerlendirme. Türkiye'de neolit. Dönem, 273–283.
81. Esin, U., and Harmankaya, S. (1999). Aşıklı. Neolit Turk. Cradle Civilis New Discov. *2*, 115–132.
82. Esin, U., and Harmankaya, S. (2007). Aşıklı Höyük. Anadolu'da Uygarlığın Doğuşu Avrupaya Yayılımı Türkiye'de neolit. Dönem Yeni Kazılar Yeni Bulgular Istanb. Turk. Arkeol. Sanat Yayın, 255–272.
83. Kayacan, N. (2003). Chipped stone industry of the Neolithic site of Musular (Cappadocia): preliminary results. Anatolia Antiq Eski Anadolu *11*, 1–10. https://doi.org/10.3406/anata.2003.991.
84. Kayacan, N., and Özbaşaran, M. (2007). The choice of obsidian and its use at Musular, central Anatolia. Tech. Syst. East. PPN Commun. Éditions APDCA Antibes, 229–233.
85. Astruc, L., Kayacan, N., and Özbaşaran, M. (2008). Technical activities held at Musular (VIIIth Millennium Anatolia): a preliminary use-wear analysis of lithic tools. Arkeometri Sonuçları Toplantısı *23*, 165–172.
86. Duru, G., and Özbaşaran, M. (2005). A 'non domestic' site in Central Anatolia. Anatolia Antiq Eski Anadolu *13*, 15–28.
87. Günel, S. (2014). New contributions regarding prehistoric cultures in the meander region: Çine-Tepecik. In Western Anatolia before Troy. Proto-Urbanisation in the 4th Millennium BC?, B. Horejs, and M. Mehofer, eds. (Austrian Academy of Sciences Press), pp. 83–104.
88. Günel, S. (2015). Çine−Tepecik: new contributions on late Bronze Age cultures in western Anatolia. In Nostoi: Indigenous Culture, Migration and Integration in the Aegean Islands and Western Anatolia during the Late Bronze and Early Iron Age (Koç University Press), pp. 627–646.
89. Günel, S. (2021). Çine-Tepecik'in Kültürel Gelişiminde Kentleşme Sürecini Anlamak. Anadolu Araştırmaları, 117–144. https://doi.org/10.26650/anar.2021.24.945531.
90. Günel, S. (2010). Mycenaean cultural impact on the Çine (Marsyas) plain, southwest Anatolia: the evidence from Çine-Tepecik. Anatol. Stud. *60*, 25–49. https://doi.org/10.1017/S0066154600001009.
91. Günel, S. (2019). Batı Anadolu-Ege İlişkilerinin Çine-Tepecik Merkezine Yansımaları, Anadolu, Kültürlerin Bağlantısı. In Başlangıcından Roma Dönemi Sonuna Kadar Eski Yakın Doğuda Ticaret ve Bölgelerarası İlişkiler / Connecting Cultures. Trade and Interconnections in the Ancient Near East from the Beginning until the End of the Roman Period, V. Şahoğlu, M. Şevketoğlu, and Y.H. Erbil, eds., pp. 187–204.
92. Lovejoy, C.O., Meindl, R.S., Pryzbeck, T.R., and Mensforth, R.P. (1985). Chronological metamorphosis of the auricular surface of the ilium: a new method for the determination of adult skeletal age at death. Am. J. Phys. Anthropol. *68*, 15–28. https://doi.org/10.1002/ajpa.1330680103.
93. Ubelaker, D.H., and Grant, L.G. (1989). Human skeletal remains: preservation or reburial? Am. J. Phys. Anthropol. *32*, 249–287. https://doi.org/10.1002/ajpa.1330320511.
94. Rose, C.B. (2017). Fieldwork at Phrygian Gordion, 2013–2015. Am. J. Archaeol. *121*, 135–178. https://doi.org/10.3764/aja.121.1.0135.
95. Kohler, E.L. (1995). The Gordion Excavations, 1950–1973, Final Reports, Volume II: The Lesser Phrygian Tumuli, Part 1: The Inhumations (UPenn Museum of Archaeology).
96. Liebhart, R., Darbyshire, G., Erder, E., and Marsh, B. (2016). A fresh look at the Tumuli of Gordion. In Tumulus as Sema (De Gruyter), pp. 627–636.
97. Rose, C.B. (2012). The Archaeology of Phrygian Gordion, Royal City of Midas: Gordion Special Studies 7 (University of Pennsylvania Press).
98. Rose, C.B., and Darbyshire, G. (2011). The New Chronology of Iron Age Gordion. Gordion Special Studies VI (University of Pennsylvania Press).
99. Voigt, M.M. (2013). Gordion as citadel and city. In Cities and Citadels in Turkey: from the Iron Age to the Seljuks, S. Redford, and N. Ergin, eds. (Peeters), pp. 161–228.
100. Schachner, A. (2011). Hattuscha. Die Hauptstadt der Hethiter: auf der Suche nach dem Sagenhaften Großreich der Hethiter (Beck C. H.).
101. Schachner, A. (2019). Die Ausgrabungen in Boğazköy-Ḫattuša 2018. Archäol. Anz. 1–146. https://doi.org/10.34780/1vvt-j762.
102. Kühne, H. (1969). Die Bestattungen der hellenistischen bis spätkaiserzeitlichen Periode. In Funde aus den Grabungen 1967 und 1968 (Boğazköy IV), K. Bittel, ed. (Gebr Mann Verlag), pp. 35–45.
103. Schachner, A. (2010). Die Ausgrabungen in Boğazköy-Ḫattuša 2009. Archäol. Anz. 161–221.
104. Manolis, S., Aiello, L., Henesse, R., and Kyparissi-Apostolika, N. (2006). The Middle Paleolithic footprints from Theopetra cave. Proceedings of the International Conference. Theopetra Cave, Twelve years of excavation and Research, pp. 81–95.
105. Facorellis, Y., Kyparissi-Apostolika, N., and Maniatis, Y. (2001). The cave of Theopetra, Kalambaka: radiocarbon evidence for 50,000 years of human presence. Radiocarbon *43*, 1029–1048. https://doi.org/10.1017/S0033822200041692.
106. Facorellis, G., and Maniatis, J. (2006). Evidence of 50,000yrs. of human activity in the cave of Theopetra by C14. Proceedings of the International Conference. Theopetra Cave, Twelve years of excavation and Research, pp. 53–69.
107. Evison, M., Kyparissi-Apostolika, N., Stravopodi, E., Fieller, N., and Smilie, D. (2006). An ancient HLA type from a Palaeolithic skeleton from Theopetra Cave, Greece. Proceedings of the International Conference. Theopetra Cave, Twelve years of excavation and Research, pp. 109–119.
108. Manolis, S., and Stravopodi, E. (2003). An assessment of the human skeletal remains in the Mesolithic deposits of Theopetra cave: a case study. In Greek Mesolithic: Problems and Perspectives, N. Galanidou, and C. Perles, eds. (British School at Athens), pp. 207–217.
109. Karkanas, P., White, D., Lane, C.S., Stringer, C., Davies, W., Cullen, V.L., Smith, V.C., Ntinou, M., Tsartsidou, G., and Kyparissi-Apostolika, N. (2015). Tephra correlations and climatic events between the MIS6/5 transition and the beginning of MIS3 in Theopetra Cave, central Greece. Quat. Sci. Rev. *118*, 170–181.
110. Stravopodi, E., and Manolis, S. (2006). The bioarchaeological profile of the anthropological finds of Theopetra cave: a pilot study in Greek peninsula. Proceedings of the International Conference. Theopetra Cave, Twelve years of excavation and Research, pp. 95–109.
111. Stravopodi, E. (2022). The anthropological material from Theopetra cave. In Neolithic Theopetra, N. Kyparissi-Apostolika, ed. (TAP, Ministry of Culture and Sports).
112. Kyparissi-Apostolika, N. (2003). The Mesolithic in Theopetra Cave: new data on a debated period of Greek prehistory. In Greek Mesolithic:

Problems and Perspectives, N. Galanidou, and C. Perles, eds. (British School at Athens), pp. 189–198.

113. Kyparissi-Apostolika, N. (2006). The excavation in Theopetra cave. Proceedings of the International Conference. Theopetra Cave, Twelve years of excavation and Research, pp. 15–17.

114. Sampson, A.A. (2016). The Sarakenos Cave at Akraephnion, Boeotia, Greece, Volume II: The Early Neolithic, the Mesolithic and the Final Paleoliothic (University of the Aegean, Polish Academy of Arts and Sciences).

115. Allen, H. (1997). The environmental conditions of the Kopais basin, Boeotia, during the post-glacial with special reference to the Mycenaean period. In Recent Developments in the History and Archaeology of Central Greece, J. Bintliff, ed. (Oxford), pp. 39–58.

116. Sampson, A. (2000). The Sarakenos Cave and the occupation of caves in Kopais basin. Proceedings of the 3rd International Conference of Boeotian Studies.

117. Sampson, A. (2014). The Sarakenos Cave at Akraefnion, Boeotia, Contribution to the Prehistory of Central Greece Monograph*6* (University of the Aegean; Polish Academy of the Arts and Sciences).

118. Katsarou, S., and Sampson, A. (2013). Perspectives of symbolism and ritualism for the Late Neolithic communities at Sarakenos Cave. In Stable Places and Changing Perceptions: Cave Archaeology in Greece and Adjacent Areas Cave Archaeology in Greece. BAR International Series, F. Mavridis, and J. Jensen, eds. (Oxford), pp. 142–152.

119. Sampson, A.A. (2008). The Sarakenos Cave at Akraephnion, Boeotia, Greece: the Neolithic and the Bronze Age: Pottery Analysis, Cave Occupation Patterns and Populations Movements in Central and Southern Greece. The Research from 1994–2000, Volume 1 (University of the Aegean).

120. Fossey, J.M. (1986). The Ancient Topography of Eastern Phokis Illustrated Edition (Brill).

121. Betancourt, P.P. (2009). The Bronze Age Begins: the Ceramics Revolution of Early Minoan I and the New Forms of Wealth That Transformed Prehistoric Society (INSTAP Academic Press).

122. Schultz, M. (2001). Paleohistopathology of bone: a new approach to the study of ancient diseases. Am. J. Phys. Anthropol. *116*, 106–147. https://doi.org/10.1002/ajpa.10024.

123. Hatzipouliou-Kalliri, E. (1983). An early Helladic II tomb by lake Vouliagmeni, Perachora. Annu. Br. Sch. Athens *78*, 369–375.

124. Bukhrashvili, P., Blocher, F., Tskvitinidze, Z., Davitashvili, S., and Fassbinder, J. (2020). Ausgrabungen in Nazarlebi, Kachetien (Georgien) 2019 *152*, 125–154.

125. Davitashvili, S., Bukhrashvili, P., and Blocher, F. (2021). Nazarlebi, ein 3000 Jahre altes Heiligtum in Kachetien (Ost-Georgien). Altert *66*, 189–218.

126. Kasimova, R.M. (1960). Antropologicheskoe issledovanie cherepov iz Mingechaura (v svyazi s izucheniem etnogeneza azerbaydzhanskogo naroda [Anthropological study of skulls from Mingachevir (in connection with the study of the ethnogenesis of the Azerbaijani people)], p. 136.

127. Arne, T.A.J. (1945). Excavations at Shah tepé, Iran (Elanders boktryckeri aktiebolag).

128. Fürst, C.M., and Amschler, J.W. (1939). The Skeletal Material Collected During the Excavations of Dr. T.J. Arne in Shah Tepé at Astrabad-Gorgan in Iran (Bokförlags aktiebolaget Thule).

129. Dabney, J., Knapp, M., Glocke, I., Gansauge, M.T., Weihmann, A., Nickel, B., Valdiosera, C., García, N., Pääbo, S., Arsuaga, J.-L., and Meyer, M. (2013). Complete mitochondrial genome sequence of a Middle Pleistocene cave bear reconstructed from ultrashort DNA fragments. Proc. Natl. Acad. Sci. USA *110*, 15758–15763. https://doi.org/10.1073/pnas.1314445110.

130. Schubert, M., Ginolhac, A., Lindgreen, S., Thompson, J.F., Al-Rasheid, K.A.S., Willerslev, E., Krogh, A., and Orlando, L. (2012). Improving ancient DNA read mapping against modern reference genomes. BMC Genomics *13*, 178. https://doi.org/10.1186/1471-2164-13-178.

131. Krzewińska, M., Kılınç, G.M., Juras, A., Koptekin, D., Chyleński, M., Nikitin, A.G., Shcherbakov, N., Shuteleva, I., Leonova, T., Kraeva, L., et al. (2018). Ancient genomes suggest the eastern Pontic-Caspian steppe as the source of western Iron Age nomads. Sci. Adv. *4*, eaat4457. https://doi.org/10.1126/sciadv.aat4457.

132. Fulton, T.L., and Shapiro, B. (2019). Setting up an ancient DNA laboratory. In Ancient DNA, B. Shapiro, A. Barlow, P. Heintzman, M. Hofreiter, J. Paijmans, and A. Soares, eds. (Humana Press), pp. 1–13.

133. Allentoft, M.E., Heller, R., Holdaway, R.N., and Bunce, M. (2015). Ancient DNA microsatellite analyses of the extinct New Zealand giant moa (Dinornis robustus) identify relatives within a single fossil site. Heredity *115*, 481–487. https://doi.org/10.1038/hdy.2015.48.

134. Psonis, N., de Carvalho, C.N., Figueiredo, S., Tabakaki, E., Vassou, D., Poulakakis, N., and Kafetzopoulos, D. (2020). Molecular identification and geographic origin of a post-medieval elephant finding from southwestern Portugal using high-throughput sequencing. Sci. Rep. *10*, 19252. https://doi.org/10.1038/s41598-020-75323-y.

135. Psonis, N., Vardinoyannis, K., and Poulakakis, N. (2022). High-throughput degraded DNA sequencing of subfossil shells of a critically endangered stenoendemic land snail in the Aegean. Mol. Phylogenet. Evol. *175*, 107561. https://doi.org/10.1016/j.ympev.2022.107561.

136. Psonis, N., Vassou, D., Nicolaou, L., Roussiakis, S., Iliopoulos, G., Poulakakis, N., and Sfenthourakis, S. (2021). Mitochondrial sequences of the extinct Cypriot pygmy hippopotamus confirm its phylogenetic placement. Zool. J. Linn. Soc. *196*, 979–989. https://doi.org/10.1093/zoolinnean/zlab089.

137. Carøe, C., Gopalakrishnan, S., Vinner, L., Mak, S.S.T., Sinding, M.H.S., Samaniego, J.A., Wales, N., Sicheritz-Pontén, T., and Gilbert, M.T.P. (2018). Single-tube library preparation for degraded DNA. Methods Ecol. Evol. *9*, 410–419. https://doi.org/10.1111/2041-210X.12871.

138. Gansauge, M.-T., Gerber, T., Glocke, I., Korlevic, P., Lippik, L., Nagel, S., Riehl, L.M., Schmidt, A., and Meyer, M. (2017). Single-stranded DNA library preparation from highly degraded DNA using T4 DNA ligase. Nucleic Acids Res. *45*, e79. https://doi.org/10.1093/nar/gkx033.

139. Reimer, P.J., Austin, W.E.N., Bard, E., Bayliss, A., Blackwell, P.G., Bronk Ramsey, C.B., Butzin, M., Cheng, H., Edwards, R.L., Friedrich, M., et al. (2020). The IntCal20 Northern Hemisphere radiocarbon age calibration curve (0–55 cal kBP). Radiocarbon *62*, 725–757. https://doi.org/10.1017/RDC.2020.41.

140. Skoglund, P., Storå, J., Götherström, A., and Jakobsson, M. (2013). Accurate sex identification of ancient human remains using DNA shotgun sequencing. J. Archaeol. Sci. *40*, 4477–4482. https://doi.org/10.1016/j.jas.2013.07.004.

141. Benjamini, Y., and Hochberg, Y. (1995). Controlling the false discovery rate: a practical and powerful approach to multiple testing. J. R. Stat. Soc. B *57*, 289–300. https://doi.org/10.1111/j.2517-6161.1995.tb02031.x.

142. Clark, A.G., Hubisz, M.J., Bustamante, C.D., Williamson, S.H., and Nielsen, R. (2005). Ascertainment bias in studies of human genome-wide polymorphism. Genome Res. *15*, 1496–1502. https://doi.org/10.1101/gr.4107905.

143. Bergström, A., McCarthy, S.A., Hui, R., Almarri, M.A., Ayub, Q., Danecek, P., Chen, Y., Felkel, S., Hallast, P., Kamm, J., et al. (2020). Insights into human genetic variation and population history from 929 diverse genomes. Science *367*, eaay5012. https://doi.org/10.1126/science.aay5012.

144. Tucci, S., Vohr, S.H., McCoy, R.C., Vernot, B., Robinson, M.R., Barbieri, C., Nelson, B.J., Fu, W., Purnomo, G.A., Sudoyo, H., et al. (2018). Evolutionary history and adaptation of a human pygmy population of Flores Island, Indonesia. Science *361*, 511–516. https://doi.org/10.1126/science.aar8486.

145. Mallick, S., Li, H., Lipson, M., Mathieson, I., Gymrek, M., Racimo, F., Zhao, M., Chennagiri, N., Nordenfelt, S., Tandon, A., et al. (2016). The Simons Genome Diversity Project: 300 genomes from 142 diverse populations. Nature *538*, 201–206. https://doi.org/10.1038/nature18964.

146. Lazaridis, I., Patterson, N., Mittnik, A., Renaud, G., Mallick, S., Kirsanow, K., Sudmant, P.H., Schraiber, J.G., Castellano, S., Lipson, M., et al.

(2014). Ancient human genomes suggest three ancestral populations for present-day Europeans. Nature *513*, 409–413. https://doi.org/10.1038/nature13673.

147. Jeong, C., Balanovsky, O., Lukianova, E., Kahbatkyzy, N., Flegontov, P., Zaporozhchenko, V., Immel, A., Wang, C.-C., Ixan, O., Khussainova, E., et al. (2019). The genetic history of admixture across inner Eurasia. Nat. Ecol. Evol. *3*, 966–976. https://doi.org/10.1038/s41559-019-0878-2.

148. Omrak, A., Günther, T., Valdiosera, C., Svensson, E.M., Malmström, H., Kiesewetter, H., Aylward, W., Storå, J., Jakobsson, M., and Götherström, A. (2016). Genomic evidence establishes Anatolia as the source of the European Neolithic gene pool. Curr. Biol. *26*, 270–275. https://doi.org/10.1016/j.cub.2015.12.019.

149. Broushaki, F., Thomas, M.G., Link, V., López, S., van Dorp, L., Kirsanow, K., Hofmanová, Z., Diekmann, Y., Cassidy, L.M., Díez-del-Molino, D., et al. (2016). Early Neolithic genomes from the eastern Fertile Crescent. Science *353*, 499–503. https://doi.org/10.1126/science.aaf7943.

150. Allentoft, M.E., Sikora, M., Sjögren, K.G., Rasmussen, S., Rasmussen, M., Stenderup, J., Damgaard, P.B., Schroeder, H., Ahlström, T., Vinner, L., et al. (2015). Population genomics of Bronze Age Eurasia. Nature *522*, 167–172. https://doi.org/10.1038/nature14507.

151. Jones, E.R., Gonzalez-Fortes, G., Connell, S., Siska, V., Eriksson, A., Martiniano, R., McLaughlin, R.L., Gallego Llorente, M., Cassidy, L.M., Gamba, C., et al. (2015). Upper Palaeolithic genomes reveal deep roots of modern Eurasians. Nat. Commun. *6*, 8912. https://doi.org/10.1038/ncomms9912.

152. Fu, Q., Posth, C., Hajdinjak, M., Petr, M., Mallick, S., Fernandes, D., Furtwängler, A., Haak, W., Meyer, M., Mittnik, A., et al. (2016). The genetic history of Ice Age Europe. Nature *534*, 200–205. https://doi.org/10.1038/nature17993.

153. Mathieson, I., Alpaslan-Roodenberg, S., Posth, C., Szécsényi-Nagy, A., Rohland, N., Mallick, S., Olalde, I., Broomandkhoshbacht, N., Candilio, F., Cheronet, O., et al. (2018). The genomic history of southeastern Europe. Nature *555*, 197–203. https://doi.org/10.1038/nature25778.

154. Omori, T., and Nakamura, T. (2006). Radiocarbon dating of archaeological materials excavated at Kaman-Kalehöyük: initial report. Anatol. Archaeol. Stud. *15*, 263–268.

155. Fu, Q., Li, H., Moorjani, P., Jay, F., Slepchenko, S.M., Bondarev, A.A., Johnson, P.L.F., Aximu-Petri, A.A., Prüfer, K., de Filippo, C., et al. (2014). Genome sequence of a 45,000-year-old modern human from western Siberia. Nature *514*, 445–449. https://doi.org/10.1038/nature13810.

156. Kefi, R., Hechmi, M., Naouali, C., Jmel, H., Hsouna, S., Bouzaid, E., Abdelhak, S., Beraud-Colomb, E., and Stevanovitch, A. (2018). On the origin of Iberomaurusians: new data based on ancient mitochondrial DNA and phylogenetic analysis of Afalou and Taforalt populations. Mitochondrial DNA A DNA Mapp. Seq. Anal. *29*, 147–157. https://doi.org/10.1080/24701394.2016.1258406.

157. Wickham, H., Averick, M., Bryan, J., Chang, W., McGowan, L.D., François, R., Grolemund, G., Hayes, A., Henry, L., Hester, J., et al. (2019). Welcome to the tidyverse. J. Open Source Softw. *4*, 1686. https://doi.org/10.21105/joss.01686.

158. Wickham, H. (2011). The split-apply-combine strategy for data analysis. J. Stat. Softw. *40*, 1–29. https://doi.org/10.18637/jss.v040.i01.

159. Wickham, H. (2007). Reshaping data with the reshape package. J. Stat. Softw. *21*, 1–20. https://doi.org/10.18637/jss.v021.i12.

160. Conway, M. (2020). gsheet: download Google sheets using just the URL. R package version 0.4.5. https://CRAN.R-project.org/package=gsheet.

161. Wickham, H. (2016). ggplot2: Elegant Graphics for Data Analysis, Second Edition (Springer).

162. Wilke, C. (2020). ggtext: improved text rendering support for “ggplot2”. R package version 0.1.1. https://CRAN.R-project.org/package=ggtext.

163. Pedersen, T.L. (2022). ggforce: accelerating “ggplot2”. https://ggforce.data-imaginist.com. https://github.com/thomasp85/ggforce.

164. Kassambara, A. (2020). ggpubr: “ggplot2” based publication ready plots. R package version 0.4.0. https://CRAN.R-project.org/package=ggpubr.

165. Slowikowski, K., Schep, A., Hughes, S., Dang, T.K., Lukauskas, S., Irisson, J.-O., Kamvar, Z.N., Ryan, T., Christophe, D., Hiroaki, Y., et al. (2021). ggrepel: automatically position non-overlapping text labels with “ggplot2”. https://rdrr.io/cran/ggrepel/.

166. Becker, R.A., Wilks, A.R., Brownrigg, R., Minka, T.P., and Deckmyn, A. (2018). maps: draw geographical maps. R package version 3.3.0. https://CRAN.R-project.org/package=maps.

167. Hijmans, R.J., Etten, J. van, Sumner, M., Cheng, J., Baston, D., Bevan, A., Bivand, R., Busetto, L., Canty, M., Fasoli, B., et al. (2022). raster: geographic data analysis and modeling. https://cran.r-project.org/web/packages/raster/raster.pdf.

168. Keitt, T., Bivand, R., Pebesma, E., and Rowlingson, B. (2010). Rgdal: bindings for the geospatial data abstraction library. https://cran.r-project.org/web/packages/rgdal/index.html.

169. Pedersen, T.L. (2020). patchwork: the composer of plots. R package version 1.1.1. https://CRAN.R-project.org/package=patchwork.

STAR★METHODS

KEY RESOURCES TABLE

REAGENT or RESOURCE	SOURCE	IDENTIFIER
Biological samples		
mus005	This study	SK5
mus006	This study	SK6
ulu117	This study	M17
CTG025	This study	g25
GOR001	This study	YH36611
GOR002	This study	YH41500
BOG019	This study	373-718
BOG020	This study	605-641
BOG024	This study	107-329
BOG028	This study	020-074
G23	This study	G23
G37	This study	G37
G76a	This study	Π934α, #76; KO 37 / Π920, #62_2
G31	This study	AΠ, #31_6
G62	This study	KO 37 / Π920, #62_1; KO 37 / Π920, #62_3
G65	This study	KO 37 / Π1022 ΛB B4, #65_1
G66	This study	P1008 PB4, #66
geo015	This study	N 39
geo017	This study	N 93
geo005	This study	N1 / Shuagori
geo006	This study	N 8A
geo029	This study	N 14
gur016	This study	N16
gur017	This study	N17
gur019	This study	N19
zrj003	This study	Shamakhi III
sha003	This study	S2, BIII
sha004	This study	S4, BIII
sha006	This study	S9, EII
sha007	This study	S3, EIII
sha008	This study	S5, FII
sha009	This study	S3, FIII
sha010	This study	S4, FIII
sha012	This study	S21, FIII
sha014	This study	S3, GIII
Chemicals, peptides, and recombinant proteins		
RNase Away	Thermo Fisher Scientific	Cat#7000
Sodium hypochloride	Sigma Aldrich	Cat#S7653
HPLC water	Sigma Aldrich	Cat#270733
Ispropanol	Merck	Cat#1009952500
Proteinase K	Thermo Fisher Scientific; New England Biolabs	Cat#E00491; Cat#P8107S
Guanidine hydrochloride	Sigma Aldrich	Cat#50950
Tween-20	BioShop	Cat#TWN508

(*Continued on next page*)

Continued

REAGENT or RESOURCE	SOURCE	IDENTIFIER
Ethanol	Isolab	Cat#920.026.2500
EDTA disodium salt dihydrate	Sigma Aldrich	Cat#E5134
Critical commercial assays		
High Sensitivity DNA Kit (Bioanalyser 2100)	Agilent Technologies	Cat#5067-4626
High Sensitivity D1000 Screen	Tape Agilent Technologies	Cat# 5067-5584
(Tapestation 2200)		
MinElute PCR Purification Kit	QIAGEN	Cat#28004
Qubit dsDNA HS Assay Kit	Thermo Fisher Scientific	Cat# Q32854
Deposited data		
mus005 BAM file	European Nucleotide Archive (ENA)	ERS11167398
mus006 BAM file	European Nucleotide Archive (ENA)	ERS11167399
ulu117 BAM file	European Nucleotide Archive (ENA)	ERS11167409
CTG025 BAM file	European Nucleotide Archive (ENA)	ERS11167387
GOR001 BAM file	European Nucleotide Archive (ENA)	ERS11167396
GOR002 BAM file	European Nucleotide Archive (ENA)	ERS11167397
BOG019 BAM file	European Nucleotide Archive (ENA)	ERS11167383
BOG020 BAM file	European Nucleotide Archive (ENA)	ERS11167384
BOG024 BAM file	European Nucleotide Archive (ENA)	ERS11167385
BOG028 BAM file	European Nucleotide Archive (ENA)	ERS11167386
G23 BAM file	European Nucleotide Archive (ENA)	ERS11167388
G37 BAM file	European Nucleotide Archive (ENA)	ERS11167389
G76a BAM file	European Nucleotide Archive (ENA)	ERS11167390
G31 BAM file	European Nucleotide Archive (ENA)	ERS12566517
G62 BAM file	European Nucleotide Archive (ENA)	ERS12566518
G65 BAM file	European Nucleotide Archive (ENA)	ERS12566519
G66 BAM file	European Nucleotide Archive (ENA)	ERS12566520
geo015 BAM file	European Nucleotide Archive (ENA)	ERS11167393
geo017 BAM file	European Nucleotide Archive (ENA)	ERS11167394
geo005 BAM file	European Nucleotide Archive (ENA)	ERS11167391
geo006 BAM file	European Nucleotide Archive (ENA)	ERS11167392
geo029 BAM file	European Nucleotide Archive (ENA)	ERS11167395
gur016 BAM file	European Nucleotide Archive (ENA)	ERS12566521
gur017 BAM file	European Nucleotide Archive (ENA)	ERS12566522
gur019 BAM file	European Nucleotide Archive (ENA)	ERS12566523
zrj003 BAM file	European Nucleotide Archive (ENA)	ERS11167410
sha003 BAM file	European Nucleotide Archive (ENA)	ERS11167400
sha004 BAM file	European Nucleotide Archive (ENA)	ERS11167401
sha006 BAM file	European Nucleotide Archive (ENA)	ERS11167402
sha007 BAM file	European Nucleotide Archive (ENA)	ERS11167403
sha008 BAM file	European Nucleotide Archive (ENA)	ERS11167404
sha009 BAM file	European Nucleotide Archive (ENA)	ERS11167405
sha010 BAM file	European Nucleotide Archive (ENA)	ERS11167406
sha012 BAM file	European Nucleotide Archive (ENA)	ERS11167407
sha014 BAM file	European Nucleotide Archive (ENA)	ERS11167408
Oligonucleotides		
IS1_adapter.P5: 5’-A*C*A*C*TCTTT CCCTACACGACGCTCTTCCG*A*T* C*T-3’(* indicates a PTO bond)	Meyer and Kircher[57]	Biomers

(*Continued on next page*)

Continued

REAGENT or RESOURCE	SOURCE	IDENTIFIER
IS2_adapter.P7: 5'-G*T*G*A*CTGG AGTTCAGACGTGTGCTCTTCC G*A*T*C*T-3'(* indicates a PTO bond)	Meyer and Kircher[57]	Biomers
IS3_adapter.P5+P7: 5'-A*G*A*T* CGGAA*G*A*G*C-3' (* indicates a PTO bond)	Meyer and Kircher[57]	Biomers
IS4: 5'-AATGATACGGCGACCACCG AGATCTACACTCTTTCCCTACACGA CGCTCTT-3'	Meyer and Kircher[57]	Biomers
IS5: 5'-AATGATACGGCGACCACCGA-3'	Meyer and Kircher[57]	Biomers
IS6: 5'-AAGCAGAAGACGGCATACGA-3'	Meyer and Kircher[57]	Biomers
P5 indexing: 5'-AATGATACGGCGACCA CCGAGATCTACACxxxxxxxACACTCTTT CCCTACACGACGCTCTT-3' (where x is one of 7 different 7 bp indexes)	Meyer and Kircher[57]	Biomers
P7 indexing: 5'-CAAGCAGAAGACGGCAT ACGAGATxxxxxxxGTGACTGGAGTTCAG ACGTGT-3' (where x is one of 22 different 7 bp indexes)	Meyer and Kircher[57]	Biomers
CL72 Sequencing primer: ACACTCTTTCC CTACACGACGCTCTTCC 100/- (IE-HPLC)	Psoni et al.[58]	Biomers
**Software and algorithms**		
AdapterRemoval (version 2.3.1)	Schubert et al.[59]	https://github.com/MikkelSchubert/adapterremovall
BWA aln/samse (version 0.7.15)	Li and Durbin[60]	http://bio-bwa.sourceforge.net/
FilterUniqueSAMCons.py	Kircher[61]	https://bioinf.eva.mpg.de/fastqProcessing/
PMDtools (version 0.60)	Skoglund et al.[45]	https://github.com/pontussk/PMDtools
samtools (version 1.9)	Li et al.[62]	https://github.com/samtools/samtools
ANGSD (version 0.937)	Korneliussen et al.[63]	http://popgen.dk/angsd/index.php/ANGSD
HaploGrep (version 2.4.0)	Weissensteiner et al.[64]	https://haplogrep.uibk.ac.at/
EIGENSOFT (version 7.2.0)	Patterson et al.[65]	https://github.com/DReichLab/EIG
AdmixTools (version 7.0.2)	Patterson et al.[32]	https://github.com/DReichLab/AdmixTools
READ	Monroy Kuhn et al.[66]	https://bitbucket.org/tguenther/read/src
ADMIXTURE (version 1.3.0)	Alexander et al.[67]	https://dalexander.github.io/admixture/download.html
PLINK (version 1.9)	Chang et al.[68]	(https://www.cog-genomics.org/plink/1.9/)
bedtools2 (genomeCoverageBed	Quinlan and Hall[69]	https://bedtools.readthedocs.io/
contamMix (version 1.0-10)	Fu et al.[70]	N/A
bcftools (version 1.9)	Li[71]	https://samtools.github.io/bcftools/bcftools.html
DATES	Chintalapati et al.[36]	https://github.com/priyamoorjani/DATES
PhyloTree (build 17)	van Oven and Kayser[72]	http://www.phylotree.org/
Yhaplo (version 1.1.2)	Poznik[73]	https://isogg.org/
Arlequin (version 3.5)	Excoffier and Lischer[74]	http://cmpg.unibe.ch/software/arlequin35/
bamUtil (version 1.0.14)	Jun et al.[75]	https://genome.sph.umich.edu/wiki/BamUtil
pileupCaller (version 1.2.2)	N/A	https://github.com/stschiff/sequenceTools
hapROH (version 0.3a4)	Ringbauer et al.[46]	https://pypi.org/project/hapROH/0.3a4/
msprime (version 0.7.4)	Kelleher et al.[76]	https://github.com/tskit-dev/msprime
scikit-allel (version 1.3.2)	Miles et al.[77]	https://scikit-allel.readthedocs.io/
**Other**		
Agencourt AMPure XP beads (60 mL)	Beckman Coulter	Cat#A63881
NEB end repair	New England Biolabs	Cat#E6050L

(*Continued on next page*)

Continued

REAGENT or RESOURCE	SOURCE	IDENTIFIER
NEB Quick ligation	New England Biolabs	Cat#E6056L
T4 Polynucleotide Kinase (T4 PNK)	Thermo Fisher Scientific	Cat#EK0032
T4 DNA Ligase	Thermo Fisher Scientific	Cat#EL0011, EL0014
Adenine Triphosphate (ATP)	Thermo Fisher Scientific	Cat#R0441
T4 DNA Polymerase	Thermo Fisher Scientific	Cat#EP0062
dNTP Set	Thermo Fisher Scientific	Cat#R0182, R0181
dNTP Mix	Thermo Fisher Scientific	Cat#R1121, R1122
Bst polymerase, large fragment	New England Biolabs	Cat#M0275S
10X ThermoPol reaction buffer	New England Biolabs	Cat#B9004S
Amplitaq Gold 360 DNA Polymerase (with AmpliTaq Gold Buffer)	Thermo Fisher Scientific	Cat#4398833
KAPA HiFi HotStart Uracil+ Kit	Kapa Biosystems	Cat#KK2801
Herculase II Fusion DNA Polymerase	Agilent Technologies	Cat#600675
10X Tango Buffer	Thermo Fisher Scientific	Cat#BY5
USER enzyme	New England Biolabs	Cat#M5505L
Klenow fragment, including 10× reaction	Thermo Fisher Scientific	Cat# EP0052
UGI	New England Biolabs	Cat #M0281L
FastAP thermosensitive alkaline phosphatase	Thermo Fisher Scientific	Cat#EF0651
DNA Polymerase I, Large (Klenow) Fragment	New England Biolabs	Cat#M0210L
Bst DNA Polymerase, Large Fragment	New England Biolabs	Cat#M0275

RESOURCE AVAILABILITY

Lead contact

Further information and requests for resources and reagents should be directed to and will be fulfilled by the lead contact, Dilek Koptekin (dilek.koptekin@metu.edu.tr).

Materials availability

This study did not generate new unique reagents.

Data and code availability

- The aligned sequence data (BAM format, without filtering for mapping quality) reported in this paper can be accessed and downloaded from the European Nucleotide Archive (ENA) under the following study accession number: PRJEB51705. The BAM files are not trimmed, except for Perachora samples produced in Crete, which include both UDG and non-UDG libraries (STAR Methods). In addition, raw data (FASTQ format) from Perachora samples produced in Crete are also available in SRA (Bioproject ID: PRJNA891271).
- Supplemental information to the present article is available in Methods S1.
- Further tables and figures to support the methodology and the main results are available in Document Z1 in Zenodo. This file contains Tables I-V and Figures I- XXVII.
- Raw data from Figures 4, 5, 6B, and 6C (Tables Z4–Z7) were deposited in the Zenodo database at https://doi.org/10.5281/zenodo.6377228. All figures and data tables located at Zenodo are identified with the prefix Z in the text.
- The genotype data for the 1000 Genomes sub-Saharan African dataset (Dataset 1) can be found in Dataset1.zip file in Zenodo. The code for producing qpAdm input files could be found at https://github.com/dkoptekin/qpAdm-wrapper.
- Any additional information required to reanalyse the data reported in this paper is available from the lead contact upon request.

EXPERIMENTAL MODEL AND SUBJECT DETAILS

Description of archaeological sites and archaeological material

Musular, Turkey

Musular is located in the volcanic Cappadocia region of Central Anatolia, Turkey, on the west bank of the Melendiz river across Aşıklı Höyük, a late 9th and 8th millennium BCE site. Unlike Aşıklı, but similar to two more contemporary and neighboring sites in the close vicinity, Musular is a flat and low site lying directly on the bedrock, a tufa rock formation. Excavations at the site between 1996 and 2004 revealed two occupational phases; an Aceramic Neolithic phase dated to the second half of the 8th-millennium cal BCE and contemporary with the last three building levels of Aşıklı Höyük (2A-C), and a late occupation phase dated to the beginning of the 6th millennium BCE.

The 8th millennium BCE site exposed what appear to be 'unusual' structures.[78–80] These included rock-cut walls and channels, a built channel, a special purpose building that is comparable to the special purpose building of T at Aşıklı,[81,82] both in size, in internal architectural features, and in the lime plastered and red painted floor and walls. Built and rock-cut channels seem to have roles in draining off water and supplying water from the river. The content of the well-preserved intact layered midden consisted of large amounts of animal bones and obsidian tools and wastes, deposited in regular layers. Animal bones, dominated by wild cattle, *Bos primigenius*, were dumped here. Characteristics of the obsidian tools,[83,84] the end-scrapers, pressure retouched projectiles, cutting tools, etc., signified hunting and post-hunting activities. Use wear analysis[85] suggests on-site butchering and hide processing. Cutting operations covered meat and fresh hide whereas scrapers also suggested skin processing and scraping activities.

The evidence thus indicates that Musular was a special activity site based on wild cattle hunting.[86] Cattle were first slaughtered at the hunting spot; initial chopping was performed at the location of the kill and then the prey was brought onto the site to be butchered. The hide and the meat were cut and the hides were processed, likely accompanied by rituals. The special building could have hosted ceremonies as part of the hunting activity. Layers of bones suggest communal consumption that accompanied the rituals. Musular most plausibly was founded by Aşıklı inhabitants around the mid of the 8th millennium BCE when a radical change in the habitation sequence is observed.

Burials SK 5 (mus005) and SK 6 (mus006) date to the Aceramic Neolithic occupation at Musular.

SK 5 (mus005). This is an adult female aged around 25-35 years. Her lying position is unclear due to post-deposition disturbances. No burial goods were found associated with the burial.

SK 6 (mus006). This is a male in his early 20's. The burial was exposed under the red-painted floor building at Musular. No burial goods were found associated with the burial.

Ulucak, Turkey

Ulucak Höyük is located 25 km east of İzmir in west-central Turkey. The mound is located in the western part of the Kemalpaşa plain. The plain is surrounded by the Nif and Spil mountains at its southern and northern ends respectively and is fed by the Nif river, a tributary of the Gediz River. Ulucak is a small mound covering an area of ca. 1 ha with 11 m of stratigraphic sequence. Continuous occupation at the site occurred from Phase VI through Phase IV (6850-5700), with habitation in Phase III (5600–5460 cal BCE) following a brief cultural break. Later phases belong to the Early Bronze Age (Phase II) and Middle Bronze Age (Phase I), with evidence of Late Roman / Early Byzantine remains on the surface.

During the 2018 field season at the site, a small sondage, 2 x 1.5 m, was dug in the western end of the mound (Trench M7), to understand the extension of the prehistoric occupation. No evidence for Neolithic occupation has been found in this sondage, while an isolated child skull (Ulucak'18 M7a - ulu117) together with some fragments of human and animal bones (including bones of a likely adult female: Ulucak'18 M7b) were found in the fill between the Early Bronze Age stone platform and the virgin soil. Accordingly, the skull and other bone fragments can be dated to the post-Neolithic period, after 5,600/5,500 BCE and before 3,000 BCE.

ulu117 (Ulucak'18 M7a). The test trench at Ulucak Höyük revealed the disarticulated cranium and mandible of a likely 5-6.5 years-old child. On the vertex of the skull, 10 mm behind the bregma region, an oval-shaped depressed trauma (13-33 mm sized) was detected. The trauma has a concentric fracture line and four radiating fracture lines related to blunt-force trauma. It seems that the individual died due to blunt-force trauma. The skeletal material could not be radiocarbon-dated due to a lack of collagen preservation.

Çine-Tepecik, Turkey

Çine-Tepecik is a mound located on the plain traversed by the Çine Stream, one of the southern tributaries of the Büyük Menderes River. Excavations led by Prof. Dr. Sevinç Günel since 2004 have revealed evidence that sheds light on the scarcely known prehistory of the Aydın region.[87,88] The earliest cultural remains in Çine-Tepecik are dated to the Chalcolithic (Late Neolithic in the Aegean chronology), and the history of settlement can be traced until the Carian-Geometric period. Meanwhile, in the Hellenistic and Roman periods, the mound was used as a cemetery. Starting from its earliest layers, Çine-Tepecik exhibits a settlement plan growing in size through the chronological sequence. The Chalcolithic period, reflecting earlier evidence of settled life, maintained a lifestyle based on agriculture and animal husbandry and included a technological toolkit that made extensive use of raw materials. Mortuary traditions in the Chalcolithic and the Early Bronze Age were characterized by infants buried in jars with grave goods, while females were interred in pithoi.

The way of life and technological advances of the early communities in Çine-Tepecik played an important role in shaping the urbanization of the 2nd mil. BCE.[89] These developments culminated in a strong, fortified settlement in the Late Bronze Age layers of Çine-Tepecik. Square towers were attached to the fortification walls at regular intervals, highlighting the defensive requirements

of the urbanism that emerged. A structured socio-economic configuration is visible in the storage and workshop areas within the walled settlement. Facilities that served storage purposes, where products were stored in pithoi, also contained archaeological and philological indicators of interregional trade. Pithoi used to store cereal products yielded seal impressions that date to the Hittite Empire period, suggesting a formally administered economic apparatus. At the same time, Mycenaean-painted wares recovered from the settlement testify to the connections with the Aegean.[90] From the Chalcolithic to the end of the Late Bronze Age, Çine-Tepecik appears as a center that engaged in cultural and commercial activities with the Aegean in the west and with Central Anatolia in the east. Within the historical geography of western Anatolia, Çine-Tepecik is positioned to the south of the land of Arzawa.[91]

Çine-Tepecik G25 (CTG025). The grave was discovered in 2012 in grid-squares I-II/d-e in trench M/12. The burial is a simple earthen grave, and the individual was placed on the right side in the hocker form. The degree of preservation of the bones of this individual is moderate. As a result of the bioarchaeological examination, it was determined that the individual was an adult female (probably 30-35 years old). No specific infection was found on the skeleton, but osteological analysis suggested that the individual had maxillar sinusitis, osteoarthritis in her joints and vertebrae, and also a healed fracture on the left radius.[92,93]

Gordion, Turkey

Gordion is a mound located in Central-West Anatolia, 65 km southwest of Ankara. Gordion thrived particularly in the 9th and 8th centuries BCE, as the capital of the Phrygia, an Iron Age kingdom in Anatolia, but its Citadel Mound includes settlements that span from the third millennium BCE to the 2nd millennium CE, with hiatuses in between.[94] Excavations of about 40 tumuli (elite burial mounds) and three lower status cemeteries at Gordion have provided a large and richly varied assemblage of human skeletal material and associated contextual information, dating from the 17th century BCE to the 5th century AD.[95–99] The most celebrated discovery is a Phrygian royal burial found inside the monumental Tumulus MM, dated ca. 740 BCE, with the skeletal remains of a man aged around 60 years. To date, very few studies have been conducted on the several hundred human skeletal remains excavated at Gordion, and there has also been rather limited analysis of the varied mortuary practices represented. For this reason, commencing in 2015, forensic archaeological and osteological studies were initiated on the skeletal materials stored in the Gordion excavation house depot, by experts from different disciplines, in particular forensic archaeology, anatomy, trauma, and ancient DNA. Among these, Tuğba Gençer is conducting an archaeological and osteological investigation of the skeletal remains from Gordion's Lower Town ("Area A" and "Area B" excavation trenches), from burials dated to the Hellenistic and Roman periods (late 4th century BCE – 2nd century CE). Current work is focusing on the pre-Roman Celtic Galatian phases (3rd–1st centuries BCE) from the Lower Town, using osteological and DNA analyses, as well as a study of burial practices, to ascertain to what extent the remains represent the Galatian communities who migrated from southeastern Europe into Anatolia in the 3rd century BCE, or the local families already living in the area when the Galatians arrived.

In parallel with the osteological studies on individuals from Lower Town Area A, bone samples were taken from five of these skeletons and sent to METU for DNA analysis, including individuals YH36611 (GOR001) and YH41500 (GOR002). Morphological studies have revealed that YH36611 was male (age: 30-35 yrs, height: ca. 1.75 cm) and YH41500 was female (age: 50+ yrs, height: ca. 1.55 cm). Both can be assigned to a time period between 333-0 BCE based on their archaeological context.

Boğazköy-Ḫattuša, Turkey

The archaeological site of Boğazköy, (located in the Çorum province, Northcentral Anatolia, Turkey) is most famous for serving as the capital city of the Hittite Empire between c. 1,650 and 1,180 BCE. The Bronze Age city is located in a rugged landscape at the transition between the steppes dominating central and southern parts of inland Anatolia and the southern extensions of the Pontos Mountains. The Hittite city (enlisted as a UNESCO world heritage site) covers roughly 186 ha and is dominated by numerous monumental official and public buildings, serving mainly as a center of cult and political power.[100] Research by the German Archaeological Institute, continuing since 1931, has uncovered not only the largest Bronze Age city of Asia Minor but also produced numerous findings to reconstruct the settlement history of the region starting from the Chalcolithic period to the early modern era.

Unfortunately, burials of the Bronze Age and especially of the Hittite Period are virtually unrecorded in modern excavations; probably because the Hittites preferred extra mural interments. During the Iron Ages, people preferred cremation, burying the ashes in small urns. However, an extensive necropolis of the Hellenistic-Galatian and especially the Roman Imperial period provides insights into the rural population of a remote region of central Anatolia. This necropolis is located in the lower town, north, west and south of the Great Temple of the Hittite period. It is characterized by a large variety of burial types possibly reflecting differences in social habits.[101] Radiocarbon dates as well as coins and ceramic finds demonstrate that the necropolis was used from the 3rd century BCE until the second half of the 4th century CE.[101,102] Three of the individuals included in this study are part of this large burial site. A fourth burial shows that the site was used in later periods at least occasionally.

BOG19 (Boğazköy 2009, 373-718, 291/374). The burial was found in the southern extension of the Hellenistic/Roman necropolis during the 2009 excavation season. It was a simple inhumation. The skeletal remains belong to a middle-aged male. Based on coins found nearby, the burial was dated to the 4th century CE.[103]

BOG020 (Boğazköy 2015, 605-641, 295-407). The burial was found in the northern extension of the Hellenistic/Roman necropolis in 2015. It was an inhumation covered by roof tiles with no grave finds. The skeletal remains of a middle-aged man found here were not dated. However, the C14 dates of the five burials found nearby indicate that this burial must have belonged to the same period, that is, 2nd to 4th centuries CE.

BOG024 (Boğazköy 2017, 107-329, 293-406). This burial, found in 2017, also belongs to the northern extension of the necropolis. The skeletal remains of an old man were found on the side of a wall in a supine position in NE-SW direction. There were no grave finds associated with the skeleton. Although no C14 dating is available, it is likely that this burial belongs also to the 2nd and 4th centuries CE, based on C14 results of other nearby burials.

BOG028 (Boğazköy 2017, 020-074, 293-407). This burial, which was found in the northern extension of the necropolis in 2017, contained the remains of a 6-year-old girl. The body, which was placed in the NW-SE direction, facing NE, was surrounded by stones. There was no grave finds in association with the skeleton except a bronze fragment. A group of child burials was found in this area, similar to this burial. Although there is no C14 date, it is estimated that these children's graves belong to the Modern Age due to the well-preserved bones and some finds found around them.

Theopetra cave, Greece

The Theopetra cave is located by a river setting on the northeast side of a limestone rock formation, 3 km south of Kalambaka in Thessaly, in central mainland Greece. It is one of the most significant prehistoric sites in Greece, providing a long stratigraphic sequence documented by material culture and bioarchaeological data. In particular, the site gives evidence for the transition from the Pleistocene to the Holocene, as well as all archaeological periods from the Middle and Upper Paleolithic to the Neolithic period and the Bronze Age. Aiming at a reliable interpretative scheme, complementary analytical methods were applied to findings from the cave including C14 dating of a large number of organic materials including bones, thermoluminescence dating, micromorphological study of sediments, anthropological analysis of skeletal remains, histopathological, stable isotope and DNA analysis of selected human specimens, petrographic and chemical analysis of pottery, microware analysis of lithics, and microscopic analysis of botanical remains.

In the deeper layers of the Middle Paleolithic in the cave, evidenced by the lithic operational sequence and the taxonomy of the archaeozoological material, within a distinct anthropogenic layer a unit of human footprints of two children with traces of a cover was uncovered.[104] These probably belonged to Neanderthals based on the typological profile of the associated stone tools of Mousterian technology, dated around 130,000 BCE.

Similarly, in addition to lithic findings and archaeozoological remains documenting the Upper Paleolithic horizon there were also two human burials, which correspond to the post-glacial Upper Palaeolithic period. Bone from one of these burials was radiocarbon dated to 14,990-14,060 BC,[105,106] and there were also successful results of the first attempt of aDNA analysis of the skeleton.[107] A human-made stone wall at the entrance of the cave, dated around 21,000 BC, was probably constructed to offer protection from natural phenomena and is a rare finding worldwide.

The presence of Mesolithic remains in the stratigraphy of the Theopetra cave enhances the debate on this transitional period to a new environmental, economic and biosocial basis. Data from this site show continuity from the Upper Palaeolithic to the Mesolithic period in mainland Greece. At the Theopetra cave the Mesolithic period is documented by the microlithic technology, the horizons of shell midden, and mainly by the human burials in situ,[108] dated between 7000 and 7500 BC. Both individuals were found to belong to mtDNA Haplogroup K1c.[40] The archaeobotanical material and the presence of unbaked clay as well as some sherds in the Mesolithic layers of the cave are of special importance with respect to the transition to the Neolithic economy.

The Neolithic horizon, although disturbed due to geological episodes,[109] is represented by living floors, hearths and working areas, a variety of ceramic types for household activities and a broad range of lithic finds mainly from obsidian and polished stone, indicating both local work and trade exchange networks. The minimum number of individuals from this period was calculated as 43. These include mainly young adults and subadults in good health. Some anatomical elements indicate possible inhumation.[110,111] Wheat, barley, olives, lentils, wild pear and pulses, the presence of which is also confirmed by archaeobotanical remains, were probably a main nutritional source, however animal fats and vegetable oils were also identified by organic residue analysis. There is strong evidence that their diet included meat mainly from domesticated animals, a few of which were kept for their by-products (wool, milk). It is worth mentioning that cut marks and knife imprints were traced on a bone of a bear indicating in situ activities. In an undisturbed section of the Neolithic horizon a number of jewels, drilling holes into deer-like teeth and shells from the nearby river, were also uncovered.[112,113]

Human use of the Theopetra cave during the Early Bronze Age period is documented by scarce ceramic finds dispersed within Neolithic material in the central and back spaces of the cave chamber within an area of stratigraphic disturbance. Profiles of the pottery, handmade polished and fine-grained texture bowls and jars suggest the presence of a range of domestic vessels possibly for cooking. Few scattered human and animal bones were traced across this stratigraphic sequence without any indication of burial practice. During this period, evidence for human activity dates from the earlier phase of the Early Bronze Age, although most of the material seems to be from its later phase.

The Theopetra cave is a key site for the prehistory of Greece, southeastern Europe and adjacent regions, proposing new schemes toward a paradigm shift in the archaeological theory via interdisciplinary research with science.

Sample G23 is from a tooth extracted from a mandible (lower jaw).

Sarakenos cave, Boeotia, Greece

The Sarakenos cave is formed in a limestone block within the area of Kopais lake, a natural basin in the northeastern part of Boeotia in mainland Greece. The Sarakenos cave excavation was part of a research project aiming to survey the anthropogenic horizons of the karstic formations around the rocky boundaries of the Kopais lake basin. The archaeological research has mapped 23 caves of low elevation at the level of the past lakeshore border. Out of these sites, the Sarakenos cave is the most important archaeological cave site.

The main excavation trenches revealed Middle and Early Helladic levels and also reached a Neolithic layer. The last phase of the late Neolithic in the cave (3706-3549 BC) is significantly extended, while the early and middle Neolithic are also present in the sequence. These upper layers have yielded pottery and rich organic remains including animal and fish bones, and freshwater shells. The layers following probably represent a hiatus in the occupation of the cave. A deep test trench reached bedrock and showed the sequence of the deposits at least in one part of the cave.

The lowest stratum, a thin layer resting on the bedrock, is dated to the beginning of the Upper Palaeolithic or the end of the Middle Palaeolithic period, as is testified by the stone industry that comprises blade-type implements of the Aurignacian and the Mousterian period.[114] Small charcoal particles from the upper part of the Palaeolithic deposit were dated to 13,100-12,150 BCE.

Layers of burnt material from hearths provide evidence for the use of the cave towards the end of the Palaeolithic or the beginning of the Mesolithic, which is also supported by absolute dating of charcoal samples from this stratum (8,530-8,340 BCE; 8,450-8,290 BCE; 8,530-9,340 BCE). By applying optical thermoluminescence an analogous age of 10110+/- 750 BP was attested.

Soil, charcoal samples and charred seeds from the cave offered solid data about the palaeoenvironment in the Kopais basin from the Palaeolithic to the Middle Bronze Age. The pollen diagrams of the palynological assemblages from the Sarakenos cave deposits and the presence of specific plant species show the impact of humans on the environment of the Kopais basin from the second half of the 5th millennium to the 2nd millennium BC,[115] when the cave was probably abandoned for unknown reasons.[116] Possibly this was due to the drainage of the lake, which may have been started at the end of the Middle Bronze Age (Middle Helladic) period.

The Sarakenos cave presents overall similarities and analogues with other caves and open sites in Greece and the Aegean, and exhibits finds of special significance. Beyond household activities such as living floors (some with holes of piles), housing livestock, storage, long-term habitation, shelter, processing of raw materials, identified from the remains of numerous ceramic vessels of high quality, chipped stone tools, weaving accessories, animal bones and food remains, a large assemblage of figurines depicting humans have also been uncovered. Although the presence of figurines within the Neolithic contexts of the cave is not an unusual finding, the Sarakenos cave is a rare site in that the figurines made out of marble and clay count hundreds, span many periods of occupation and exhibit a variety of features depicting the face and clothes.[116–118]

Unlike the Palaeolithic period, during the Neolithic and the Bronze Age periods human occupation in the Kopais basin appears to be present both in open air and in cave sites. During these periods there is ample archaeological evidence from the Sarakenos Cave for the regular exploitation of aquatic resources.[117,119]

The Sarakenos cave offers a parallel to the Theopetra cave in mainland Greece. They are both pilot study sites developing a long rigid biostratigraphic and cultural sequence from the Palaeolithic to the Bronze Age using new analytical reliable methods.

G37 is a tooth sample extracted from a mandibular fragment recovered from the upper layers of the Sarakenos cave in Boeotia.[114,119]

Perachora cave, Greece

The cave of Lake Vouliagmeni at Perachora is formed on the southern slope of a limestone hill to the west of the lake, at an altitude of 50 m and 500m, in Corinthia in Greece. In the surrounding area, at 300m from the cave, an open settlement site dating to the Early Bronze Age period,[120] and, at a short distance, a burial ossuary pit was also discovered.

Rescue excavations in 1992 by the Ephorate of Paleoanthropology-Speleology of the Greek Ministry of Culture revealed a large number of intact skulls placed along the walls of the cave, as well as cranial and postcranial remains as scattered bones, few pottery sherds and two lithic tools of flint and obsidian in the sandy sediment, the main anthropogenic horizon in the cave consisting of three sublayers. Based on the absence of articulated skeletons and the pattern of spatial distribution of postcranial bones inside the Perachora cave, the site was identified as a cave ossuary, as those known from other Bronze Age sites in Greece, mainly in the Cyclades and in Crete. In terms of the placement of skulls in ossuaries, especially in caves, as attested in the Early Bronze Age Perachora cave, there are parallels from a few sites in Attica and Crete in Greece, where research at the very important Agios Charalambos cave in the eastern part of the island has revealed evidence for similar skull treatment.[121]

The pottery sherds from the cave are mostly classified as coarse pottery for household activities, mainly saucers, plates and sauce boats. However, a remarkable proportion of decorated sherds with the whole surface painted was also recorded. Although it is not possible to relate the sherds with the human bones, it is very likely that they were also used during the burial process. The relative chronology based on the typological categories of the ceramic findings, and especially comparison with the decorated pottery from other sites in central Greece, in Corinth and in the Argolid in the Peloponnese, suggest the cave was used within the period 2,750 BCE to 2,450/2,200 BCE.[120]

Based on the bioarchaeological analysis of the skeletal remains, the minimum number of individuals was calculated as 92. These include infants, subadults and young adults of a relative male: female ratio of 60:40. Acknowledging the inherent biases and limitations posed by the statistical sampling and the absence of other tissues in the study of ancient bone, integrated macroscopic, radiological, scanning electron microscopy (SEM), endoscopic, spectroscopic, and histopathological analyses of skeletal material from the Perachora cave provide evidence of mild anemic episodes for few individuals and *intra vitam* vestiges of inflammatory reaction in most of the specimens. Interestingly, in almost all cases remodeling of the diseased areas, as a healing state, is evidenced.[111] Regarding anemia, it would be convenient to support a genetic anemia incident linked to malaria/anemia balanced polymorphism mechanism, manifested in marshy areas such as the lake of Perachora. Such a diagnosis can potentially be supported by molecular analysis,[122] while other types of anemia cannot be diagnosed. In contrast to the results of laboratory analysis of bone samples from

other cave sites in Greece, where severe diagenetic events due to geochemical processes have been traced, the Perachora skeletal material is of satisfactory taphonomic preservation. This is crucial for the successful application of reliable analytical techniques to generate physical evidence for biocultural interpretation.

The bioarchaeological profile of the Perachora cave makes the site a pilot case study considering the deliberate burial behavior with a selective process in the management of anatomical elements and the deposition of the skulls, which is not often found in a cave ossuary. The site is also important for the significant number of specimens recovered and, in terms of demographic variables, for the relatively broad range of age-categories represented. The preliminary data enrich the database on histopathological lesions building up a possible health scenario by applying reliable analytical methods.

Based on the results of the Perachora cave study, beyond the archaeological, anthropological and bioarchaeological parameters, there are many aspects of interest. These include the chronological spectrum during which the cave was used, its geographical proximity and the spatial relation with the nearby settlement and tomb,[123] and its instrumental use by different or related groups/communities -a field of hot debate in research. The burial pattern at the Perachora cave is an essential human institution since it seems to make a shift towards the definition of an ossuary and secondary burial sites, where the skulls are treated in a cautious and time-investing manner like primary burials. The Perachora cave assemblage offers unique insights into the cultural contexts in which it was generated and the associated rituals/activities.

G31 was obtained from a left side petrous bone (Crete aDNA Lab ID: ADNA_100031_6, Cave ossuary main chamber: AΠ, #31_6) from the Perachora cave ossuary.

G62 was obtained from a right side petrous bone (Crete aDNA Lab ID: ADNA_100062_1, Cave ossuary main chamber: KO 37 / Π920, #62_1) and a left side petrous bone (Crete aDNA Lab ID: ADNA_100062_3, Cave ossuary main chamber: KO 37 / Π920, #62_3). The libraries obtained from 62_1 and 62_3 (see Table S1) were identified as "Identical/Twin" by the READ algorithm; thus we merged them as G62 (see Figure I in Document Z1 and Table Z9 in Zenodo).

G76a was obtained from a right side petrous bone (Crete aDNA Lab ID: ADNA_100062_2: Cave ossuary main chamber: KO 37 / Π920, #62_2) and a tooth sample extracted from an incomplete mandible (Stockholm aDNA Lab ID: G76a, Cave ossuary main chamber: Π934α, #76) from the Perachora cave ossuary. The libraries obtained from 62_2 and G76a (see Table S1) were identified as "Identical/Twin" by the READ algorithm; thus we merged them as G76a (see Figure I in Document Z1 and Table Z9 in Zenodo).

G65 was obtained from a left side petrous bone sample (Crete aDNA Lab ID: ADNA_100065_1, Cave ossuary main chamber: Π1022 ΛB B4, #65_1) from the Perachora cave ossuary.

G66 was obtained from a left side petrous bone sample (Crete aDNA Lab ID: ADNA_100066, Cave ossuary main chamber: P1008 PB4, #66) from the Perachora cave ossuary.

Inside the cave, the bones were recovered together with pottery, whose typology suggests a long period of use during the Early Bronze Age (Early Helladic period). The radiocarbon date measured from G76a falls in the middle part of this period.

Didnauri, Georgia

The Didnauri town / cemetery is located near the Iori River gorge in southeastern Georgia. Archaeological excavations began in 2016 by the archaeologist Prof. Konstantine Pitskhelauri. Based on radiocarbon dates as well as clay and bronze artefacts, Prof. Pitskhelauri assigns the Didnauri settlement and burial ground to the late 14th century and the 13th century BCE. It is estimated that 5-6 more such ancient cities existed in the same region in this period.

The tombs are presented in the form of kurgans. Until now one cemetery containing 32 tombs has been excavated, while excavation has also started on another five tombs. The skeletal samples used in this study were excavated in this first cemetery (skeletal numbers: MMKPH 1-32). The osteological materials are preserved in the Museum for the History of Georgian Medicine.

The three skeletal samples from Didnauri analyzed here were geo005 (N1 / Shuagori), geo006 (N 8A) and geo029 (N14). These were dated to ca. 1,250-850 BCE. There was insufficient information to determine their age or sex.

Doghlauri, Georgia

The Doghlauri cemetery was excavated as part of a salvage excavation under the directorship of E. Rova (Venice Ca'Foscari University) and I. Gagoshidze (Georgian National Museum). The site is located in the Georgian Shida Kartli region and is believed to have been used as the cemetery of the neighboring Aradetis Orgora settlement. Excavations revealed structures across a wide area, attributed to the Kura-Araxes period (3,500-2,500 BCE) and the Late Bronze Age/Iron Age period (c.1,500-700 BCE). Graves were found in structures belonging to both periods. Doghlauri represents one of the largest ever excavated cemeteries of the Georgian Bronze Age, with c.450 burials excavated. The Kura-Araxes graves frequently contained multiple burials, and pottery and occasionally weapons and metal objects were found as burial goods. The LBA graves were mostly single burials and were relatively richer in goods. Both sexes and a wide range of ages were represented in burials of either period.[43]

Of the two skeletal samples from Doghlauri analyzed in this study, the geo015 (N 39) individual belonged to an adult male (based on osteological analysis) from the Kura-Araxes period, while the geo017 (N 93) individual belonged to the Late Bronze Age period.

Nazarlebi, Georgia

The archaeological site of Nazarlebi, located on the Shiraki plain in Kakheti, Eastern Georgia (N41.339492, E46.238252), is classified as a Late Bronze Age settlement, and sanctuary (ca 15th to 9th centuries BC). The present-day landscape type of the Shiraki Plain, which lies between the Iori and Alazani rivers, is a steppe with gently rolling hills, mostly used for grain crops and pastures. Nazarlebi was originally a natural hill, which was transformed into a fortress-like complex with the help of ring-shaped terraces and ramparts.

Findings at Nazarlebi include burials, circular shrines, skeletal remains of different animals (incl. humans), and a multitude of Bronze Age artefacts. This site is notable for its large deposits of weapons made of bronze. Nazarlebi is part of the Bronze Age culture that occurred in the eastern South Caucasus.[124,125]

gur016 (Nazarlebi I, Korgan 1, Grave 7, Child Skull and other skeletal remains, Excavated on 25.11.2007).

gur017 (Nazarlebi I, Korgan 1, Grave 4, Human baby skull and other skeletal remains, Excavated on 25.11.2007).

gur019 (Nazarlebi I, Korgan 1, Grave 13, Human skull and other skeletal remains, Excavated on 25.11.2007).

Shamakhi, Azerbaijan

The site Shamakhi in Azerbaijan was originally excavated in 1960. The individual zrj003 (Shamakhi III) is thought to represent the burial of a commoner during the Antiquity period in this region. The individual featured a mesocephalic skull and was inhumed within a pit grave in an extended position. Undecorated ceramics accompanied the burial.[126]

Shah Tepe, Iran

The site at Shah Tepé was investigated in 1933 by T.J. Arne[127] in collaboration with Iranian authorities. Some of the finds and skeletal remains of humans were sent to Sweden after the examinations. The human remains were first sent to Lund to C.M. Fürst who performed an osteological analysis of the material.[128] Today, the skeleton material is incorporated into the collections of the Museum of Mediterranean and Near Eastern Antiquities in Stockholm. Through the publications by Arne and Fürst, the find circumstances of the burials may be reconstructed. The excavations were carried out in trenches and layers. Arne identified three main chronological phases (I-III), where Phase III is the oldest one[127] and dated by Arne to the time around 3,200-2,900 BCE while period II fell between 2,900-1,800 BCE which was further divided into three phases, phase IIb to 2,900-2,300, phase IIa2 to 2,300-2,000 and phase IIa1 to 2,000-1,800 BCE. The last period was dated to c.700-800 CE. The division and dates are, however, not reliable when considering more recent research.[41,42] Four radiocarbon dates provided here on human remains, all from the deepest layer (III) of the site, indicate that the burials fall in the Late Chalcolithic and the Bronze Age, approximately between 3,350-3,050 cal BCE. One dating of an individual that did not exhibit preserved DNA is slightly younger. Some of the burials at Shah Tepe had been dug through house foundations. The settlement was a village-like center.

The field documentation provides a view of the stratigraphic conditions at the site. There are a total of 95 individuals in the museum's collections, of which 39 belong to Phase I, 28 to phase II, and 19 to phase 3. The remaining individuals could not be linked to the exact found location. Through drawings and published documentation, the site can be found for most burials identified. In addition, there is osteological data and data on body position and grave goods, often ceramics that exhibit specific features.

Descriptive data on the samples from Shah Tepe. Trench, Layer, and skeleton number according to Arne.[127]

Name of Sample	Trench, Layer	Skeleton number	Material	Dating, BP	$\delta^{13}C$/ $\delta^{15}N$/(C:N)	ID
Sha001, No data	AIII	S2	Tooth	3998±33	-20.6/8.7 (3.2)	Ua-70797
Sha002, No data	BII	S8	Petrous bone	-	-	-
Sha003	BIII	S2	Tooth	-	-	-
Sha004	BIII	S4	Tooth	4560±33	-19.6/10.4 (3.2)	Ua-70798
Sha005, No data	CII	S8	Petrous bone	-	-	-
Sha006	EII	S9	Tooth	-	-	-
Sha007	EIII	S3	Tooth	4544±33	-19.4/10.4 (3.2)	Ua-70799
Sha008	FII	S5	Petrous bone	-	-	-
Sha009	FIII	S3	Petrous bone	4484±33	-20.5/9.0 (3.5)	Ua-70800
Sha010	FIII	S4	Petrous bone	-	-	-
Sha011, No data	FIII	S16	Occipital bone	-	-	-
Sha012	FIII	S21	Tooth	-	-	-
Sha013, No data	GII	S7	Petrous bone	-	-	-
Sha014	GIII	S3	Tooth	-	-	-

Abbreviations

BP = Before Present, BCE = Before Common, CE = Common Era, E = Early, M = Middle, L = Late, HG = Hunter-gatherers, N = Neolithic, CA = Chalcolithic, BA = Bronze Age, IA = Iron Age, MP = Medieval Period, CHG = Caucasus Hunter Gatherers, WHG = Western Hunter Gatherers, EHG = Eastern Hunter-Gatherer, WSHG = West Siberian Hunter Gatherers, ROH = Runs of Homozygosity, MAF = minor allele frequency

METHOD DETAILS

Sample preparation

Samples were prepared at the aDNA laboratories of METU and Hacettepe (Ankara, Turkey), the Centre for Palaeogenetics (CPG) (Stockholm, Sweden) and the Ancient DNA Lab at FORTH (Heraklion, Greece) (Table S1).

Ankara

Samples were processed at the aDNA laboratories of METU and Hacettepe Universities (Ankara, Turkey). Both laboratories followed the same procedures to extract DNA and construct libraries. Prior to DNA extraction, the surface of bones and/or tooth samples was decontaminated with a 0.5% sodium hypochlorite solution and UV irradiated in a crosslinker (6 J/cm2 at 254 nm). After decontaminating the bones, approximately 120mg of bone was cut out and ground to fine powder in the SPEX 6875 freezer mill. DNA was extracted and purified following the steps in Dabney et al.[129] Double-stranded, blunt-end, Illumina compatible sequencing libraries were prepared using 20ul of the DNA extracts as described in Meyer and Kircher.[57] Negative controls at every step of DNA extraction and library preparation were also included to assess contamination. The number of PCR cycles for the enrichment of each library was determined using real-time PCR (qPCR). Next, the enriched libraries were purified using AMPure beads and then screened for DNA content using low-coverage shotgun sequencing on the Illumina HiSeq or Novaseq 6000 platforms (at the Science for Life Laboratory in Stockholm). Finally, samples that yielded roughly $\geq$1% authentic human DNA showing aDNA-related post-mortem damage ($\geq$15% C$\rightarrow$T transitions at the first position of 5' end)[45,130] were re-sequenced further for deeper coverage (see Table S1).

Stockholm

Samples were prepared at the aDNA laboratory of the Centre for Palaeogenetics (CPG) (Stockholm, Sweden). The surface of bone and/or tooth samples was decontaminated with a 0.5% sodium hypochlorite solution and UV irradiated (6 J/cm2 at 254 nm). Bone was drilled to powder, and the tip root of the teeth was cut with a multitool drill (Dremel) to obtain approximately 80 to 150 mg of bone powder/root tip. DNA was isolated and purified following Dabney et al.[129] or Krzewinska et al.[131] protocols. Illumina sequencing libraries were prepared using 20ul of the DNA extracts as described in Meyer and Kircher.[57] All standard measures were taken to prevent exogenous DNA contamination, including the use of library negative controls (extraction blanks) and PCR blanks in every step of library preparation and amplification. Real-time PCR (qPCR) was used to determine the number of PCR cycles for each library during the amplification step. Double-stranded, blunt-end libraries were first screened using low-coverage shotgun sequencing on the Illumina HiSeq X or Novaseq 6000 platforms (at the Science for Life Laboratory in Stockholm). Next, we chose the samples that yielded roughly $\geq$1% authentic human DNA showing aDNA-related post-mortem damage ($\geq$15% C$\rightarrow$T transitions at the first position of 5' end)[45,130] and re-sequenced these for obtaining deeper coverage (see Table S1).

Crete

Six petrous bone samples from Perachora were prepared in the cleanroom facilities of the Ancient DNA Lab, Institute of Molecular Biology and Biotechnology (IMBB), Foundation for Research and Technology - Hellas (FORTH) (Heraklion, Greece), following strict ancient DNA guidelines (e.g., Fulton et al.[132]). Negative extraction and library controls (water blanks) were included in all cases to monitor for contamination. All post-library preparation steps (i.e., qPCR, library amplification and indexing, indexed library purification and quantification) were performed (with the negative controls wherever applicable) in a standard molecular biology lab located in a different building. Sequencing was performed in the Genomics Facility of IMBB-FORTH.

Sample processing and DNA extraction. For all petrous bones (490-1116 mg of powder) processing and DNA extraction were performed following established procedures[133] with a few modifications.[58,134]

Double-stranded library preparation and indexing for initial screening. For all samples, DNA extract was built into a blunt-end library according to procedures (library preparation, quantification, indexing) previously described[133] with a few modifications.[134,135] Shallow shotgun sequencing (screening) was performed on an Illumina NextSeq 500 platform using 75+6 bp paired-end read chemistry (2 × 75 bp plus 6 bp index). These libraries in Table S1 follow a coding format of ##_#_DS_1.

Double-stranded library preparation for deeper sequencing. After initial examination of the screening results and confirmation that all samples a) are characterized by an ancient DNA-like post-mortem damage profile and b) have high human endogenous DNA content, new libraries were prepared, this time from DNA pre-treated with the USER enzyme [uracil-DNA-glycosylase (UDG) and Endonuclease VIII (EndoVIII)] (New England Biolabs, Ipswich, MA, USA) as described in Psonis et al.[136] Library preparation, quantification and indexing were performed as described above. Deep sequencing was performed on an Illumina NextSeq 500 platform using 75+6 bp single-end read chemistry (75 bp plus 6 bp index). See Table S1 for sequencing statistics of these libraries that follow a coding format of ##_#_DS_UDG_L2.

Additional sequence data for sample ADNA_100031_6. This sample has been previously used in a recent study to test different modifications on the library preparation techniques,[136] albeit without being incorporated in a population genomics analysis. Hence, sequence data from 25 additional libraries were used for this sample. Details on library preparation, quantification, indexing, and sequencing procedures can be found in the corresponding study. Moreover, additional libraries have been prepared for this sample in our lab to test different library protocols, enzymes, and initial DNA quantities used. The six double-stranded libraries including the term "BEST" in their code name have been prepared using the BEST protocol of Carøe et al.[137] with or without the use of UDG or EndoVIII (as in Psonis et al.[136]), whereas the four single-stranded libraries including the term "2.55" have been prepared using the protocol of Gansauge et al.[138] with modifications as in Psonis et al.[136] For all ten of them, quantification, indexing and sequencing procedures were also the same as in Psonis et al.[136]

Radiocarbon dating

Fifteen individuals were C14 radiocarbon dated by the TÜBİTAK- MAM (mus005, mus006, CTG025), Beta Analytic Radiocarbon Dating Laboratory (G23, G37, G76a) and by the Tandem Laboratory at Uppsala University (geo005, geo006, geo015, geo017, geo029, zrj003, sha004, sha007, sha009) (Tables 1 and S1). All dates were calibrated using IntCal20.[139]

QUANTIFICATION AND STATISTICAL ANALYSIS

Sequence data processing

For each library, we removed the residual adapter sequences in raw FASTQ files by using the software "*Adapter Removal*" (version 2.3.1) using "*–qualitybase 33 –gzip –trimns*" parameters.[59] The libraries, sequenced by paired-end reads were merged after removing residual adapter sequences, requiring at least 11 bp overlap between the pairs using additionally "*–collapse –minalignmentlength 11*". The merged reads were mapped to the human reference genome (version hs37d5) using the program "*BWA aln/samse*" (version 0.7.15)[60] with parameters "*-n 0.01, -o 2*" and by disabling the seed with "*-l 16500*."[61]

Multiple libraries from the same individual were merged with "*samtools merge*" (version 1.9)[62] and PCR duplicates with identical start and end coordinates were removed using "*FilterUniqueSAMCons.py*."[61] Reads with >10% mismatches to the human reference genome, <35 base pairs and <30 mapping quality were also removed.

We should note that the samples that had both UDG and non-UDG libraries were merged after trimming at the ends of the reads (see below). Consequently, we do not report PMD damage for those merged libraries in Table S1. All library-specific PMD-damage profiles are available in Table S1.

We calculated average genome coverage, including only reads with mapping quality >30 (see Table S1), and using "*genomeCoverageBed*" implemented in "*bedtools2*."[69] All samples had genome coverages >0.02X.

The previously published ancient genomic data were also remapped and filtered using the same procedure to avoid biases.

Testing for contamination and quality control

To evaluate the authenticity of the genomes, we used three approaches after extracting the reads of minimum base quality and mapping quality of 30 for each sample: (1) examination of all ancient DNA-specific damage patterns caused by cytosine deamination in all samples by using "*PMDtools*" (version 0.60) with the "*–deamination*" parameter,[45] (2) mtDNA-based contamination estimation across all samples by using "*contamMix*" (version 1.0-10),[70] (3) X-chromosome-based contamination estimation of XY samples by using the "*contamination.R*" script in "*ANGSD*" (version 0.937)[63] (Table S1).

All merged libraries showed the following characteristics: (1) $\geq$15% C$\rightarrow$T transitions for non-UDG-treated samples and $\geq$5% C$\rightarrow$T transitions for UDG-treated samples at the first position of 5' end, (2) >93% authenticity estimates based on *contamMix*, and (3) <5% contamination estimates based on X-chromosome contamination estimates in XY samples.

Molecular sex determination

After extracting the reads of minimum base quality and mapping quality of 30, we used both the "R_y"[140] and "R_x" methods[138] to determine the molecular sex of all samples (Tables 1, S1, and S2).

Estimating uniparental haplogroups

Mitochondrial DNA and Y chromosome VCF files were generated from whole genome alignment data (BAM files) using "*samtools mpileup*" (version 1.9) and "*bcftools call*" (version 1.9).[71] Nucleotides with quality scores lower than 30 and depths lower than 2 are filtered by "*bcftools filter*" (version 1.9).[71] mtDNA haplogroups were obtained using "*HaploGrep*" (version 2.4.0)[64] based on build 17 of PhyloTree (http://www.phylotree.org/) and applying a mtDNA quality score threshold >0.5. Y chromosome haplogroups were determined for all male samples by using "*Yhaplo*" (version 1.1.2) based on Y-DNA Haplogroup Tree 2019-2020 of ISOGG (https://isogg.org/) (see Table 1 and Table Z8 in Zenodo).

Both mtDNA and Y-chromosome haplogroups were assigned to major haplogroups. Major haplogroups that were inconsistent with previously published results and haplogroups that cannot be differentiated at high resolution (i.e. Y haplogroup CT) were removed from the analysis. In total 380 mtDNA and 200 Y-chromosome haplogroups were analysed. To assign major haplogroups of mtDNA, U groups either by using just U as a major group or by separating them as U2'3'4'7'8'9 (referred to as U*), U1, U7 and U8 mtDNA and Y-chromosome frequency haplogroup differences between periods were calculated by the pairwise F_{ST} test. F_{ST} values and possible significant deviations from "0" were calculated in "*Arlequin*" (version 3.5) with 10,000 permutations.[74] We applied the false discovery rate (FDR) correction[141] for multiple testing by using R (https://www.r-project.org/) (see also Figure 6A, Figure XXI-XXIII in Document Z1 and Table Z8 in Zenodo).

Genome-wide SNP datasets

We prepared three datasets (panels) for different population genetics analyses.

Dataset 1

The 1000 Genomes sub-Saharan African dataset, which we created in this study as a high-quality and relatively unbiased SNP dataset to use in demographic inference in our sample (following Skoglund et al.[45]). Our motivation was that SNP panels such as the Human Origins or 1240K panel have been mostly ascertained in selected populations, often west Eurasians, and thus suffer from ascertainment bias.[142,143] To avoid this as much as possible, while maintaining a large number of SNPs for statistical power, we prepared this new SNP panel. Importantly, because the SNPs are ascertained in outgroup populations that are equally distant to all studied populations, we can directly interpret changes in diversity as admixture, instead of population size changes (Methods S1A).

This new SNP panel includes both autosomal and X-chromosome SNPs. We started with all bi-allelic SNPs in the 1000 Genomes Project phase 3 dataset.[30] We then masked the following sites as in Tucci et al.[144] with some modifications.

- within 5 bp of another SNPs, a short insertion or deletion;
- within structural variants defined in in phase 3 of the 1000 Genomes project;
- within segmental duplications (downloaded from: http://hgdownload.cse.ucsc.edu/goldenPath/hg19/database/genomicSuperDups.txt.gz);
- within a CpG dinucleotide context;
- not within the 1000 Genomes accessibility mask (downloaded from ftp://ftp.1000genomes.ebi.ac.uk/vol1/ftp/release/20130502/supporting/accessible_genome _masks/20141020.pilot_mask.whole_genome.bed);
- within blacklisted signal artefact regions (downloaded from http://mitra.stanford.edu/kundaje/akundaje/release/blacklists/hg19-human/wgEncodeHg19ConsensusSignalArtifactRegions.bed.gz) except for those within "High_Mappability_island";
- with minor allele frequency (MAF) <5% in all of the 5 sub-Saharan African populations in phase 3 of the 1000 Genomes project: Yoruba in Ibadan, Nigeria; Luhya in Webuye, Kenya; Gambian in Western Divisions in the Gambia; Mende in Nigeria; Esan in Nigeria (504 individuals in total);
- with Hardy-Weinberg equilibrium exact test p-value below the 0.001 in each of the 5 sub-Saharan African populations in phase 3 of the 1000 Genomes project;
- within pseudoautosomal regions in the X chromosome.

After filtering, 4,771,930 autosomal and 206,805 X chromosome SNPs remained. Note that these had >5% MAF in at least one of the five sub-Saharan African populations in phase 3 of the 1000 Genomes project.

We genotyped all ancient individuals in our dataset (Table S2) at these SNP positions (see below) and merged this data with genotype data from 300 present-day samples from Mallick et al.[145] (downloaded from https://reichdata.hms.harvard.edu/pub/datasets/sgdp/ as "*PLINK format*" on 30 Aug 2020).

The corresponding genotype data can be found in Dataset1.zip file in Zenodo.

Dataset 2

The 1240K Capture Array dataset includes 417 ancient and 23 present-day individuals from SW Asia called across a total of 1,121,751 autosomal SNPs.[31]

Dataset 3

The Human Origins SNP Array (HO) dataset includes 2,583 present-day humans from 213 different populations genotyped on the Affymetrix Human Origins Array,[11,146] merged with newly generated and previously published ancient individuals (see below), and 300 present-day samples from Mallick et al.[145] (downloaded from https://reichdata.hms.harvard.edu/pub/datasets/sgdp/ as "*PLINK format*" on 30 Aug 2020) as well as 763 present-day samples from Jeong et al.[147] (downloaded from https://edmond.mpdl.mpg.de/file.xhtml?fileId=101735&version=1.0 as "*EIGENSTRAT format*" on 30 Aug 2020" on a total of 615,771 autosomal SNPs.

We used the Human Origins SNP Array dataset (Dataset 3) for PCA analyses, the 1240K Capture Array dataset (Dataset 2) to estimate Runs of Homozygosity (ROH) using hapROH, and the 1000 Genomes sub-Saharan African dataset (Dataset 1) for all other analyses.

Ancient genome sample selection

In our dataset, we included all published ancient samples (as of April 2022) from Anatolia,[9,11,12,14,18,20,22,31,40,148] the Aegean,[13,18,31,40] present-day Iran,[11,19,149] South Caucasus,[11,12,20,21,150,151] and the Levant.[8,11,14–16,20,24,44] We also added 23 present-day individuals from the same five regions.[145] In addition, we included genomes from European Hunter-Gatherers,[12,31,146,152,153] Baikal Neolithic and Bronze Age (Damgaard et al.[12]), West Siberian Hunter-Gatherers[19] as well as Yamnaya[12,19,31,150,153] populations to analyze their relationships with Southwest Asian and East Mediterranean populations. If there were either first- or second-degree related individuals from the same site, we retained the highest coverage genome and excluded the rest from the dataset. We did not include the Ashkelon samples (n=10) from Feldman et al.[26] due to their low number of SNPs (< 2000 SNPs) overlapping with the 1000 Genomes sub-Saharan African dataset, and also Ash002 and Ash040 from Yaka et al.[22] due to the relatively high genetic similarity to each other as measured in their outgroup f_3-scores ($f_3 > 0.30$).

We also added two Aegean Mesolithic mtDNA genomes (Theo1 and Theo5) from Hofmanova et al.[40] to mtDNA haplogroup analyses.

Ancient genomes and ancient genome groups (so-called "populations") were named following the groups defined in the original publications. The only exception was the MA2198 genome from Damgaard et al.[12] The C14 date of MA2198 was reported earlier as the Ottoman period by Omori and Nakamura,[154] while the Iron Age date mentioned in the article appears to be a mistake. This sample was accordingly named Anatolia_Kalehoyuk_OttomanIII (see also Table S2).

Defining time periods

Instead of grouping populations based on archaeological periodization, we decided to use temporal groups because of the difficulty in assigning matching cultural identities across regions. We thus chose the divide our time range into six time periods (TP): TP1 (>= 10,000 BP], TP2 (10,000 - 8000 BP], TP3 (8000 - 6000 BP], TP4 (6000 - 4000 BP], TP5 (4000 - 2000 BP] and TP6 (2000 BP - present]. We also tested the same approach but using 1,000-year or 2,500-year windows instead of 2,000-year windows, after 10,000 BP (see Figures XIII and XIV in Document Z1).

Trimming and pseudo-haploid genotyping

To avoid possible confounding by deamination (C-to-T and G-to-A transitions) at the ends of the reads, we trimmed (a) 10 bases at the ends of each read in libraries obtained by shotgun sequencing without Uracil-DNA-glycosylase (UDG) treatment, and (b) 2 bases at the ends of each read from libraries obtained with UDG treatment. When the sample had both UDG and non-UDG libraries, we trimmed each before merging the libraries. Trimming (clipping) was performed using the "*trimBam*" command of "*bamUtil*" (version 1.0.14).[75] To avoid genotype calling biases due to differential sequencing coverage among samples, we pseudo-haploidized the data by randomly selecting one allele for each of the targeted SNP positions using the genotype caller "*pileupCaller*" (version 1.2.2) (https://github.com/stschiff/sequenceTools) on "*samtools mpileup*" output (base quality>30 and MAPQ>30).[62]

Genetic kinship analyses

We used "*READ*"[66] to determine genetic kinship between each pair of individuals from the same site using the 1000 Genomes sub-Saharan African SNP panel dataset (Dataset 1) and pseudo-haploidized genotypes. First, we ran *READ* to calculate pairwise mismatch rates (P0) for each 1 Mb window. For each population from different regions and time periods, we computed the P0 value separately by using all published and unpublished neighboring contemporary samples, except for Shah Tepe, which had a sufficient number of samples (n=9) for this analysis. To calculate a robust normalization factor (median of P0 values for each population), we took into account only pairs that had more than 5,000 overlapping SNPs. We then calculated kinship coefficient (θ) for each window using (1 - normalized P0) as a proxy utilizing custom script. Finally, we computed the mean θ value for each pair of individuals (see Figure I in Document Z1 and Table Z9 in Zenodo).

Two pairs from Perachora, Greece (Library ID: 62_1 and Library ID: 62_3; Library ID: G76a and Library ID: 62_2; see Table S1) were identified as "Identical/Twin" by *READ*. The libraries obtained from 62_1 and 62_3 were constructed from left-side and right-side petrous bones, respectively, which would be consistent with the possibility that the bones were derived from a single individual. The libraries obtained from G76a and 62_2 were constructed from a petrous bone and a tooth (see Table S1). They may be either the same individual or twins, although with Perachora Cave being an ossuary, the former is more likely. We thus merged BAM files of these two pairs as G62 and G76a, respectively, and treated them as two individuals in downstream analyses. All other samples were unrelated (see Figure I in Document Z1, Table S1 and Table Z9 in Zenodo).

Principal components analysis (PCA)

We performed principal components analysis[65] to obtain an overview of the possible relationships among populations and/or possible artefacts. We used the "*smartpca*" program (version: 18140) of "*EIGENSOFT*" (version 7.2.0)[65] with "*lsqproject:YES, numoutlieriter: 0*" parameters to construct the components of present-day West Eurasian or Eurasian populations from Human Origins SNP Array dataset (Dataset 3) (see Table Z1 in Zenodo). Ancient individuals were projected onto the first two principal components of present-day genetic variance (see Figure 2; Figure Z1-2, and Figures II–IV in Document Z1).

Ancestry proportion estimation

We estimated proportions of ancestry in SW Asia populations using "*qpAdm*" (version: 1520)[33,34] implemented in "*AdmixTools*" (version 7.0.2), with "*allsnps: YES, details:YES*" parameters. For all runs, we used a base set of "Right" populations (Base12) composed of Mbuti, Han, Papuan, Mixe, Ust_Ishim, Kostenki14, MA1, Villabruna, Levant_HG, Anatolia_HG, Iberomaurusian, AfontovaGora3, plus either CHG (Base12_CHG) or Iran_GanjDareh_N (Base12_Iran) (13 in total).[14,145,146,152,155,156] To model Anatolia Ottoman individuals, we also added Botai_EN to the right populations (Base12_Iran_Botai, Base12_CHG_Botai) (14 in total). In choosing "Right" populations we followed former studies[14,20,35] with some modifications to improve resolution.

We generated input files for qpAdm analysis that included all combinations of target, source and right populations by using qpAdm-wrapper (https://github.com/dkoptekin/qpAdm-wrapper).

The criteria used to report the results in Figure 3 were as follows: (a) To model populations for each region, we chose the *earliest* Holocene component from Southwest Asia as the primary source, if such models were feasible. (b) For possible external ancestry sources we used in the models, we chose those populations that could explain (produce feasible models with) the majority of the populations in that region (e.g. to explain Anatolian-related ancestry in S Caucasus we used both Neolithic and Chalcolithic populations from Anatolia in our models. We found that more populations can be modelled with Anatolian Chalcolithic populations than with Neolithic populations. Therefore, we reported models of S Caucasus with Anatolian Chalcolithic populations in Figure 3). (c) Among feasible models, we chose parsimonious models (i.e., with fewer source populations) over more complicated models. (d) If the model of a particular population was feasible (p>0.01) and fit the described criteria a-c, but yielded p<0.05, and if this population could be modelled with a different source with p>0.05, we reported the p>0.05 model.

The results used in Figure 3 are provided in Table S4. All models that we attempted are reported in Table Z3 in Zenodo.

Genetic differentiation among populations

We calculated inter-population differentiation using F_{ST}, separately for regional populations in each time period (Figure 1, Table II in Document Z1). We used the "*smartpca*" algorithm (version: 18140) of "*EIGENSOFT*" (version 7.2.0),[65] with parameters "*inbreed:YES, fstonly: YES*". We used the Z > 3 cut-off for each comparison, representing nominally significant p<0.001 (see Figure 5 and Table Z4 in Zenodo).

Genomic similarity/distance among populations

Genome-wide similarity was calculated using outgroup-f_3 statistics[32] implemented in the "*qp3Pop*" algorithm (version: 651) in "*AdmixTools*" (version 7.0.2). We used genomes from all populations (*i.e.,* all sites and periods), using the 1000 Genomes phase3 Yoruba population (n=108) as an outgroup. We used 1-f_3 as a measure of genetic distance. We used >2,000 SNPs as cut-off for calculations for autosomal SNPs and >1,000 SNPs for X chromosome SNPs (see Figures 4 and 5 and Table Z5-7 in Zenodo).

Detecting gene flow among populations

To estimate gene flow between Population X and Population Y for autosomes, we used f_4-statistics[32] implemented in the "*qpDstat*" algorithm (version: 980) in "*AdmixTools*" (version 7.0.2). We used tests of the form f_4(Test, Outgroup; PopX, PopY) using the 1000 Genomes phase3 Yoruba population as an outgroup and with the "*f4mode: YES*" option. We used >10,000 overlapping SNPs as cut-off for reporting f_4-test calculations (see Figures S1–S4; Table S3).

Admixture time estimation

To estimate the admixture date for the Musular and for the Greece Bronze Age genomes, we applied "*DATES*"[36] (https://github.com/priyamoorjani/DATES) using default parameters. We assigned models that yielded a positive mean of <300 generations, a normalized root-mean-square deviation (NRMSD) <1 and Z-score >2 as "feasible". We assumed 28 years per generation (Table IV in Document Z1).

Runs of homozygosity (ROH)

We estimated ROH using "*hapROH*" (version 0.3a4)[46] with default parameters that were optimized for the 1240K SNPs. The default genetic map of hapROH and 5,008 global haplotypes from the 1000 Genomes Project were used.[30] We used 104 of the 440 ancient and modern-day SW Asian genomes which were covered by at least 400K SNPs of the 1240K Capture Array dataset (Dataset 2). We plotted the values of the sum of ROH (4-8 cM) in time transects for Anatolia, Aegean, modern-day Iran, Levant, and South Caucasus (see Figure S5).

Coalescent simulations

We chose to use population genetic simulations under specific demographic models to effectively interpret the different behaviors of the F_{ST} and f_3 statistics. Using simulations effectively allows us to compare these statistics, as we fully know the demographic processes behind the data. We performed coalescent simulations using the software "*msprime*" (version 0.7.4)[76] under four various demographic models involving four or five populations. We assumed a mutation rate of 1.25×10^{-8} bp yr^{-1}, and a recombination rate of 1.0×10^{-8} bp yr^{-1} and 29 years per generation.[146]

For all models, we sampled 100 Mbp DNA sequences for 100 representatives of a simulated population that would stand for present-day Yoruba individuals (N_e = 100,000), representing the outgroup, and 10 individuals of PopA, PopB, PopC, and PopX (N_e = 10,000). For the four population models, the tree topology used was in the form of {YRI, {PopA, {PopB, PopC}}}, and the respective divergence times were 160,000 BP, 40,000 BP and 20,000 BP. For the five population models, the tree topology used was in the form of {YRI, {PopX, {PopA {PopB, PopC}}}}, and the respective divergence times were 160,000 BP, 70,000 BP, 40,000 BP and 20,000 BP. The models are described in the legend of Figure VII in Document Z1. We computed F_{ST}, outgroup-f_3 and pairwise mismatch (pmm) using "*weir_cockerham_fst*", "*patterson_f3*", "*mean_pairwise_difference_between*" functions, respectively, from the "*scikit-allel*" Python package (version 1.3.2) (https://scikit-allel.readthedocs.io/). See Figure VII in Document Z1 and Table Z2 in Zenodo for the different parameters used.

Visualization

We produced all graphs in R (https://www.r-project.org/) after reading and manipulating data using "*tidyverse*,"[157] "*plyr*,"[158] "*reshape2*,"[159] and "*gsheet*"[160] packages. All figures produced by using "*ggplot2*"[161] and its extension packages such as "*ggtext*,"[162] "*ggforce*,"[163] "*ggpubr*,"[164] "*ggrepel*."[165] In Figure 1, we used freely available Natural Earth data (https://www.naturalearthdata.com) to create the map by using "*maps*,"[166] "*raster*"[167] and "*rgdal*"[168] packages. The multiple panel figures combined by using "*patchwork*"[169] package.

Current Biology, Volume *33*

Supplemental Information

Spatial and temporal heterogeneity in human mobility patterns in Holocene Southwest Asia and the East Mediterranean

Dilek Koptekin, Eren Yüncü, Ricardo Rodríguez-Varela, N. Ezgi Altınışık, Nikolaos Psonis, Natalia Kashuba, Sevgi Yorulmaz, Robert George, Duygu Deniz Kazancı, Damla Kaptan, Kanat Gürün, Kıvılcım Başak Vural, Hasan Can Gemici, Despoina Vassou, Evangelia Daskalaki, Cansu Karamurat, Vendela K. Lagerholm, Ömür Dilek Erdal, Emrah Kırdök, Aurelio Marangoni, Andreas Schachner, Handan Üstündağ, Ramaz Shengelia, Liana Bitadze, Mikheil Elashvili, Eleni Stravopodi, Mihriban Özbaşaran, Güneş Duru, Argyro Nafplioti, C. Brian Rose, Tuğba Gencer, Gareth Darbyshire, Alexander Gavashelishvili, Konstantine Pitskhelauri, Özlem Çevik, Osman Vuruşkan, Nina Kyparissi-Apostolika, Ali Metin Büyükkarakaya, Umay Oğuzhanoğlu, Sevinç Günel, Eugenia Tabakaki, Akper Aliev, Anar Ibrahimov, Vaqif Shadlinski, Adamantios Sampson, Gülşah Merve Kılınç, Çiğdem Atakuman, Alexandros Stamatakis, Nikos Poulakakis, Yılmaz Selim Erdal, Pavlos Pavlidis, Jan Storå, Füsun Özer, Anders Götherström, and Mehmet Somel

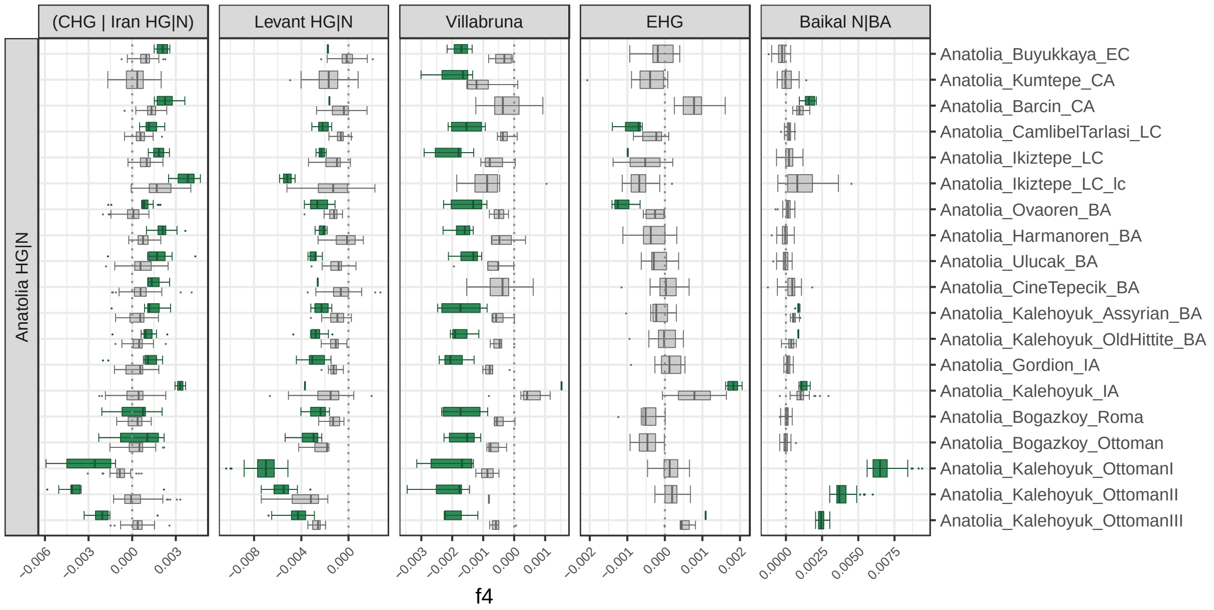

Figure S1: Genetic affinities of post-Neolithic Anatolian populations, **related to Table S3-S4 and Figure 3**. Results from the model *f_4(YRI, Test; Anatolia HG|N, Post Neolithic Anatolian)*, where *Test* refers to the South Caucasus/Iran or Levant pre-Neolithic/Neolithic populations, Villabruna, EHG and Baikal populations (Table S3). The Dataset 1 (STAR Methods) was used in the analysis. Boxplots show all f_4 statistics calculated by at least 10K overlapping SNPs. Green colour boxplots show nominally significant f_4-statistics with $|Z| > 3$ and grey colour boxplots show non-significant f_4-statistics with $|Z| < 3$.

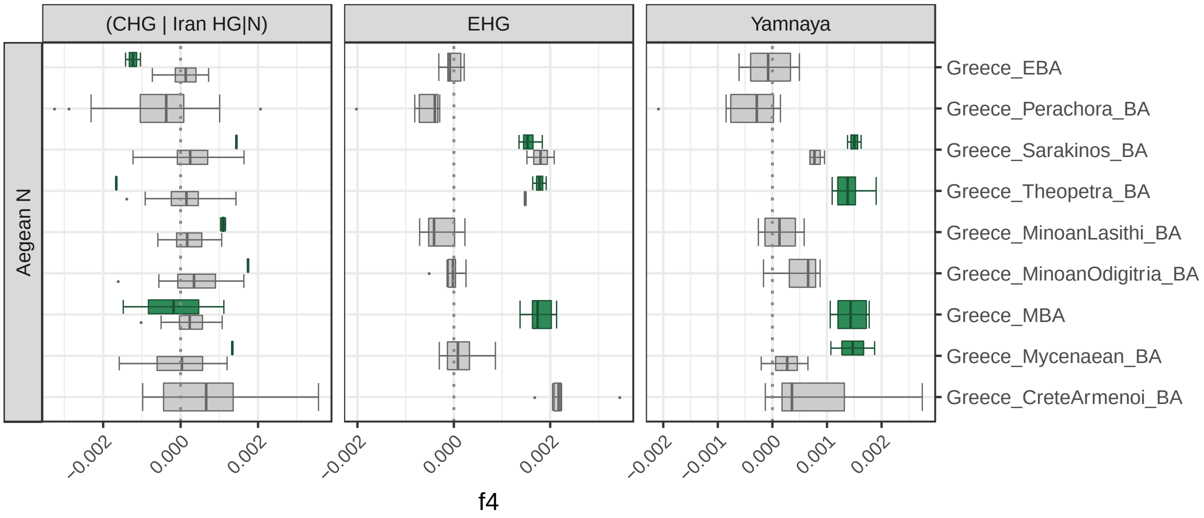

Figure S2: Genetic affinities of post-Neolithic Aegean populations, related to Table S3-S4 and Figure 3. Results from the model f_4*(YRI, Test; Aegean N, Post Neolithic Aegean)*, where *Test* refers to the South Caucasus/Iran pre-Neolithic/Neolithic populations, EHG and Yamnaya populations (Table S3). The Dataset 1 (STAR Methods) was used in the analysis. Boxplots show all f_4 statistics calculated by at least 10K overlapping SNPs. Green colour boxplots show nominally significant f_4-statistics with $|Z| > 3$ and grey colour boxplots show non-significant f_4-statistics with $|Z| < 3$.

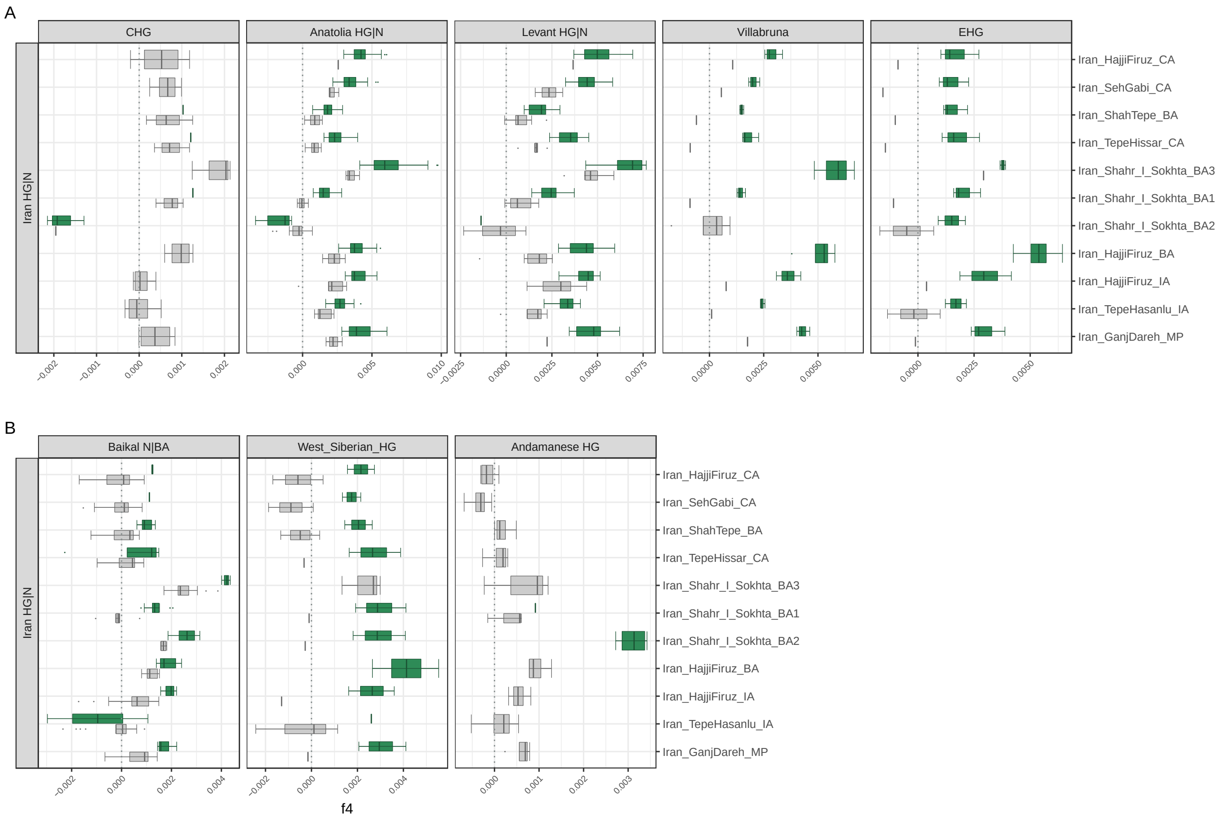

Figure S3: Genetic affinities of post-Neolithic Iran populations, related to Table S3-S4 and Figure 3. Results from the model *f_4(YRI, Test; Iran HG|N, Post Neolithic Iran)*, where *Test* refers to (A) the Anatolian or Levant pre-Neolithic/Neolithic populations, CHG, EHG and Villabruna populations, and (B) Baikal, WSHG and Andamanese HG (Table S3). The Dataset 1 (STAR Methods) was used in the analysis. Boxplots show all f_4 statistics calculated by at least 10K overlapping SNPs. Green colour boxplots show nominally significant f_4-statistics with $|Z| > 3$ and grey colour boxplots show non-significant f_4-statistics with $|Z| < 3$.

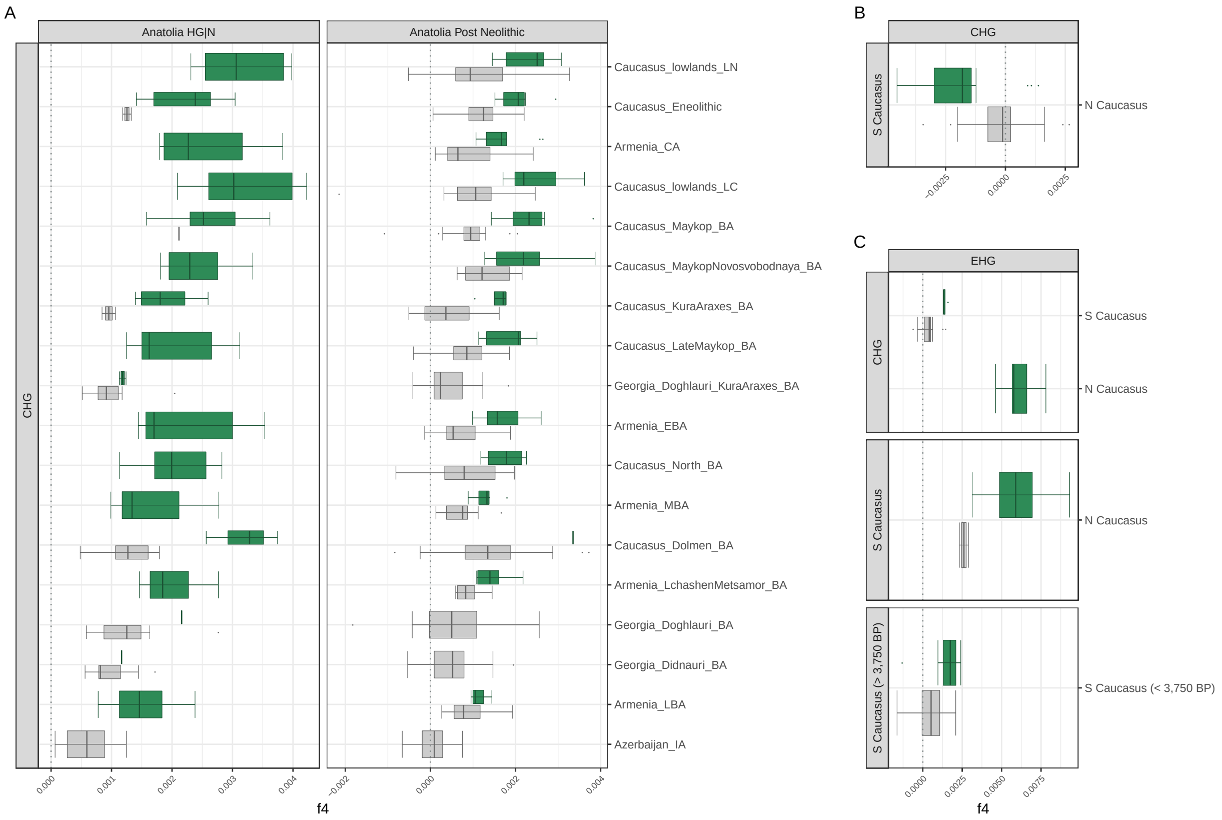

Figure S4: Genetic affinities of post-Neolithic South Caucasus populations, related to Table S3-S4 and Figure 3. Results from (A) *f_4(YRI, Anatolia HG|N or Anatolia Post Neolithic; CHG, South Caucasus Post Neolithic)*, (B) *f_4(YRI, CHG; South Caucasus, North Caucasus)*, and (C) *f_4(YRI, EHG; Pop1, Pop2)*, where *Pop1* or *Pop2* refer to CHG, South Caucasus or North Caucasus populations. The Dataset 1 (STAR Methods) was used in the analysis. Boxplots show all f_4 statistics calculated by using at least 10K overlapping SNPs. Green colour boxplots show nominally significant f_4-statistics with $|Z| > 3$ and grey colour boxplots show non-significant f_4-statistics with $|Z| < 3$.

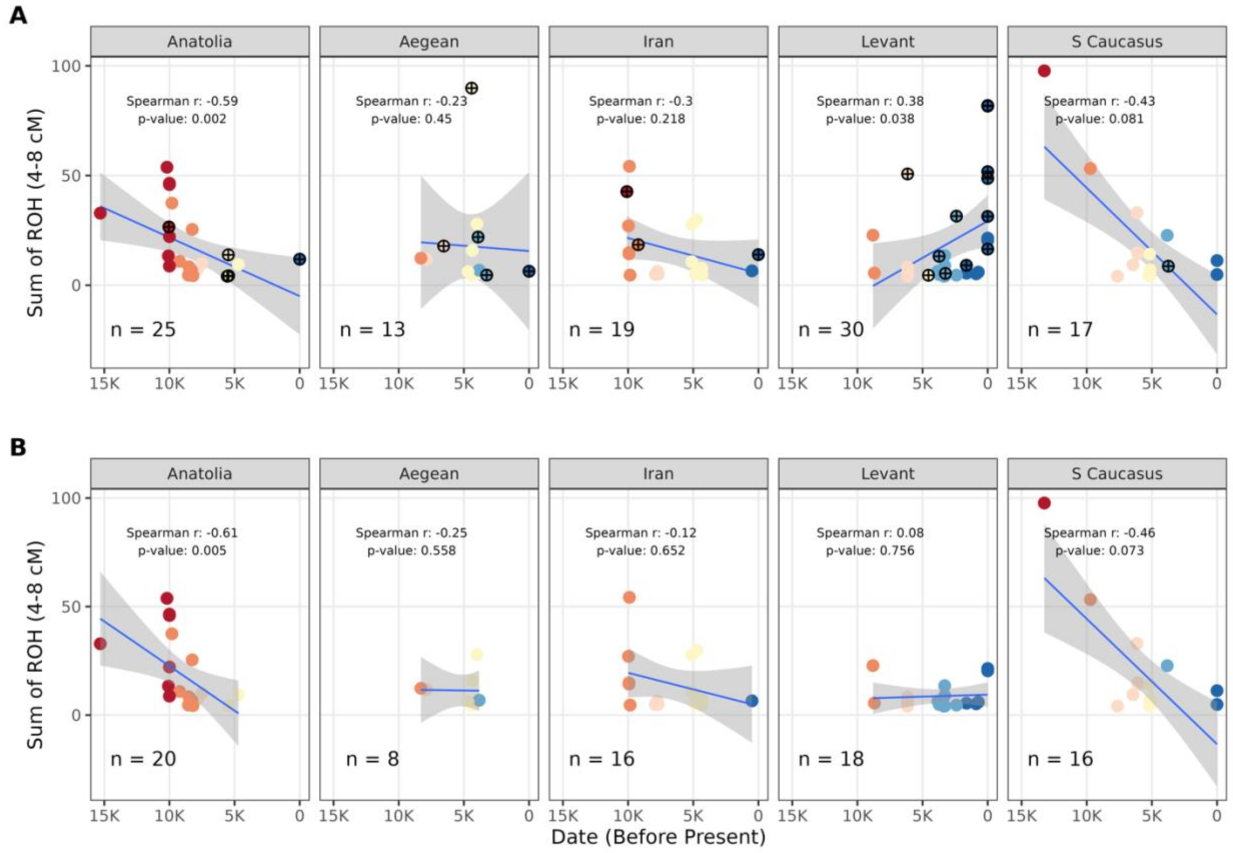

Figure S5: Sum of short (4-8 cM) runs of homozygosity (sROH) among ancient genomes, related to STAR Methods. Panel A shows all individuals for which ROH could be called (with minimum 400,000 SNPs), where individuals with ROH > 20cM, representing consanguinity, are indicated with a plus symbol. Panel B is the same as panel A but after removing likely consanguineous individuals (with ROH > 20cM). The Dataset 2 (STAR Methods) was used in the analysis. The lines in each panel represent linear regression lines. Each point represents an ancient individual and is colour coded based on its time period (Figure 1). We note that we detect no decrease in the sROH values (4-8 cM) for the Aegean, but this is likely because we did not have Aegean genomic samples from the first half of the Holocene with sufficient SNPs to determine ROH. The increase of the sum of ROH in the Levant we observed has been well-documented before and is likely due to an increase in consanguineous marriages in the region in recent times[S1] (see also Table Z11 in Zenodo).

Supplemental References

S1. Scott, E.M., Halees, A., Itan, Y., Spencer, E.G., He, Y., Azab, M.A., Gabriel, S.B., Belkadi, A., Boisson, B., Abel, L., et al. (2016). Characterization of Greater Middle Eastern genetic variation for enhanced disease gene discovery. Nat. Genet. *48*, 1071–1076. 10.1038/ng.3592.

S2. Ceballos, F.C., Gürün, K., Altınışık, N.E., Gemici, H.C., Karamurat, C., Koptekin, D., Vural, K.B., Mapelli, I., Sağlıcan, E., Sürer, E., et al. (2021). Human inbreeding has decreased in time through the Holocene. Curr. Biol. *31*, 3925–3934. 10.1016/j.cub.2021.06.027.

S3. Feldman, M., Gnecchi-Ruscone, G.A., Lamnidis, T.C., and Posth, C. (2021). Where Asia meets Europe – recent insights from ancient human genomics. Ann. Hum. Biol. *48*, 191–202. 10.1080/03014460.2021.1949039.

S4. Harney, É., May, H., Shalem, D., Rohland, N., Mallick, S., Lazaridis, I., Sarig, R., Stewardson, K., Nordenfelt, S., Patterson, N., et al. (2018). Ancient DNA from Chalcolithic Israel reveals the role of population mixture in cultural transformation. Nat. Commun. *9*, 3336. 10.1038/s41467-018-05649-9.

S5. Kılınç, G.M., Omrak, A., Özer, F., Günther, T., Büyükkarakaya, A.M., Bıçakçı, E., Baird, D., Dönertaş, H.M., Ghalichi, A., Yaka, R., et al. (2016). The demographic development of the first farmers in Anatolia. Curr. Biol. *26*, 2659–2666. 10.1016/j.cub.2016.07.057.

S6. Marchi, N., Winkelbach, L., Schulz, I., Brami, M., Hofmanová, Z., Blöcher, J., Reyna-Blanco, C.S., Diekmann, Y., Thiéry, A., Kapopoulou, A., et al. (2022). The genomic origins of the world's first farmers. Cell *185*, 1842-1859.e18. 10.1016/j.cell.2022.04.008.

S7. Yaka, R., Mapelli, I., Kaptan, D., Doğu, A., Chyleński, M., Erdal, Ö.D., Koptekin, D., Vural, K.B., Bayliss, A., Mazzucato, C., et al. (2021). Variable kinship patterns in Neolithic Anatolia revealed by ancient genomes. Curr. Biol. *31*, 2455–2468. 10.1016/j.cub.2021.03.050.

S8. Skoglund, P., Malmström, H., Omrak, A., Raghavan, M., Valdiosera, C., Günther, T., Hall, P., Tambets, K., Parik, J., Sjögren, K.-G., et al. (2014). Genomic diversity and admixture differs for Stone-Age Scandinavian foragers and farmers. Science *344*, 747–750. 10.1126/science.1253448.

S9. Lazaridis, I., Nadel, D., Rollefson, G., Merrett, D.C., Rohland, N., Mallick, S., Fernandes, D., Novak, M., Gamarra, B., Sirak, K., et al. (2016). Genomic insights into the origin of farming in the ancient Near East. Nature *536*, 419–424. 10.1038/nature19310.

S10. Nielsen, R., and Slatkin, M. (2013). An introduction to population genetics. Sunderland MA Sinauer Assoc. Sch.

S11. Harney, É., Patterson, N., Reich, D., and Wakeley, J. (2021). Assessing the performance of qpAdm: a statistical tool for studying population admixture. Genetics *217*, iyaa045. 10.1093/GENETICS/IYAA045.

S12. de Barros Damgaard, P., Marchi, N., Rasmussen, S., Peyrot, M., Renaud, G., Korneliussen, T., Moreno-Mayar, J.V., Pedersen, M.W., Goldberg, A., Usmanova, E., et al.

(2018). 137 ancient human genomes from across the Eurasian steppes. Nature *557*, 369–374. 10.1038/s41586-018-0094-2.

S13. Clemente, F., Unterländer, M., Dolgova, O., Amorim, C.E.G., Coroado-Santos, F., Neuenschwander, S., Ganiatsou, E., Cruz Dávalos, D.I., Anchieri, L., Michaud, F., et al. (2021). The genomic history of the Aegean palatial civilizations. Cell *184*, 2565–2586. 10.1016/j.cell.2021.03.039.

S14. Feldman, M., Fernández-Domínguez, E., Reynolds, L., Baird, D., Pearson, J., Hershkovitz, I., May, H., Goring-Morris, N., Benz, M., Gresky, J., et al. (2019). Late Pleistocene human genome suggests a local origin for the first farmers of central Anatolia. Nat. Commun. *10*, 1218. 10.1038/s41467-019-09209-7.

S15. Haber, M., Doumet-Serhal, C., Scheib, C., Xue, Y., Danecek, P., Mezzavilla, M., Youhanna, S., Martiniano, R., Prado-Martinez, J., Szpak, M., et al. (2017). Continuity and admixture in the last five millennia of Levantine history from ancient Canaanite and present-day Lebanese genome sequences. Am. J. Hum. Genet. *101*, 274–282. 10.1016/j.ajhg.2017.06.013.

S16. Haber, M., Nassar, J., Almarri, M.A., Saupe, T., Saag, L., Griffith, S.J., Doumet-Serhal, C., Chanteau, J., Saghieh-Beydoun, M., Xue, Y., et al. (2020). A genetic history of the Near East from an aDNA time course sampling eight points in the past 4,000 years. Am. J. Hum. Genet. *107*, 149–157. 10.1016/j.ajhg.2020.05.008.

S17. Kılınç, G.M., Koptekin, D., Atakuman, Ç., Sümer, A.P., Dönertaş, H.M., Yaka, R., Bilgin, C.C., Büyükkarakaya, A.M., Baird, D., Altınışık, E., et al. (2017). Archaeogenomic analysis of the first steps of Neolithization in Anatolia and the Aegean. Proc. R. Soc. B *284*, 20172064. 10.1098/rspb.2017.2064.

S18. Lazaridis, I., Mittnik, A., Patterson, N., Mallick, S., Rohland, N., Pfrengle, S., Furtwängler, A., Peltzer, A., Posth, C., Vasilakis, A., et al. (2017). Genetic origins of the Minoans and Mycenaeans. Nature *548*, 214–218. 10.1038/nature23310.

S19. Narasimhan, V.M., Patterson, N., Moorjani, P., Rohland, N., Bernardos, R., Mallick, S., Lazaridis, I., Nakatsuka, N., Olalde, I., Lipson, M., et al. (2019). The formation of human populations in South and Central Asia. Science *365*, eaat7487. 10.1126/science.aat7487.

S20. Skourtanioti, E., Erdal, Y.S., Frangipane, M., Balossi Restelli, F., Yener, K.A., Pinnock, F., Matthiae, P., Özbal, R., Schoop, U.D., Guliyev, F., et al. (2020). Genomic history of Neolithic to Bronze Age Anatolia, Northern Levant, and Southern Caucasus. Cell *181*, 1158–1175. 10.1016/j.cell.2020.04.044.

S21. Wang, C.-C., Reinhold, S., Kalmykov, A., Wissgott, A., Brandt, G., Jeong, C., Cheronet, O., Ferry, M., Harney, E., Keating, D., et al. (2019). Ancient human genome-wide data from a 3000-year interval in the Caucasus corresponds with eco-geographic regions. Nat. Commun. *10*, 590. 10.1038/s41467-018-08220-8.

S22. Mehrjoo, Z., Fattahi, Z., Beheshtian, M., Mohseni, M., Poustchi, H., Ardalani, F., Jalalvand, K., Arzhangi, S., Mohammadi, Z., Khoshbakht, S., et al. (2019). Distinct genetic variation and heterogeneity of the Iranian population. PLoS Genet. *15*, e1008385. 10.1371/journal.pgen.1008385.

S23. Agranat-Tamir, L., Waldman, S., Martin, M.A.S., Gokhman, D., Mishol, N., Eshel, T., Cheronet, O., Rohland, N., Mallick, S., Adamski, N., et al. (2020). The genomic history of the Bronze Age Southern Levant. Cell *181*, 1146–1157. 10.1016/j.cell.2020.04.024.

S24. Haber, M., Mezzavilla, M., Xue, Y., and Tyler-Smith, C. (2016). Ancient DNA and the rewriting of human history: be sparing with Occam's razor. Genome Biol. *17*, 1. 10.1186/s13059-015-0866-z.

S25. Feldman, M., Master, D.M., Bianco, R.A., Burri, M., Stockhammer, P.W., Mittnik, A., Aja, A.J., Jeong, C., and Krause, J. (2019). Ancient DNA sheds light on the genetic origins of early Iron Age Philistines. Sci. Adv. *5*, eaax0061. 10.1126/sciadv.aax0061.

S26. Altınışık N.E., Kazancı D.D., Aydoğan A., Gemici H.C., Erdal Ö.D., Sarıaltun S., et al. A genomic snapshot of demographic and cultural dynamism in Upper Mesopotamia during the Neolithic Transition. Sci. Adv. 2022;8:eabo3609. doi:10.1126/sciadv.abo3609
